# Growth-Directing Nanostructured Interfaces via Block Copolymer-Templated Au Nanoseed Arrays for Stabilized Zinc Anodes in Lean-Zinc Batteries

**DOI:** 10.1007/s40820-026-02167-y

**Published:** 2026-05-06

**Authors:** Dong Won Hae, Hoseok Lee, Hyeong Jun Kook, Ga Hee Kim, Saehun Kim, Jaecheol Choi, Min Pyeong Kim, Seok Hun Kang, Won Jun Lee, Young-Gi Lee, Hyeong Min Jin, Jongsoon Kim, Dong Ok Shin

**Affiliations:** 1 Smart Materials Research Section, Electronics and Telecommunications Research Institute (ETRI), Daejeon, 34129 Republic of Korea; 2 Department of Semiconductor and Advanced Device Engineering, University of Science and Technology (UST), Daejeon, 34113 Republic of Korea; 3 Department of Future Energy Engineering, Sungkyunkwan University, Suwon, 16419 Republic of Korea; 4 SKKU Institute of Energy Science and Technology (SIEST), Sungkyunkwan University, Suwon, 16419 Republic of Korea; 5 Department of Energy Science, Sungkyunkwan University, Suwon, 16419 Republic of Korea; 6 Department of Fiber System Engineering, Dankook University, Yongin, 16890 Republic of Korea; 7 Department of Organic Materials Engineering, Chungnam National University, Daejeon, 34134 Republic of Korea

**Keywords:** Aqueous zinc-ion battery, Block copolymer template, Au nanoseed, Plated zinc anode

## Abstract

Achieving dendrite-free and highly reversible Zn anodes remains a critical challenge for realizing high-performance aqueous Zn-ion batteries (AZIBs). Here, we report a rationally designed nanoscale interfacial architecture, constructed via sequential assembly of a reduced graphene oxide (rGO) nanolayer and solvent-annealed block copolymer (BCP)-templated Au nanoseed arrays, that enables spatially uniform Zn nucleation and planar plating, yielding highly stable, dendrite-free Zn anodes with minimal excess Zn. The synergistic integration of zincophilic Au nanoseeds on rGO, which energetically favor Zn (002) nucleation, effectively suppresses parasitic reactions and directs preferentially oriented Zn growth, yielding macroscopically uniform Zn plating with an exceptionally high (002) relative texture coefficient (RTC) of 88.2%. Combined experimental and theoretical investigations reveal that the homogeneous Zn^2+^ flux and substrate-induced texture development at the Au nanoseed–rGO interface (AGI) are critical for directing initial Zn nucleation and sustaining planar growth. The plated Zn on AGI (Zn@AGI) delivers exceptional cycling stability (> 3000 h at 1 mA cm^−2^, 10% DOD) and excellent performance under high-DOD conditions. Moreover, full cells incorporating Zn@AGI achieve a remarkably high energy density of 156.1 Wh kg^−1^ full electrode under an ultralow *N*/*P* ratio of 2. This work presents a strategic interfacial approach toward stable and practical AZIBs.

**Supplementary Information:**

The online version contains supplementary material available at 10.1007/s40820-026-02167-y.

## Highlights

- A nanoscale Au nanoseed–rGO interface (AGI) architecture drives highly planar Zn plating with an exceptional (002) texture (RTC = 88.2%), yielding dense and dendrite-free Zn growth.
- The AGI-driven texture regulation homogenizes Zn^2+^ flux, suppresses ZHS/HER side reactions and delivers exceptional cycling durability (>3000 h at 10% depth of discharge).
- A full-cell paired with a high-loading MnO_2_ cathode delivers 156.1 Wh kg^−1^ under an ultralow N/P ratio of 2, highlighting strong practical viability for lean-zinc batteries.

**Supplementary Information:**

The online version contains supplementary material available at 10.1007/s40820-026-02167-y.

## Graphical abstract

**Figure:**
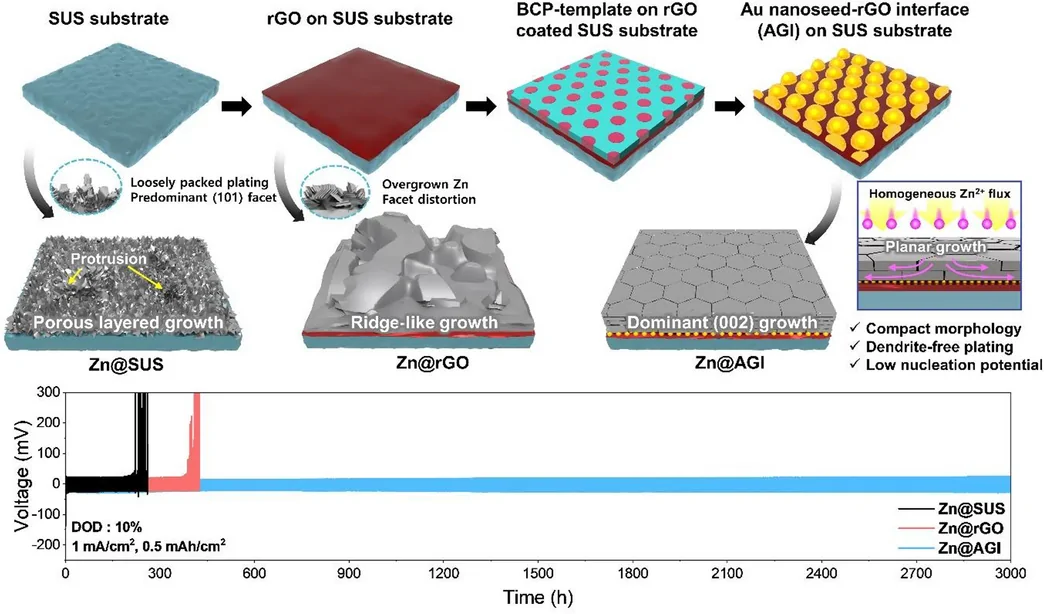

## Introduction

Driven by the growing need for intrinsically safe, economically viable and eco-friendly energy storage platforms, aqueous Zn-ion batteries (AZIBs) have emerged as compelling contenders against Li-ion batteries (LIBs) for future applications in grid storage and portable electronics [1, 2]. Zinc metal is especially attractive due to its natural abundance, high theoretical capacity (820 mAh g^−1^ and 5855 mAh cm^−3^) and suitable redox potential (− 0.76 V vs. standard hydrogen electrode), while water-based electrolytes offer inherent advantages over organic systems, including high ionic conductivity and improved safety [3, 4]. Such combined features render AZIBs suitable for high-rate cycling and safe operation. However, Zn metal, as a non-hosted anode, undergoes dynamic stripping/plating behaviors accompanied by substantial volume changes [5]. As a result, several key challenges, mainly occurring at the structurally fluctuating and reactive surfaces of Zn anodes, remain to be addressed. The most critical issues include the dendritic and uneven Zn growth triggered by “tip effect,” fatal side reactions (e.g., H_2_ generation and Zn corrosion) and inactive oxide passivation, all of which can severely compromise electrochemical efficiency and cycling stability, and, in extreme cases, lead to complete cell failure [6–12]. Moreover, the use of thick zinc foil, which is commonly employed in current AZIB configurations, results in an excessively high *N*/*P* ratio (e.g., > 250 when a 100-μm-thick Zn foil is paired with a 1 mg/cm^2^ MnO_2_ cathode) [13]. This imbalance causes material inefficiency and reduced energy density, ultimately hindering practical applicability.

In pursuit of more stable and efficient Zn anodes, extensive research has been devoted to various engineering strategies. Among them, the surface protection such as Al_2_O_3_ [14], Sn [15], PEO [16], ZnS [17] and MXene [18] has been widely employed and demonstrated to be effective in preventing the Zn anode from direct exposure to aqueous electrolytes, thereby suppressing Zn dendrites and interfacial side reactions. However, significant challenges still remain regarding the formation of smooth, uniformly coated thin layers to minimize resistance caused by extended ion diffusion pathways and enhance the interfacial compatibility with Zn metal [19]. In addition, mechanical stress from deep stripping/plating during repeated cycling can induce cracking or delamination of the protective layers, compromising their long-term effectiveness [20]. Besides these surface protection approaches, other strategies such as electrolyte regulation and functional separator design have also been reported to stabilize Zn anodes, mainly by modifying Zn^2+^ solvation structures or regulating ion transport pathways in the cell. However, these methods typically influence Zn plating indirectly through bulk electrolyte chemistry or intermediate components, rather than directly controlling nucleation and crystallographic growth at the Zn surface [21, 22]. Furthermore, these studies still rely on thick Zn foil, resulting in a high N/P ratio and limiting active material utilization, thereby restricting their practical applicability. In this regard, constructing 3D structured conductive architectures including CNT framework [23], 3D-printed N-doped carbon host [24] and ITO mesh template [25] has been proved to be a feasible host for stable Zn deposition, as the enlarged surface area mitigates local electric field intensity. Although these structural conductive electrodes have shown improvements in stabilizing Zn anodes, they often require intricate preparation processes and still suffer from dendritic and uneven Zn plating due to the lack of zincophilic affinity, an ability to attract and bind Zn [26–28]. Moreover, while adjusting the amount of plated Zn on these 3D architectures can be a viable approach to achieve a desirable *N*/*P* ratio, the inactive host structure itself adds significant weight and thus compromises the overall cell energy density [29].

From a practical perspective, minimizing excessive Zn usage is essential, and this requires plating Zn directly onto current collectors in the form of a uniform and compact morphology. Deliberate introduction of zincophilic sites on Zn plating substrates may offer effective control over Zn anode stabilization by employing seed materials with intended multiple benefits (e.g., high Zn affinity, uniform Zn-ion flux, spatially regulated Zn deposition, low nucleation potential). Particularly, an incorporation of heterogeneous seeds such as Au [30] and Ag [31] on Zn substrates has been explored, showing promising potential to provide preferred nucleation sites and suppress dendrite formation. As a proof of concept, Zhi et al. demonstrated that quasi-isolated nano-Au particles on Zn foil preferentially absorb Zn ions and prevent dendrites/protrusions during repeated plating/stripping of Zn [30]. Liu et al. visualized non-dendritic, seed-mediated Zn deposition on Ag-loaded carbon paper substrates through the formation of Zn_*x*_Ag_1-*x*_ alloy phases [31]. However, plated Zn anodes using zincophilic seeds still suffer from electrochemical side reactions due to their lack of inherent corrosion resistance, ultimately leading to dendritic growth and/or by-product formation under low-rate or high-capacity stripping/plating operations [32, 33]. Moreover, given that the initial Zn nucleation process strongly influences the subsequent growth morphology, achieving a homogeneous distribution of seed sites with strong Zn-ion affinity is a prerequisite for compact and continuous Zn plating, which has rarely been reported. Another critical requirement for stable Zn plating is the regulation of crystallographic growth orientation during Zn plating. It is well known that achieving a preferential (002) orientation of Zn deposition can be a key strategy for suppressing parasitic reactions in AZIBs [34]. The superior stability of the (002) facet can be explained by the Gibbs–Curie–Wulff theorem, which predicts its intrinsically lower surface energy relative to the (001) and (101) facets, thereby mitigating water-induced side reactions and retarding vertical Zn growth [35, 36]. Recently, Archer et al. have showed that a graphene substrate with a low lattice mismatch of ~ 7% to the Zn (002) plane facilitates reversible Zn electroplating and limits dendrite formation [37]. Although these Zn nucleation-guided strategies offer promising pathways for stabilizing Zn metal anodes, the synergistic combined implementation of these strategies has scarcely been reported in scalable and commercially viable AZIB systems operating under practically relevant low N/P ratios.

In this study, we develop a synergistic nanoscale interfacial architecture on a SUS substrate, which functions as a zincophilic, growth-directing platform to enable uniform Zn plating and ensure long-term anode stability under practically relevant conditions, including a low *N*/*P* ratio. To implement this concept, a hybrid interfacial structure composed of block copolymer (BCP)-templated Au nanoseed arrays assembled on the 2D reduced graphene oxide (rGO) nanolayer is suggested as an effective Zn plating interface for highly stable lean-Zn anodes. The fabrication process begins with the formation of the rGO nanolayer on the SUS substrate, which provides a chemically stable base while reducing the surface roughness for subsequent layer assembly. Following this, a BCP thin film was self-assembled to generate well-ordered cylindrical nanoscale domains through solvent-vapor-driven microphase separation. Subsequently, gold precursors were selectively loaded onto these domains and then reduced, resulting in BCP morphology-directed Au nanoseed arrays on the rGO nanolayer. It was found that the Au nanoseed–rGO interface (AGI), featuring a periodic arrangement of Au nanoseed arrays with strong zincophilicity on the rGO nanolayer, enabled spatially controlled Zn nucleation and planar plating with a predominant (002) facet, thereby yielding highly stable, dendrite-free Zn anodes. In addition, the advantageous coupling effects of AGI were validated through morphological and structural characterizations and electrochemical testing, while theoretical simulations were conducted to elucidate the underlying mechanism. The fabricated Zn anodes were named according to their underlying substrates; for example, the Zn anode plated on AGI@SUS is hereafter referred to as Zn@AGI. Consequently, Zn@AGI exhibited highly reliable Zn stripping/plating in a symmetric cell configuration, maintaining consistent cycling performance over 3000 h at 1 mA cm^−2^ and 10% depth of discharge (DOD, 0.5 mAh cm^−2^), highlighting its ultradurable dendrite-suppressing capability. Notably, even under harsher conditions of 2.5 mA cm^−2^ and 50% DOD (2.5 mAh cm^−2^), the Zn symmetric cell based on Zn@AGI maintained stable cycling over 250 h. The assembled Zn@AGI || MnO_2_ full cell with an *N*/*P* ratio of 23 exhibited 4.6-fold higher capacity than the Zn@rGO-based full cell at 1 A g^−1^ after 1200 cycles. Moreover, under an ultralow *N*/*P* ratio of 2, the Zn@AGI|| MnO_2_ full cell delivered a high energy density of 156.1 Wh kg^−1^ (based on the full electrode), markedly outperforming those of conventional cells employing thick Zn anodes and low-mass-loading cathodes, thus demonstrating excellent practical potential.

## Experimental Section

### Material Preparation

Graphene oxide dispersion (< 50 μm, 1wt%) was purchased from GrapheneAll. An asymmetric block copolymer, poly(styrene-*block*-4-vinylpyridine) (PS-*b*-P4VP, molecular weight: 25 kg mol^−1^ for PS, 10 kg mol^−1^ for P4VP), was obtained from Polymer Source Inc. Tetrahydrofuran (THF, > 99%), toluene (> 99%), hydrochloric acid (HCl, > 99.99%, 37wt%) and sulfuric acid (H_2_SO_4_, > 95%) were purchased from Sigma-Aldrich. Gold(III) chloride trihydrate (HAuCl_4_·3H_2_O) was purchased from Strem Chemicals. For zinc-ion battery experiments, zinc sulfate heptahydrate (ZnSO_4_·7H_2_O, > 99%), manganese sulfate (MnSO_4_·H₂O, > 99%) and potassium permanganate (KMnO_4_, > 99%) were purchased from Sigma-Aldrich. Zinc foil (250 μm, > 99.98%) was purchased from Thermo Fisher Scientific.

### Preparation of Au Nanoseed–rGO Interface on SUS Substrate

To fabricate plated Zn anodes, reduced graphene oxide (rGO) nanolayer was first prepared onto stainless steel (SUS) substrates via multiple spin coating cycles, followed by a subsequent two-step thermal reduction process consisting of vacuum annealing at 150 °C for 4 h and rapid thermal annealing (RTA) at 250 °C for 4 h. Next, block copolymer (BCP) thin films were self-assembled on the rGO nanolayer to achieve Au nanoseed arrays. To conduct this, a BCP solution (1 wt%) was prepared by stirring PS-*b*-P4VP in toluene/THF mixtures (80:20, v/v) until full dissolution of the polymers. The 40-nm-thick BCP thin films were formed on rGO nanolayer by spin coating under 5000 rpm for 60 s. Subsequently, the as-spun BCP thin films were placed in a small sealed vessel. After injecting a solvent mixture of toluene and THF (20:80, v/v), the sealed vessel quickly became saturated with solvent vapor, which in turn induced the self-assembly of the BCP films. The volume of solvent mixture and annealing time were carefully controlled to promote the lateral ordering of cylindrical P4VP nanodomains, generating nanopatterned BCP templates. The self-assembled BCP templates were then immersed in a 1 mM HAuCl_4_ aqueous solution containing 0.01 wt% HCl with controlled loading times to enable site-specific electrostatic interactions of AuCl_4_^−^ anions with the protonated pyridine groups in the P4VP nanocylinders. Following that, the Au anion-loaded BCP templates were subjected to oxygen plasma treatment (50 W, O_2_ 50 SCCM) to remove polymer templates and leave periodic Au nanoseed arrays on rGO@SUS substrates (AGI@SUS). The duration of oxygen plasma treatment was carefully controlled to minimize damage to the underlying rGO nanolayer. Finally, thermal treatment was conducted at 250 °C for 1 h using RTA.

### Fabrication of Plated Zn Anodes

To fabricate the plated Zn anodes, electroplating was carried out in a two-electrode configuration, where commercial Zn foil served as the counter electrode and the substrates (pristine SUS, rGO@SUS and AGI@SUS) as the working electrodes. An aqueous solution of 2 M ZnSO_4_ served as the electrolyte, and the interelectrode distance was fixed at approximately 700 μm. The plating current density was systematically adjusted between 1 and 40 mA cm^−2^, and the plating time was controlled to obtain capacities ranging from 1 to 5 mAh cm^−2^. Unless stated otherwise, a current density of 20 mA cm^−2^ was employed as the standard condition throughout the experiments. For an ultralow *N*/*P* ratio testing (*N*/*P* ratio = 2), the Zn plating capacity was reduced to 1.6 mAh cm^−2^.

### Preparation of α-MnO_2_ Cathode

To synthesize α-MnO_2_, 5 mM of MnSO_4_ and 2 mL of 0.5 M H_2_SO_4_ were added to 60 mL of deionized water under continuous stirring. Subsequently, 20 mL of 0.1 M KMnO_4_ solution was slowly introduced into the mixture. The resulting suspension was transferred into a Teflon-lined stainless steel autoclave and subjected to hydrothermal treatment at 120 °C for 12 h. After cooling to room temperature, the precipitate was collected by centrifugation, washed several times with deionized water and dried overnight in a vacuum oven at 90 °C. The cathode slurry was prepared by mixing α-MnO_2_, Super P and PVDF in a weight ratio of 7:2:1 using N-methyl-2-pyrrolidone. The slurry was cast onto Ti foil and dried under vacuum at 100 °C. The typical mass loading of the cathode was ~ 1 mg cm^−2^. For low *N*/*P* ratio testing (*N*/*P* ratio = 2), a water-based binder system comprising carboxymethyl cellulose (CMC) and styrene–butadiene rubber (SBR) was employed. In this case, the slurry was prepared by mixing α-MnO_2_, Super P, CMC and SBR in a weight ratio of 7:2:0.5:0.5, achieving a cathode mass loading 3.8 mg cm^−2^.

### Material Characterization

Zn anodes for morphological characterization were prepared by disassembling the coin cells after Zn plating, followed by rinsing the samples with distilled water and acetone. Surface and cross-sectional morphologies were examined using field-emission scanning electron microscopy (FE-SEM, S4800, Hitachi). Crystallographic orientation was analyzed using X-ray diffraction (XRD, PANalytical) using a Cu Kα radiation source. Surface chemical composition was characterized by X-ray photoelectron spectroscopy (XPS, K-Alpha+, Thermo Fisher Scientific), calibrated using the C 1*s* peak at 284.5 eV. Atomic force microscopy (AFM, Dimension, Bruker) was employed to analyze the surface topography and height distribution of Au nanoseeds and substrate interfaces. The characteristic Debye ring patterns were analyzed using grazing incidence X-ray diffraction (GIXRD, SmartLab, Rigaku Corporation) with a Cu Kα radiation source operated at 45 kV and 200 mA. Confocal laser scanning microscope (CLSM, LSM 980, Carl Zeiss) was used to assess the macroscopic surface roughness of Zn anodes. Raman spectroscopy (FEX, WEVE) with a 532-nm green laser was conducted to quantitatively evaluate the reduction degree of graphene oxide. In situ electrodeposition behavior and surface evolution were monitored using an optical microscope (OM, BX53M, Olympus).

### Computational Details

The geometry of the rGO and molecular species (including − COOH, − OH, H_2_O, H_2_ and − CH_2_OH) were optimized, and its energy was evaluated using the Gaussian 16 suite. All calculations were carried out with spin-unrestricted density functional theory (DFT), employing the Becke–Lee–Yang–Parr (B3LYP) hybrid exchange–correlation functional along with the triple-zeta valence plus polarization (TZVP) basis set. Dispersion interactions were described using Grimme’s DFT-D3 correction. Interfacial models for Zn plating were subsequently constructed on rGO and the Au array (AGI) presenting the (002) surface; Zn plating was performed on this Au (002) facet. The DFT-D3 correction was retained during interface construction to accurately capture van der Waals interactions. The optimized structure obtained from this procedure was subsequently used as input for adsorption and Gibbs free energy calculations, which were performed using the Vienna Ab Initio Simulation Package (VASP), applying the Generalized Gradient Approximation (GGA) with the Perdew–Burke–Ernzerhof (PBE) functional in combination with the projector augmented-wave (PAW) method. A plane-wave cutoff energy of 500 eV was used, and the atomic structures were fully relaxed until the residual forces on all atoms were less than 0.04 eV Å. For Brillouin zone sampling, a Γ-centered 2 × 2 × 1 k-point mesh was employed for all supercell calculations. Adsorption energy for the single-layer Zn overlayer was defined as *E*_ads_ = *E*_(Zn orientation slab + substrate)_ − *E*_(substrate)_ − *E*_(Zn orientation slab)_, where all energies are total energies of fully relaxed structures for the substrate with the Zn monolayer, the pristine substrate (rGO or AGI) and the isolated Zn monolayer constructed in the same supercell. For multi-layer Zn slabs, the interfacial binding energy was defined as *E*_bind_ = *E*_(Zn multi-slab+substrate)_ − *E*_(substrate)_ − *E*_(Zn multi-slab)_. The Zn slab thickness and the interfacial area were kept identical for the rGO and AGI models, allowing a direct comparison of facet-dependent interfacial stabilization. In all calculations, the isolated Zn monolayer and multi-layer Zn slabs were constructed in the same supercell and in-plane lattice as the corresponding interface models and were evaluated using identical computational settings including the same cutoff energy, k-point mesh, dispersion correction and relaxation criteria, ensuring consistent reference energies across different facets and substrates.

### Electrochemical Characterization

The electrochemical performances of asymmetric, symmetric, and full-cells were evaluated using a battery cycler (WonAtech Co. Ltd.) and VSP-3e potentio/galvanostat (Bio-logic Scientific Instruments) with CR2032-type coin cells. For symmetric cell testing, 100 μL of 2 M ZnSO_4_ aqueous electrolyte was used. For full cells using MnO_2_ cathodes, 120 μL of an aqueous electrolyte consisting of 2 M ZnSO_4_ and 0.1 M MnSO_4_ was used. To evaluate Coulombic efficiency (CE) during Zn plating/stripping, asymmetric cells were assembled using pristine SUS, rGO@SUS or AGI@SUS as the working electrode and Zn foil as both the counter and reference electrode. Chronoamperometry (CA) was performed at a constant potential of − 150 mV (vs. Zn^2+^/Zn). Initial nucleation potential and short-circuit time (T_SC_) tests were conducted at a constant current density of 1 mA cm^−2^. For full cell tests, galvanostatic charge/discharge and cyclic voltammetry (CV) measurements were taken within a voltage window of 0.8–1.8 V. Tafel polarization measurements were taken on symmetric cells within a potential range of ± 100 mV (vs. Zn^2+^/Zn), using 1 M Na_2_SO_4_ as the electrolyte**.**

## Results and Discussion

### Fabrication and Structural Characterization of BCP-Templated AGI

**Fig. 1:**
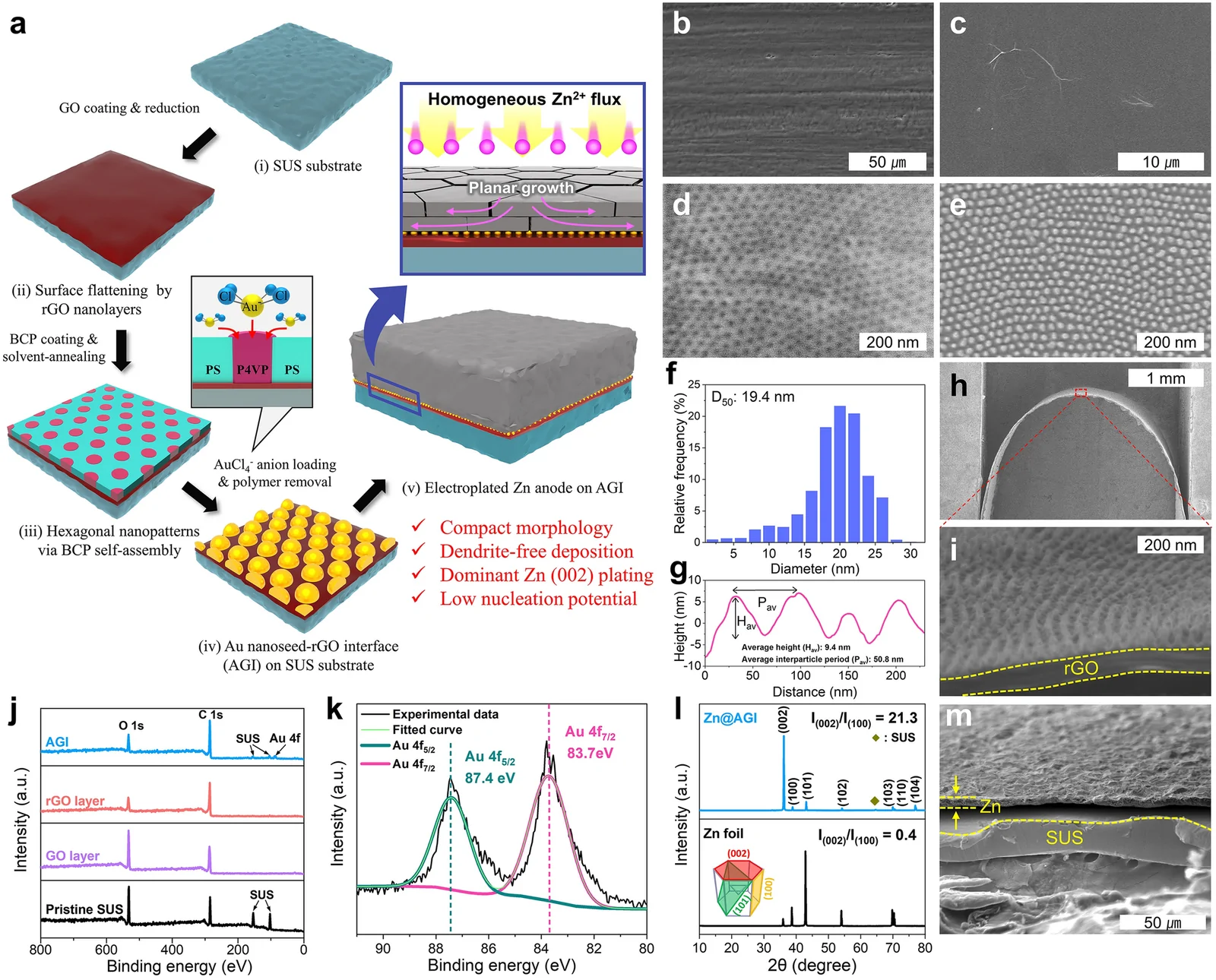
**a** Schematic illustration depicting the sequential fabrication steps of the Zn@AGI anode. SEM images of **b** pristine SUS, **c** rGO@SUS, **d** self-assembled BCP template formed on rGO@SUS and **e** the resulting AGI@SUS after BCP nanopattern transfer process. **f** Size distribution analysis of Au nanoseed arrays. **g** AFM profiles obtained from Fig. S5c, showing the average height and interparticle period of the Au nanoseed arrays. **h** Low-magnification SEM image of the AGI@SUS substrate in a curved form. **i** Magnified SEM image of **h**, clearly demonstrating the successful fabrication of the AGI@SUS substrate. **j** Wide-scan XPS spectra of SUS, GO, rGO and AGI. **k** Deconvoluted XPS core scans of Au 4*f* from AGI@SUS. **l** XRD patterns of the commercial Zn foil and the electroplated Zn anode on AGI@SUS. **m** Cross-sectional view of the plated Zn anode on AGI@SUS (~ 1.6 mAh cm^−2^)

In establishing the nanoscale interfacial architecture that stabilizes Zn anodes, self-assembled BCP templates were utilized as a nanopatterning tool to create well-ordered Au nanoseed patterns on the rGO nanolayer [38, 39]. BCP-templated nanopatterning provides a scalable and manufacturing-compatible approach for introducing ordered nanoscale metal seed architectures over large areas, and such patterns can be effectively utilized to regulate initial ion nucleation and growth of Zn anodes, offering particular advantages for AZIBs where early nucleation behavior critically influences overall performance [40, 41]. The term “Au nanoseed” refers to spatially confined Au nanoparticles that act as nucleation sites for homogeneous Zn plating. Figure 1a schematically describes the overall fabrication procedure. First, the rGO nanolayer was prepared on the SUS substrate by multiple spin coating steps using a GO dispersion, followed by thermal reduction. Then, an amphiphilic BCP, poly(styrene-*block*-4-vinylpyridine) (PS-*b*-P4VP, 25.0 kg mol^−1^ for the PS block and 10.0 kg mol^−1^ for the P4VP block), was spin-coated onto the rGO surface. Subsequently, the BCP thin film underwent solvent-vapor annealing using a toluene/THF vapor environment, which induced microphase separation of BCPs and resulted in the formation of vertically aligned P4VP nanocylinders embedded in the PS matrix. Immersion of the BCP thin film in a weakly acidic aqueous solution containing Au precursors (1 mM HAuCl_4_ in 0.01wt% HCl) enabled strong and site-specific electrostatic interactions of AuCl_4_^−^ anions with the protonated pyridine groups in the P4VP nanocylinders. Subsequent oxygen plasma treatment effectively removed the organic BCP matrix, and the following thermal treatment yielded the AGI on the SUS substrate, with monodisperse Au nanoseed arrays selectively positioned at the original locations of the P4VP nanocylinders. The corresponding substrate images and detailed preparation process for GO-coated SUS (GO@SUS), rGO-coated SUS (rGO@SUS) and AGI-coated SUS (AGI@SUS) are presented in Fig. S1. Finally, Zn electroplating on AGI produced a highly stable and uniform Zn anode, exhibiting a low nucleation overpotential (η), indicative of a reduced kinetic barrier for Zn nucleation rather than a thermodynamic shift, along with compact, dendrite-free plating and a predominant (002) orientation.

Figure 1b, c shows SEM images of the pristine SUS and rGO@SUS substrate, respectively. Spin coating was employed as a scalable and effective method for the uniform coating of the GO nanolayer on the SUS substrate (Fig. S2). Subsequent thermal reduction resulted in ~ 20-nm-thick rGO layer with a densely stacked structure (Figs. S3 and S4). After the formation of the rGO nanolayer, the intrinsically rough surface of the SUS substrate was substantially smoothed as evidenced by AFM surface roughness measurements (Fig. S5a, b). Figure 1d shows the surface morphology of the self-assembled BCP thin film formed on rGO@SUS. The P4VP nanocylinders were vertically aligned and arranged in a well-defined hexagonal pattern within the surrounding PS matrix, indicating successful microphase separation via solvent annealing (Figs. S6a, S6b). It is worth noting that the lateral ordering of the P4VP nanocylinders was strongly influenced by solvent annealing conditions, with 300 µL of solvent and 3 h of annealing identified as optimal in this study (Fig. S6c). In particular, the inherent surface roughness of the pristine SUS substrate was found to hinder the lateral ordering and uniform microphase separation of the BCP thin film, highlighting the necessity of the rGO nanolayer in surface planarization (Fig. S7). After the pattern transfer from the BCP template, Au nanoseed arrays with D_50_, average height and interparticle period of 19.4, 9.4 and 50.8 nm, respectively, were formed on rGO@SUS, resulting in AGI@SUS, as shown in Figs. 1e–g and S5c. Due to the slight vertical swelling of the acid-stimulated P4VP block during the AuCl_4_^−^ anion loading step, the resulting Au nanoseed arrays exhibited a larger lateral diameter than the original P4VP nanocylinders as illustrated in Fig. S8. An optimal loading time of AuCl_4_^−^ anions (30 s) was identified in this study as prolonged loading led to irregularly shaped nanoseeds and even interconnections between neighboring seeds (Fig. S9). The large-area SEM observation demonstrated the reliability of the BCP template-based pattern transfer process for scalable fabrication of Au nanoseed arrays (Fig. S10). Figure 1h presents a low-magnification SEM image of curved AGI@SUS for capturing a cross-sectional view of AGI. The successful fabrication of AGI@SUS was further confirmed by enlarged observations (Fig. 1i).

The surface chemistry of the prepared substrates was examined by X-ray photoelectron spectroscopy (XPS) analysis. Compared to GO@SUS, rGO@SUS showed enhanced reduction features, while AGI@SUS exhibited an additional Au peak, indicating the presence of metallic Au nanoseeds (Fig. 1j, k). Moreover, the C 1*s* scan of the rGO nanolayer in AGI@SUS displayed a deeper reduction state than that in rGO@SUS, presumably due to the catalytic influence of Au nanoseeds, which is unattainable through simply extending the thermal treatment in the case of rGO@SUS (Fig. S11) [42, 43]. During the polymer removal step, the oxygen plasma conditions were carefully optimized to avoid damaging the underlying rGO nanolayer, as excessive etching could compromise its structural integrity. Figure 1l compares X-ray diffraction (XRD) patterns of the commercial Zn foil and the electroplated Zn anode on AGI@SUS. The Zn foil exhibited typical diffraction features of a hexagonal close-packed (hcp) crystal structure, with dominant peaks corresponding to (002), (100) and (101) planes [36]. In contrast, the electroplated Zn anode on AGI@SUS showed a strong preferential orientation along the (002) facet, as evidenced by the pronounced intensity of the Zn (002) peak. In particular, the orientation factor (*R* = *I*_(002)_/*I*_(100)_) reaches 21.3, far exceeding that of the Zn foil (0.4), indicating dominant (002) growth. This preferential orientation is attributed to the synergistic effect of the AGI, which will be discussed in detail in the following section. Figure 1m shows the cross-sectional view of the plated Zn anode (~ 1.6 mAh cm^−2^), revealing a compact and uniform Zn layer.

### Characterization of Substrate-Guided Zn Anodes

**Fig. 2:**
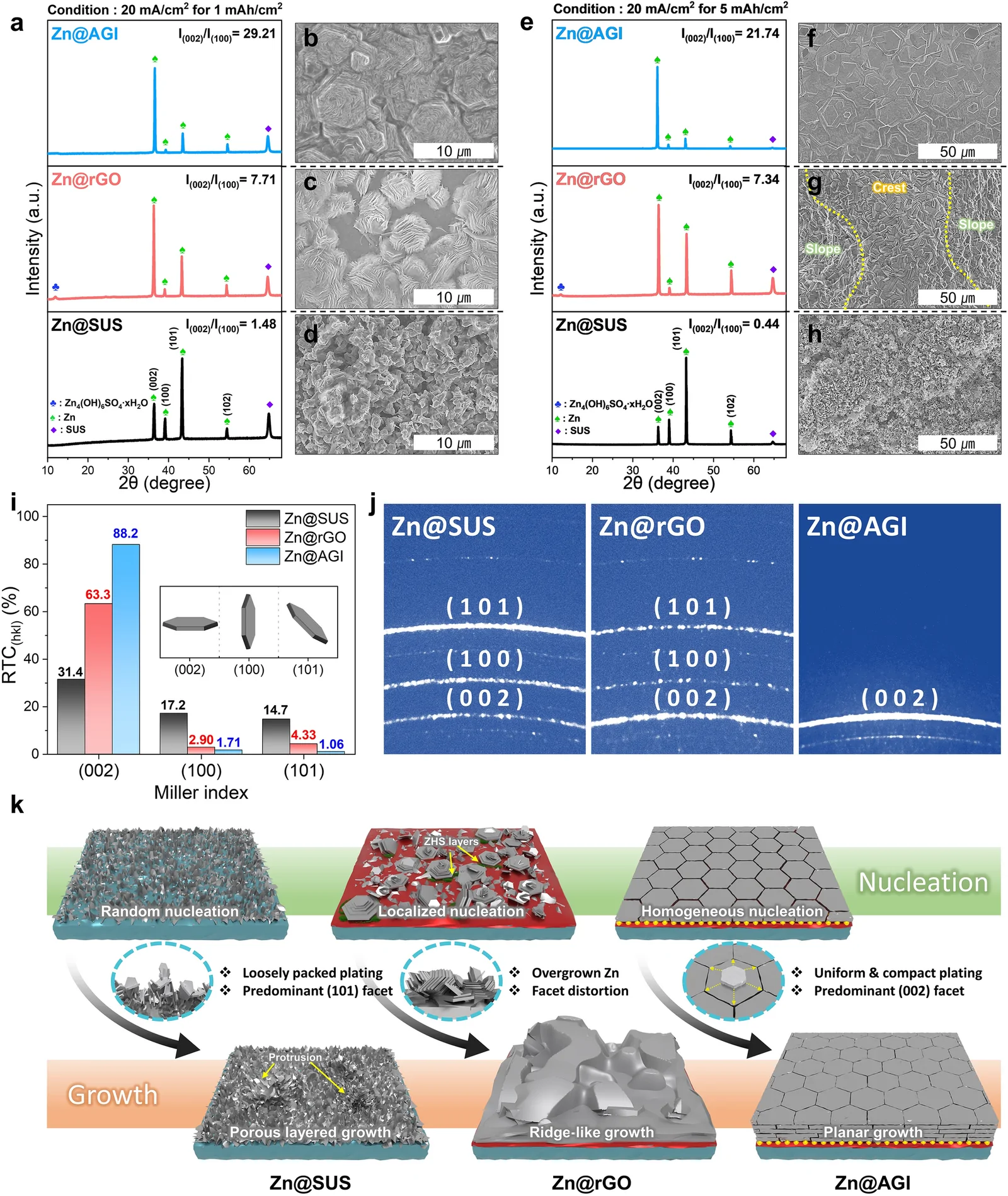
Comparative characterization of Zn plating behavior across different substrates to reveal substrate-dependent effects **a** Characteristic XRD patterns of the Zn anodes plated at a current density of 20 mA cm^−2^ to a capacity of 1 mAh cm^−2^. Corresponding SEM surface images of **b** Zn@AGI, **c** Zn@rGO and **d** Zn@SUS. **e** Characteristic XRD patterns of the Zn anodes plated at a current density of 20 mA/cm^2^ to a capacity of 5 mAh/cm^2^. Corresponding SEM surface images of **f** Zn@AGI, **g** Zn@rGO and **h** Zn@SUS. Individual value of the orientation factor (*R* = *I*_(002)_/*I*_(100)_) for each plated Zn anode was calculated from the XRD results and indicated within the corresponding XRD plot. **i** Relative texture coefficient (RTC) and **j** 2D-GIXRD patterns of three Zn anodes plated at 20 mA cm^−2^–5 mAh cm^−2^. **k** Schematic comparison of Zn plating behavior on different substrates, highlighting substrate-guided growth mechanisms

To elucidate the substrate dependency of Zn plating behavior, we systematically investigated Zn growth on various substrates under controlled electroplating conditions. Since the initial plating critically affects the subsequent growth characteristics, we primarily examined the crystal facet distribution and surface morphology of the Zn anodes at a capacity of 1 mAh cm^−2^ under a current density of 20 mA cm^−2^ (Fig. 2a–d). Owing to the low plating capacity of 1 mAh cm^−2^, which theoretically corresponds to a Zn thickness of ~ 1.7 μm, all Zn anodes exhibited detectable XRD peaks from the underlying SUS substrate. Notably, the SUS peak in Zn@SUS and Zn@rGO appeared slightly more intense than that in Zn@AGI, which is attributed to locally exposed regions of the SUS substrate that were not fully covered by plated Zn. While Zn@SUS exhibited a loosely packed plating morphology, Zn@rGO showed incomplete coverage in many areas as evidenced by SEM images. In contrast, a uniformly covered Zn layer was observed in Zn@AGI, indicating improved plating homogeneity. In terms of crystallographic texture, three Zn anodes exhibited distinctly different XRD patterns, reflecting the strong influence of the underlying substrate on the orientation of plated Zn. Regardless of the substrate type, *R* values were higher than that of the pristine Zn foil (0.4). This is consistent with previous findings, suggesting that increased cell overpotential at higher current densities, such as 20 mA cm^−2^, favors the development of (002) texture [44, 45]. However, Zn@SUS exhibited irregularly shaped Zn deposits with a persistent dominance of the (101) crystallographic facet. This can be attributed to the kinetically favorable growth and stronger Zn–Zn bonding of the (101) plane, especially in the absence of substrate-guided growth and sufficient zincophilicity [36, 46]. For Zn@rGO, a relatively high R value of 7.71 indicates enhanced (002) growth facilitated by the underlying rGO nanolayer, likely due to improved lattice compatibility with the Zn (002) facet. However, a comparable (101) peak was also observed, resulting in an unusual non-horizontal layered Zn growth. Among all samples, Zn@AGI exhibited the most intense (002) reflection, with the highest *R* value of 29.21, reflecting a strongly preferred (002) orientation and planar Zn plating.

To further investigate the effect of the substrate on Zn growth behavior, the plated Zn capacity was increased to 5 mAh cm^−2^ while maintaining the current density of 20 mA cm^−2^, and the resulting morphological and crystallographic changes were analyzed (Fig. 2e–h). As expected, the initial morphology of Zn@SUS at 1 mAh cm^−2^ significantly dictated the subsequent Zn plating, yielding more pronounced (101) and (100) peaks and a correspondingly reduced R value of 0.44. Zn@rGO and Zn@AGI also preserved their initial morphologies and crystallographic features. Despite the high R value of 7.34, the (101) facet was nonetheless apparent in Zn@rGO. For Zn@AGI, a uniformly planar, compact and dendrite-free morphology was observed, implying that interface-guided homogeneous Zn^2+^ flux and planar growth remained effective even at the higher plating capacity. Further insights into the synergistic interfacial effect of the Au nanoseed arrays and the rGO nanolayer will be provided in the mechanism discussion section. Interestingly, Zn@rGO still exhibited a noticeable SUS peak, whereas other two anodes showed negligible signals. This observation contradicts expectations based on the theoretical thickness (~ 8.5 μm) of the Zn layer corresponding to a plating amount of 5 mAh cm^−2^. In this regard, a ridge-like morphology of Zn@rGO, as shown in Fig. 2g, indicates localized overgrowth accompanied by substantial thickness variation on a macroscopic scale. Despite the rGO layer’s ability to guide Zn growth along the (002) facet via lattice compatibility, residual oxygen-containing defects (e.g., –OH, –COOH) in rGO exhibited strong Zn^2+^ affinity, leading to preferential plating at these sites and inducing distorted rather than planar (002) growth, which resulted in localized dendritic overgrowth of Zn. Moreover, Zn@rGO displayed a noticeable signal corresponding to zinc hydroxide sulfate (ZHS, Zn_4_(OH)_6_SO_4_·xH_2_O), which was not observed in the other anodes [47]. This may be associated with incompletely reduced GO regions, where oxygen defects induce local H_2_O and OH^−^ generation during Zn plating, thereby facilitating the formation of resistive ZHS layers as explained in Fig. S12a [48–50]. Notably, these ZHS layers were found near the surfaces where Zn plating initially occurred (Fig. S12b, c). In addition, after the intentional removal of the plated Zn, intense ZHS signals were detected, reconfirming that ZHS layers primarily formed on the rGO surface (Fig. S12d).

Since the applied current density is a critical factor influencing the characteristics of plated Zn, the samples were fabricated by varying the current density from 1 to 20 mA cm^−2^ under the fixed Zn capacity of 5 mAh cm^−2^ (Figs. S13–S15). In general, the R value increased with higher current density for all samples, reflecting enhanced (002) oriented Zn growth, except for Zn@SUS, as summarized in Fig. S16. Most importantly, the ZHS layers were detected at lower current densities (< 10 mA cm^−2^), likely due to prolonged exposure to water and lowered overpotential for hydrogen evolution reaction (HER), consistent with previous reports [32, 33]. This behavior is also presumably related to the greater reactivity of the (100) and (101) facets toward water [45]. Thus, a higher current density of 20 mA cm^−2^ is considered a suitable condition for achieving uniform, horizontally oriented Zn plating while avoiding ZHS formation. However, further increasing the current density to 40 mA cm^−2^ resulted in a distorted plating morphology, likely due to the transition to a mass-transport-limited, non-equilibrium growth regime associated with local Zn^2+^ depletion near the substrate during rapid consumption (Fig. S17) [51]. These observations indicate that the enhanced (002) texture results from the combined effects of elevated current density and the presence of zincophilic Au nanoseed arrays, with Zn@AGI exhibiting more uniform and planar Zn plating than the control electrodes under the same electrochemical conditions. Zn texture evolution across three anodes (5 mAh cm^−2^ at 20 mA cm^−2^) was quantitatively compared by calculating the relative texture coefficient (RTC), as defined by Eq. (1) [44, 52]:

1 $${\mathrm{RTC}}_{{\left( {{\mathrm{hkl}}} \right)}} = \frac{{I_{{\left( {{\mathrm{hkl}}} \right)}} /I_{{0\left( {{\mathrm{hkl}}} \right)}} }}{{\sum I_{{\left( {{\mathrm{hkl}}} \right)}} /I_{{0\left( {{\mathrm{hkl}}} \right)}} }} \times 100$$

where *I*_(hkl)_ and *I*_*0*(hkl)_ represent the XRD peak intensities of the plated Zn anode and the standard Zn foil, respectively. In general, a higher RTC value indicates a more pronounced crystallographic texture. As shown in Fig. 2i, Zn@AGI exhibited the highest (002) RTC of 88.2%, significantly higher than those of Zn@rGO (63.3%) and pristine SUS (31.4%), and was accompanied by a corresponding decrease in (100) and (101) RTC. This analysis demonstrates that the introduction of the nanoscale AGI structure significantly enhances Zn (002) texturing. The crystallographic characteristics of the plated Zn anodes were further analyzed using grazing incidence X-ray diffraction (GIXRD) (Fig. 2j). Consistent with the XRD results, the Debye ring corresponding to the (002) facet became progressively more intense in the order of Zn@SUS, Zn@rGO and Zn@AGI. Figure 2k schematically illustrates the substrate-dependent Zn plating behavior. In the case of Zn@SUS, the absence of guiding features induces random nucleation and dendritic growth, resulting in a loose and porous Zn layer with localized protrusions. For Zn@rGO, the rGO nanolayer offers partial control over Zn orientation due to its moderate zincophilicity and lattice compatibility. However, incomplete reduction and local inhomogeneity result in the ridge-like morphology accompanied by ZHS formation, thereby disrupting uniform growth. In contrast, Zn@AGI exhibits a compact and dendrite-free structure. This is enabled by the synergistic interfacial architecture of Au nanoseed arrays on rGO, which equalizes Zn^2+^ ion flux and directs lateral Zn plating, thereby markedly strengthening (002) texture.

**Fig. 3:**
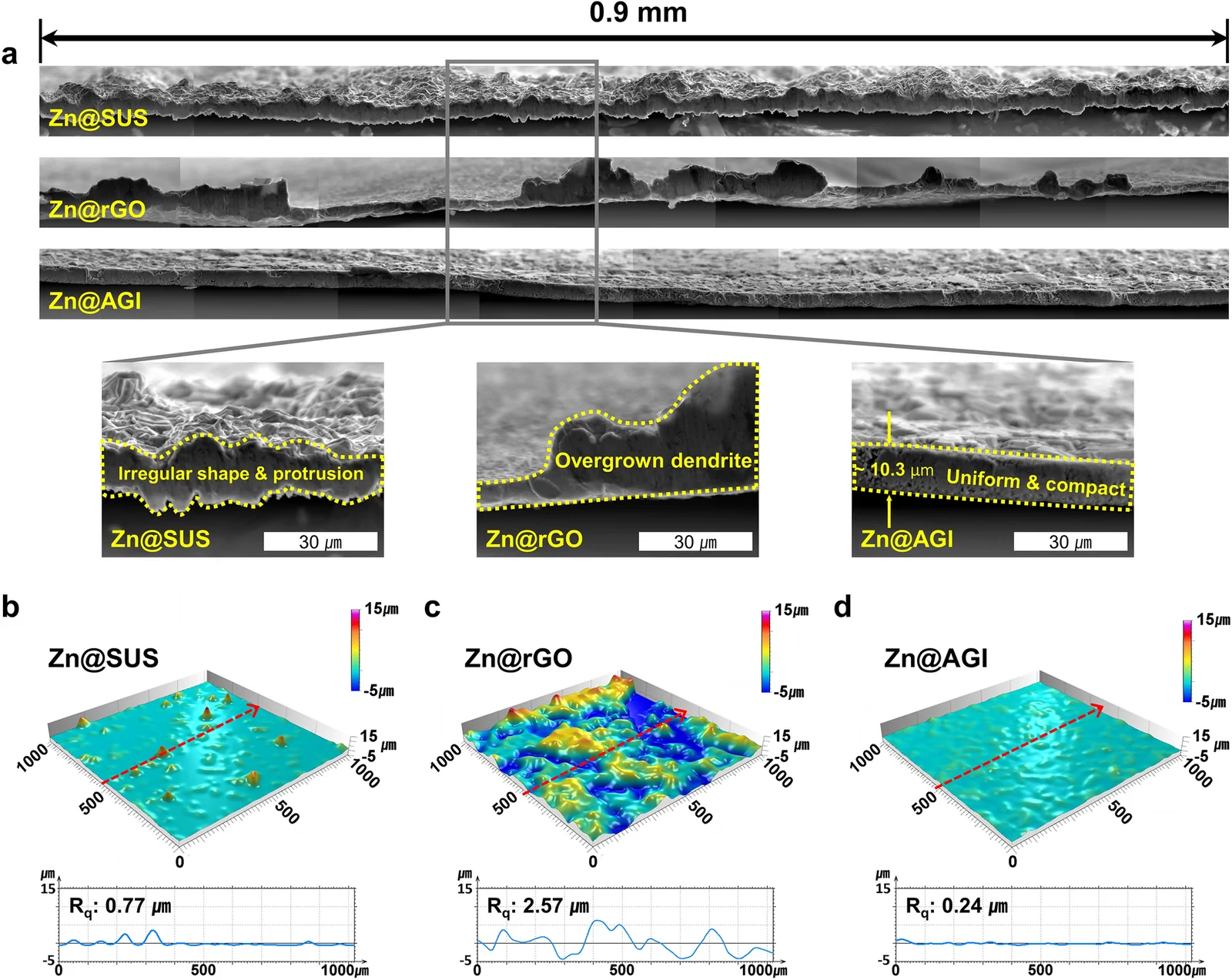
**a** Cross-sectional SEM images over ~ 0.9 mm range for Zn@SUS, Zn@rGO and Zn@AGI (5 mAh cm^−2^ at 20 mA cm^−2^), comparing substrate-dependent plating uniformity on a macroscopic scale. The magnified regions highlighted by gray boxes reveal distinct Zn plating morphologies that vary depending on the substrate. Confocal laser scanning microscope (CLSM) images and corresponding *R*_*q*_ values of **b** Zn@SUS, **c** Zn@rGO and **d** Zn@AGI

To compare the macroscopic uniformity of Zn plating across different substrates, large-area cross-sectional SEM images of the Zn anodes (5 mAh cm^−2^ at 20 mA cm^−2^), spanning ~ 0.9 mm, were analyzed, as shown in Fig. 3a. Zn@SUS exhibited irregularly shaped and non-uniform Zn layer without preferential growth orientation. In spite of relatively low roughness (*R*_*q*_ = 0.77 μm) measured by confocal laser scanning microscopy (CLSM), sporadically distributed protrusions were observed on Zn@SUS, indicating spatially uncontrolled Zn plating behavior (Fig. 3b). In the case of Zn@rGO, well-plated Zn was observed in localized regions. However, macroscopic observation revealed the emergence of abnormally overgrown dendrites accompanied by the highest roughness (*R*_*q*_ = 2.57 μm), suggesting non-uniform overall plating (Fig. 3c). Such macroscopic non-uniformity aligns with the features captured in the optical microscopy image of Zn@rGO (Fig. S18). In sharp contrast, Zn@AGI (*R*_*q*_ = 0.24 μm) exhibited the smoothest surface among all samples, with uniformly plated Zn and no signs of dendritic growth, which could improve intrinsically poor roughness of commercial Zn foil (Figs. 3d and S19). In addition, the measured thickness of ~ 10.3 μm further suggests the compact and dense Zn plating layer. Figure S20 shows cross-sectional SEM images of the Zn anodes plated at 5 mA cm^−2^–5 mAh cm^−2^ on the different substrates. At this slower rate, (002) orientation was reduced and ZHS formation became more noticeable. However, Zn@AGI sustained uniform Zn plating. In this sense, the rGO nanolayer, Au nanoseeds and the BCP-templated periodic structure play different but complementary roles in controlling Zn plating. The rGO nanolayer mainly smooths the substrate surface and facilitates ordered BCP assembly, while it may partially favor Zn growth along the (002) direction. By contrast, Zn@AGI sustains uniform Zn plating because the Au nanoseed arrays guide the Zn^2+^ flux and, together with their high zincophilicity, promote (002)-oriented nucleation and growth. Meanwhile, the BCP-templated nanopatterns regulate the spatial distribution of Au nanoseeds and further homogenize the local Zn^2+^ flux. In contrast to previously reported crystallographically predefined substrates [53, 54] or current-driven strategies [55–57], the AGI@SUS architecture adopts a more generally applicable interfacial design that enables robust and preferential Zn (002) plating on a commercially relevant current collector through rational interfacial engineering, thereby offering a practical and scalable route for Zn metal anodes without requiring single-crystal-like Zn templates or ultrahigh current densities.

### Theoretical Calculation on Zn Adsorption and Growth Mechanism

**Fig. 4:**
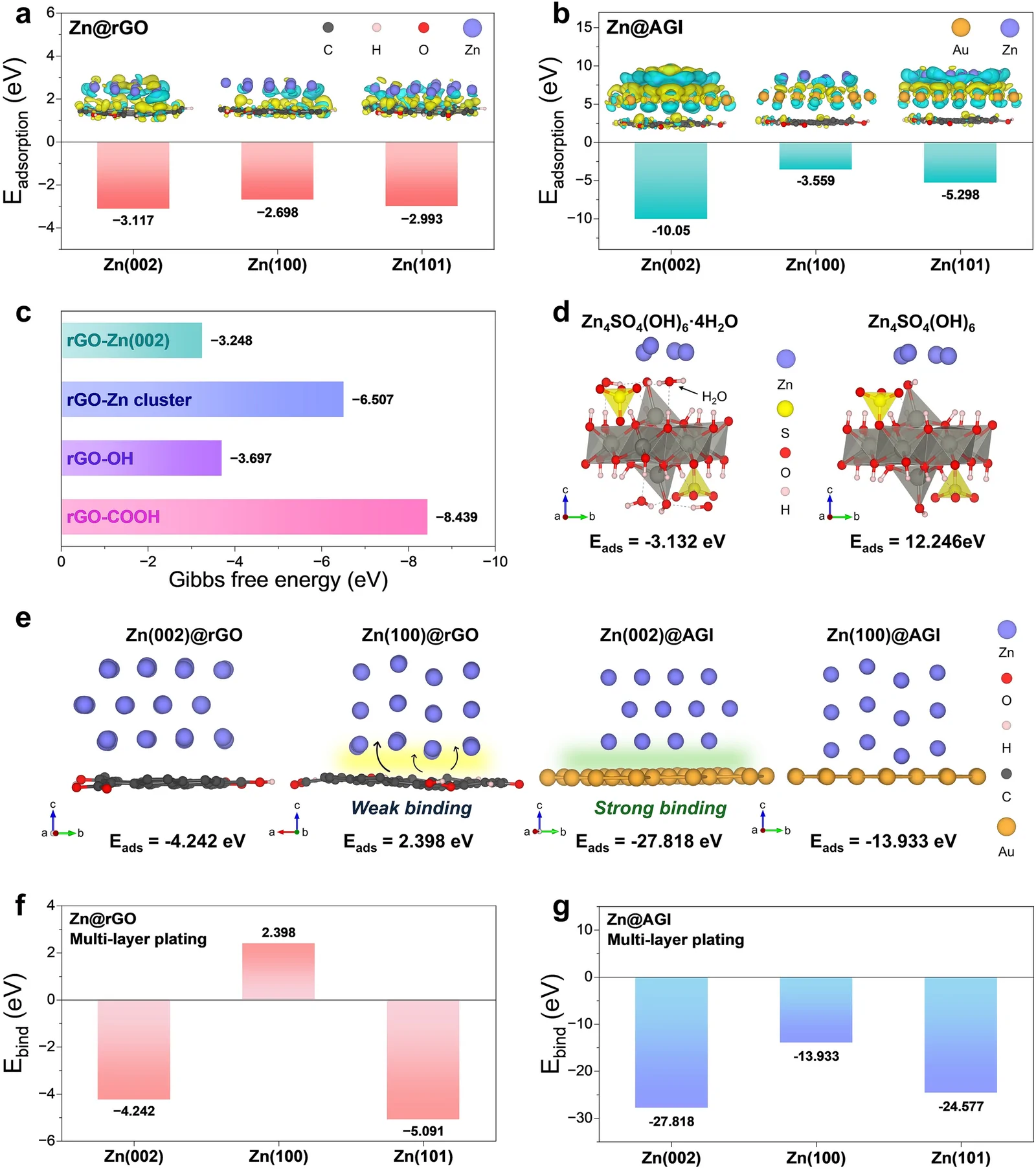
Calculated adsorption energies and charge density difference (CDD) plots for Zn slabs with Zn (002), Zn (100) and Zn (101) orientations on **a** the rGO substrate and **b** the AGI substrate; Yellow and cyan indicate charge accumulation and depletion, respectively. **c** Gibbs free energy plot comparing the thermodynamic favorability of Zn adsorption at different sites on rGO: Zn (002) facet, Zn cluster, hydroxyl-functionalized region (rGO–OH) and carboxyl-functionalized region (rGO–COOH). **d** Adsorption energies of Zn on hydrated (*x* > 0) and anhydrous (*x* = 0) ZHS (Zn_4_(OH)_6_SO_4_·xH_2_O) surfaces. **e** Optimized geometries of multi-layer Zn (002) and Zn (100) slabs on the rGO and AGI substrates, with the corresponding interfacial binding energies for different Zn orientations (multi-layer denotes a three-layer Zn slab). Calculated interfacial binding energies of multi-layer Zn slabs with (002), (100) and (101) orientations on **f** rGO and **g** AGI

To theoretically verify whether the presence of Au nanoseed arrays facilitates Zn (002) plating with a lower η, indicative of a reduced kinetic barrier for initial Zn nucleation, we conducted first-principles calculations comparing the adsorption energies of Zn on rGO and AGI substrates across various crystal facets: Zn (002), Zn (100) and Zn (101) (Figs. S21–S22) [30, 58]. As shown in Fig. 4a, Zn (002) on Zn@rGO exhibited the lowest adsorption energy of − 3.117 eV, followed by Zn (101) and Zn (100) with values of − 2.993 and − 2.698 eV, respectively. The relatively small difference between Zn (002) and Zn (101) indicates a weakly preferred orientation on rGO, consistent with the XRD peak comparison shown in Fig. 2e. In contrast, Zn@AGI exhibited a significantly lower adsorption energy of − 10.050 eV for Zn (002), indicating significantly enhanced Zn affinity on AGI (Fig. 4b) [59]. The stabilization of Zn (002) on AGI reflects the intrinsic zincophilicity of the Au-modified interface, which effectively lowers the heterogeneous nucleation barrier at the early plating stage. This strong interfacial interaction lowers the kinetic barrier for Zn nucleus formation at the Au-modified interface. As shown in the insets of Fig. 4a, b, the charge density difference (CDD) plots further support these findings: Zn@rGO exhibits localized and non-uniform electron accumulation/depletion due to residual oxygen-containing defects such as −COOH and −OH, whereas Zn@AGI shows a more continuous and homogeneous interfacial charge redistribution, resulting in a flattened surface potential and more stable Zn bonding [60, 61]. Notably, Zn (002) on AGI forms the most continuous layer-like interfacial charge redistribution. Such uniform charge redistribution on AGI can be attributed to Au nanoseeds, which promote a homogeneous interfacial electron distribution and stabilize Zn adsorption during early nucleation. In good agreement with the crystallographic and morphological results shown in Figs. 2 and 3, these findings demonstrate that the preferential Zn (002) adsorption on Zn@AGI originates from its uniform surface potential and strengthened Zn–AGI interfacial interaction, resulting in highly uniform Zn plating. To examine whether Zn–Au alloying contributes to the enhanced zincophilicity, we evaluated substitutional Zn incorporation into the Au nanoseed via formation energy analysis (Fig. S23) [62]. The positive substitution formation energy suggests that alloying is thermodynamically unfavorable under our modeled conditions. Therefore, the enhanced zincophilicity of AGI is primarily governed by adsorption-driven interfacial energetics rather than alloy-driven stabilization. Additionally, the lower adsorption energy of the Zn (101) on rGO in Fig. 4a suggests an association with residual oxygen-containing defects on the rGO surface. To elucidate this theoretically, the Gibbs free energies of Zn adsorption were compared for Zn (002) plating, Zn cluster formation, hydroxyl (−OH) regions and carboxyl (−COOH) regions on rGO.

According to the results in Figs. 4c and S24–S25, the reduction of −COOH to −CH_2_OH showed the lowest Gibbs free energy (− 8.439 eV), followed by Zn cluster formation (− 6.507 eV), reduction of − OH to − H (− 3.697 eV) and Zn (002) plating (− 3.248 eV). This indicates that during charging, electrons are primarily consumed in reducing oxygen-containing surface defects, which theoretically facilitates Zn nucleation directly at these defect sites [60, 61]. Subsequently, Zn atoms are predicted to aggregate into stable clusters around these reduced regions. In contrast, direct alignment and growth of Zn (002) layers require higher adsorption energy, making them less favorable on rGO. This aligns with the CDD plot of Zn (002)@rGO (Fig. 4a), which shows localized, non-uniform charge redistribution attributable to oxygen-containing defects (− COOH, − OH). Consequently, the reduction of Zn^2+^ ions during charging is delayed by the preferential electron consumption at such defects, which allows gradual accumulation of H_2_O and OH⁻ around Zn^2+^ ions, thereby favoring the initial nucleation of hydrated ZHS [48, 63]. Therefore, as shown in Figs. 2e and S12, the correlation between ZHS structural evolution and Zn plating behavior on rGO was theoretically elucidated by comparing Zn adsorption energies on hydrated and anhydrous ZHS surfaces. Zn showed stable adsorption on hydrated ZHS (− 3.132 eV), whereas Zn adsorption on anhydrous ZHS (+ 12.246 eV) was associated with unfavorable thermodynamics. This can be attributed to the polar coordination of –OH and H_2_O in hydrated ZHS, which facilitates Zn adsorption via covalent interactions, whereas the deficient coordination environment in anhydrous ZHS hinders such adsorption [64]. This theoretical interpretation is supported by the experimental observations in Fig. S12, where under low-current–density conditions, the slower Zn plating rate prolongs electrolyte/H_2_O contact with the rGO surface, thereby facilitating the nucleation of hydrated ZHS near defect sites (–COOH, –OH) and subsequently promoting Zn growth. In contrast, at high current densities, the accelerated Zn plating shortens the exposure time to the electrolyte and H_2_O, thereby suppressing ZHS hydration and significantly reducing ZHS formation. Under these conditions, Zn plating predominantly occurs directly on rGO rather than through a hydrated ZHS-mediated pathway.

To further examine orientation-dependent Zn growth, we compared the interfacial binding energies of multi-layer Zn slabs exposing Zn (002), Zn (100) and Zn (101) on rGO and the AGI substrates. As shown in Figs. 4e–f and S26 for multi-layer Zn slabs on rGO, the interfacial binding energy for Zn (100) is positive, indicating energetically unfavorable interfacial binding. Moreover, the Zn (101) on rGO is even lower than that of Zn (002), suggesting that growth along (101) is increasingly favored thermodynamically during continued plating. This explains the emergence of non-uniform surface orientations, including Zn (101), observed experimentally in Fig. 2c, g. On the other hand, as shown in Fig. 4g, AGI exhibits low interfacial binding energies across all examined facets, particularly enabling stable Zn plating with a dominant (002) orientation. To facilitate comparison of the absolute adhesion strength, we additionally report the work of adhesion per unit area, *W*_ad_ = [*E*_(substrate)_ + *E*_(multi-layer Zn slabs)_* − E*_(multi-layer Zn slabs + substrate)_]/*A*, where *A* is the interfacial area of the supercell, which yields consistent facet-dependent trends (Table S1). Notably, AGI shows the largest *W*_ad_ for the Zn (002) slab among the examined facets, indicating the strongest interfacial adhesion for the (002) oriented overlayer and providing a thermodynamic basis for the dominant Zn (002) plating on AGI. This behavior is characterized by a lower *η* on AGI, which enhances Zn affinity and promotes uniform and planar Zn plating. Accordingly, the multi-layer Zn adsorption energy results on AGI theoretically rationalize the uniform Zn (002) plating observed on AGI surfaces in Fig. 2f. A control interface based on non-zincophilic Fe_2_O_3_ nanoseeds on rGO (Zn@FGI) exhibited a lower R value of 3.08 together with distorted morphologies, indicating that Fe_2_O_3_ nanoseeds are ineffective in directing Zn (002) growth (Fig. S27).

### Electrochemical Performance Evaluation

**Fig. 5:**
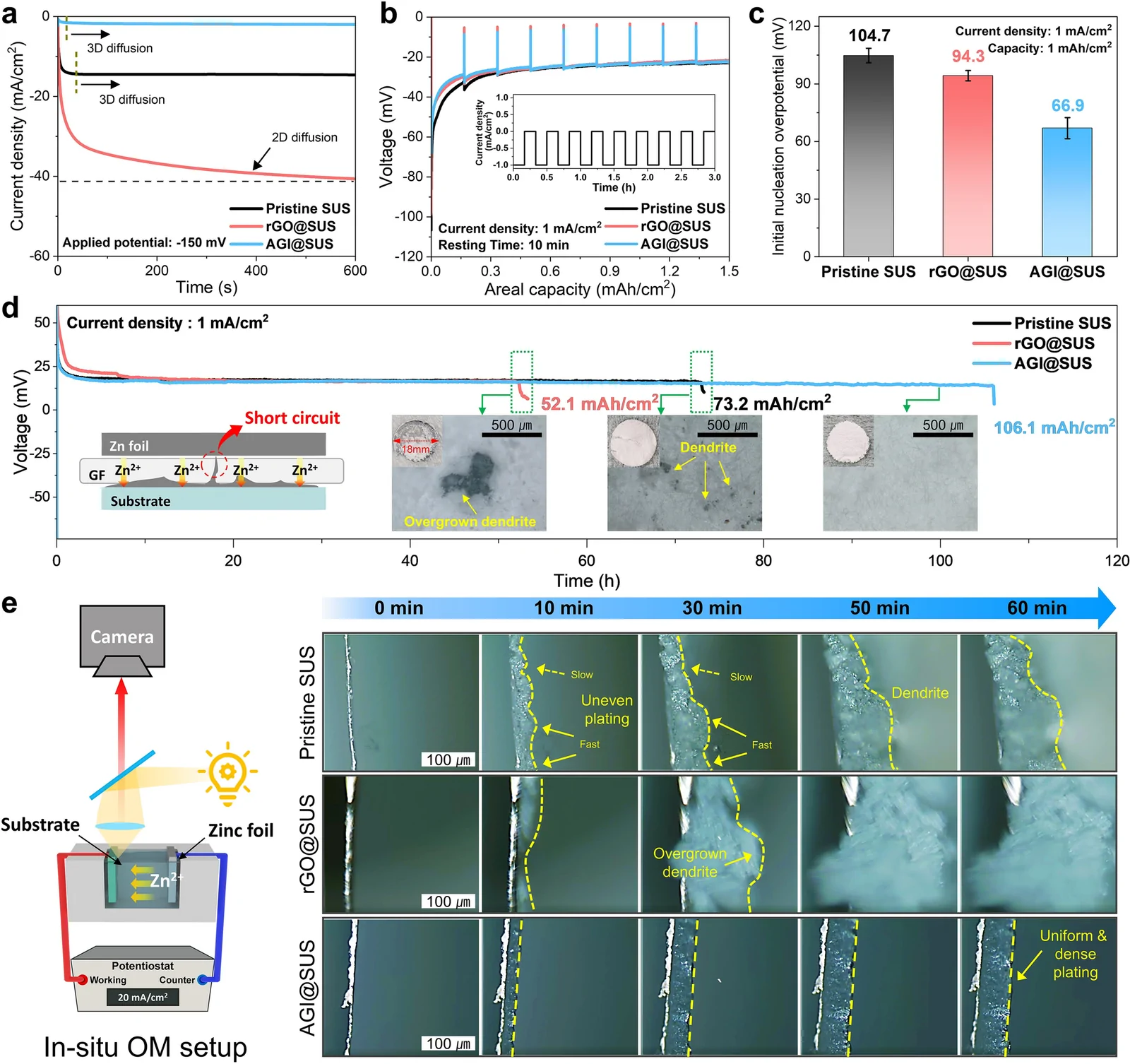
**a** Comparison of chronoamperometry (CA) profiles during Zn plating on pristine SUS, rGO@SUS and AGI@SUS substrates, highlighting differences in initial current response and plating behavior. **b** Voltage–capacity profiles recorded during intermittent electrodeposition at 1 mA/cm^2^ with 10 min rest intervals. The inset in **b** displays the corresponding cycling protocol. **c** Comparison of initial *η* for Zn plating on different substrates. **d** Short-circuit time (*T*_sc_) test conducted in an asymmetric cell configuration using pristine SUS, rGO@SUS and AGI@SUS as plating substrates. The inset shows optical microscopy (OM) images of the glass fiber separator surfaces after *T*_sc_ for pristine SUS and rGO@SUS, and during ongoing plating for AGI@SUS. **e** Schematic of the in situ OM setup and real-time observation of Zn plating behavior on three substrates at 20 mA/cm^2^ over 60 min

To assess the substrate-dependent Zn plating stability, particularly nucleation and ion diffusion characteristics, chronoamperometry (CA) technique was employed (Fig. 5a). It should be noted that in this study, the Zn@AGI configuration was adopted because a direct comparison of substrate-supported plated Zn and peeled-off Zn films at the same areal capacity (5 mAh cm^−2^, ~ 10 μm) revealed severe cracking and tearing in the peeled Zn layers (Fig. S28). A constant potential of − 150 mV was applied to the half-cell configuration using the substrates as the working electrodes. It is well known that during the initial stage of plating governed by 2D diffusion, uneven ion flux leads to a continuous increase in the metal surface area. A pronounced rise in surface area reflects accelerated rough Zn growth and the onset of localized dendritic structures [65]. In this regard, the current density of the cell with rGO@SUS kept increasing over 600 s, suggesting an uncontrolled 2D diffusion-dominated plating process. This prolonged plating likely contributed to the formation of macroscopic non-uniformities, such as overgrown dendrites observed in Fig. 3a. For pristine SUS, nucleation and 2D ion diffusion occur within 36 s; however, initial dendritic growth and random orientation may disrupt the uniformity of subsequent Zn plating. In contrast, AGI@SUS achieved current stabilization rapidly, indicating that uniform 3D diffusion was promoted on its surface. As a result, Zn plating on AGI@SUS was horizontal and compact, in agreement with SEM and CLSM observations. Notably, the cell with AGI@SUS exhibits a much lower current density, suggesting limited increase in specific surface area [66]. Figure 5b compares voltage–capacity profiles during the intermittent electrodeposition of Zn at the constant current of 1 mA cm^−2^ and rest time of 10 min. Compared to pristine SUS and rGO@SUS, AGI@SUS attained equilibrium potential rapidly, indicating that Zn plating process is more growth-controlled than nucleation-controlled [4, 67]. This implies rapid and uniform nucleation on AGI@SUS, facilitated by its high zincophilicity and homogeneous Zn^2+^ ion flux. The plating then proceeded in a stable lateral manner without dendritic growth. Measuring the initial *η* further corroborated these findings, with AGI@SUS exhibiting the lowest value of 66.9 mV (Figs. 5c and S29), consistent with facilitated and spatially uniform Zn nucleation on the AGI surface.

To investigate the substrate-dependent plating capacity limit leading to cell failure via internal short-circuiting, a short-circuit time (*T*_sc_) test was conducted (Fig. 5d). The test was performed in an asymmetric cell under a constant current density of 1 mA cm^−2^. rGO@SUS exhibited the shortest lifespan, failing after 52.1 h (≈52.1 mAh cm^−2^), as local defect regions in rGO likely triggered localized overgrowth of dendritic Zn. This abnormal dendrite formation was clearly confirmed by postmortem analysis following short-circuiting (inset images in Fig. 5d), which revealed gigantic Zn dendrites penetrating into the separator. In comparison, pristine SUS endured longer plating time of 72.9 h (≈ 72.9 mAh cm^−2^), however, eventually failed due to the gradual accumulation of sharp Zn dendrites. In stark contrast, AGI@SUS showed the longest *T*_sc_ of up to 106.1 h (≈106.1 mAh cm^−2^) attributable to the synergistic interplay between Au nanoseed arrays and the underlying rGO nanolayer, which collectively promoted the formation of dense, well-oriented Zn deposits. SEM observations on the glass fiber separator side after the *T*_sc_ test further corroborated this behavior, as Zn protrusions penetrating the separator were clearly observed for pristine SUS and rGO@SUS, whereas no Zn was detected in the case of AGI@SUS even after 100 mAh cm^−2^ of plating (Fig. S30). To visualize the Zn plating dynamics in real time, in situ optical microscopy (OM) was employed, as illustrated in Fig. 5e alongside the schematic of the observation setup. Zn plating was conducted under a current density of 20 mA cm^−2^ for 60 min. Owing to poor Zn^2+^ affinity and lack of nucleation-site control, SUS exhibited immediate dendrite initiation, followed by tip-effect-driven growth. Meanwhile, rGO@SUS showed highly unstable growth behavior, with overgrown dendrites progressively emerging upon nucleation and expanding in an unregulated manner. In contrast, AGI@SUS demonstrated laterally uniform Zn growth from the onset, which remained consistent throughout plating up to 20 mAh cm^−2^.

**Fig. 6:**
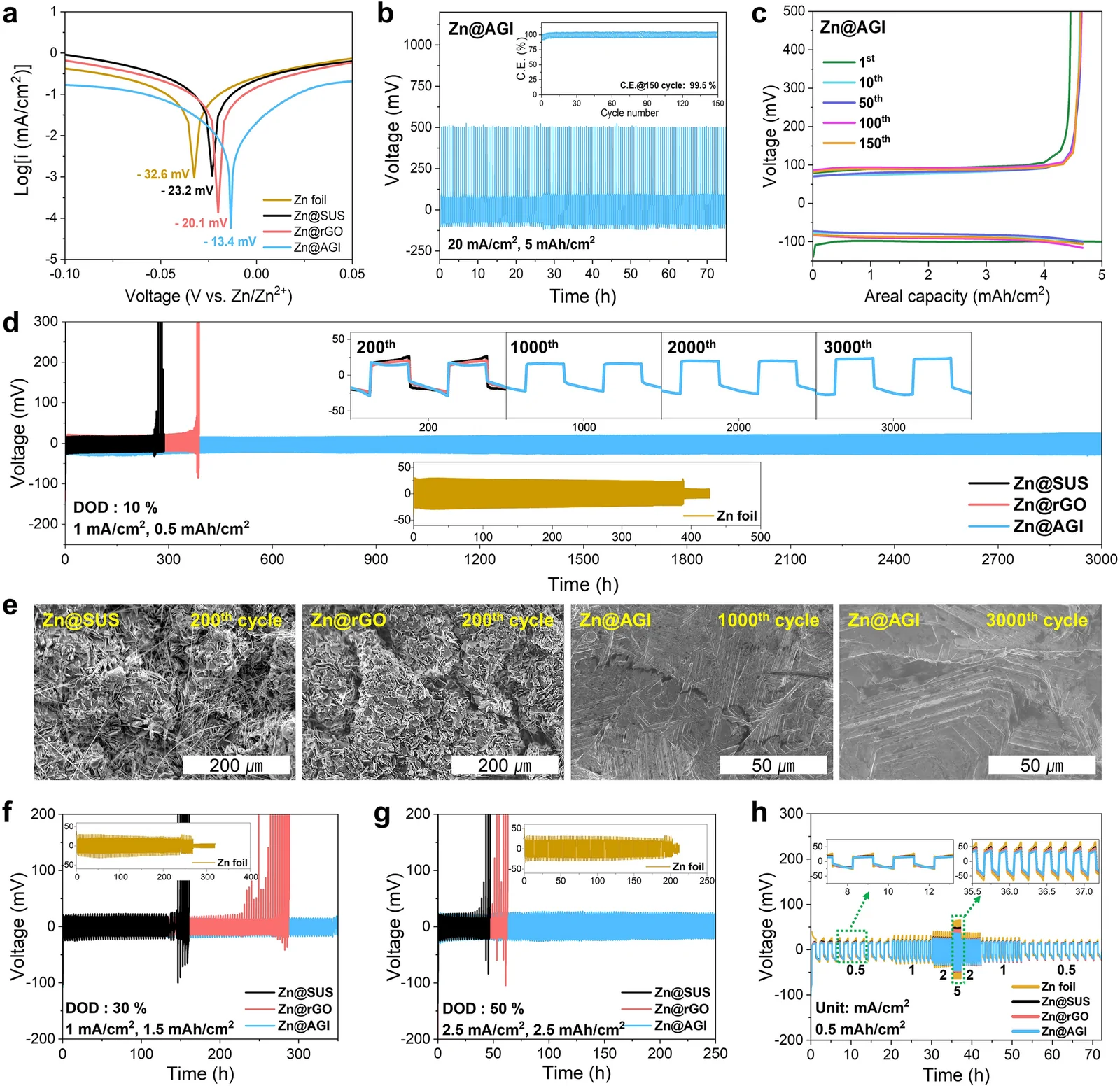
**a** Tafel polarization curves of the different Zn anodes measured in 1 M Na_2_SO_4_ electrolyte, showing corrosive currents (*I*_corr_) and potential (*P*_corr_). **b** Voltage–time profiles of the asymmetric cell with Zn@AGI cycled at 20 mA cm^−2^ for a plating capacity of 5 mAh cm^−2^. The inset displays Coulombic efficiency over cycling. **c** Selected voltage–capacity curves corresponding to (b). **d** Long-term cycling performance of symmetric cells using Zn@SUS, Zn@rGO and Zn@AGI cycled at 1 mA cm^−2^ with a plating/stripping capacity of 0.5 mAh cm^−2^ (corresponding to 10% DOD). Top inset shows selected voltage profiles over cycling. Bottom inset shows the cycling performance for Zn foil anode. **e** SEM images of cycled anodes: Zn@SUS and Zn@rGO after 200 cycles, and Zn@AGI after 1000 and 3000 cycles. Cycling performance of symmetric cells at higher DOD of **f** 30% (1 mA cm^−2^, 1.5 mAh cm^−2^) and **g** 50% (2.5 mA cm^−2^, 2.5 mAh cm^−2^). The result for Zn foil is provided in the inset for comparison. **h** Rate capability comparison of symmetric cells under various current densities (0.5, 1, 2, 5 mA cm^−2^) at a fixed plating/stripping capacity of 0.5 mAh cm^−2^

Tafel polarization measurements were taken to assess corrosive behavior such as corrosion current (*I*_corr_) and potential (*P*_corr_) for the Zn anodes (Fig. 6a). Both *I*_corr_ and *P*_corr_ exhibited a decreasing trend in the order of Zn@SUS > Zn@rGO > Zn@AGI. Zn@AGI has the lowest *I*_corr_ (0.053 mA cm^−2^) and P_corr_ (− 13.4 mV) among all samples, indicating the highest corrosion resistance and revealing a clear correlation between Zn (002) texture and electrochemical stability. The measured *I*_corr_ and *P*_corr_ values for all samples, including bare Zn foil, are summarized in Table S2. In addition, XRD patterns of Zn anodes as a function of immersion time in 2 M ZnSO_4_ solution revealed distinct differences in by-product formation (Fig. S31). While Zn@SUS and Zn@rGO showed clear peaks corresponding to ZHS, indicating chemical instability, Zn@AGI exhibited no detectable by-products, confirming its excellent resistance to electrolyte-induced degradation. Moreover, linear sweep voltammetry (LSV) measurements revealed that Zn@AGI exhibits the most negative HER potential among the tested electrodes, demonstrating that AGI effectively suppresses hydrogen evolution and water-driven parasitic reactions at the Zn surface (Fig. S32). To further evaluate the reliable plating/stripping behavior of the Zn anodes, which is critically influenced by plating morphology and corrosion resistance, asymmetric cells were assembled and cycled at a current density of 20 mA cm^−2^ with a plating/stripping capacity of 5 mAh cm^−2^. While Zn@SUS exhibited a gradual increase in overpotential before failing at around 35 h, Zn@rGO failed abruptly after only four cycles (Fig. S33). In contrast, Zn@AGI maintained stable cycling for over 75 h corresponding to 150 cycles, exhibiting an outstanding Coulombic efficiency of 99.5% (Fig. 6b). XPS shows that the Au 4f_7/2_ peak remains unchanged after Zn plating/stripping, indicating preserved metallic Au without detectable alloying, while the Zn 3*p* signal appearing after stripping, suggests zincophilic adsorption at the Au nanoseeds (Fig. S34). The voltage–capacity curves revealed negligible variation in overpotential throughout the cycling test, reflecting the superior cycling reversibility of Zn@AGI, as shown in Fig. 6c.

In practical AZIBs, the extent of Zn utilization quantified by DOD is a key factor influencing the achievable energy density at the device level [68]. In particular, realizing high DOD is quite challenging for thin Zn anodes as they are more subject to structural degradation during repeated cycling. In this regard, symmetric cell tests were conducted to evaluate the long-term cycling performance of the plated thin Zn anodes at varying DOD levels. Symmetric cells were first tested at 1 mA cm^−2^ with a capacity of 0.5 mAh cm^−2^ corresponding to 10% DOD as shown in Fig. 6d. Impressively, Zn@AGI showed exceptional cycling stability for more than 3000 h, far surpassing those of Zn@SUS (223 h) and Zn@rGO (409 h). To clarify whether this superior stability originates from intrinsic anode reversibility, capacity-limited reversibility tests were further conducted. These tests show a higher initial Coulombic efficiency for Zn@AGI (~ 93.0%) than for Zn@SUS and Zn@rGO, indicating intrinsically improved Zn reversibility (Fig. S35). Moreover, the selected voltage–time profiles at the 200, 1000, 2000 and 3000 h, as shown in the top inset of Fig. 6d, retained a near-ideal rectangular shape with only a marginal overpotential increase, indicating highly stable plating/stripping behavior over prolonged cycling. It is particularly intriguing that the cell failures accompanied by a sudden rise in overpotential in Zn@SUS and Zn@rGO differ from typical short-circuiting, suggesting a distinct and critical degradation pathway [44, 69]. Given the lower reversible Zn capacity, this abnormal rise in overpotential can be attributed to surface degradation and the formation of a porous structure of plated/stripped Zn, which ultimately hinders Zn^2+^ diffusion at the electrode–electrolyte interface, consistent with previous reports [69]. For a 250-μm-thick Zn foil, a capacity of 0.5 mAh cm^−2^ corresponds to a DOD of only 0.34%. Nevertheless, the cell failed due to short-circuiting after 388 h, in contrast to the failure mode observed in the plated Zn anodes, as shown in the bottom inset of Fig. 6d. This clearly distinguishable cycling stability is further supported by postmortem SEM images (Fig. 6e). The surface images obtained after 200 cycles revealed that a porous plated Zn surface locally entangled with the glass fiber separator in Zn@SUS and relatively large Zn deposits segmented by visible cracks in Zn@rGO. In contrast, Zn@AGI exhibited a flat and uniform plated Zn layer with negligible signs of porosity, even after extended cycling for 1000 and 3000 h. Figure 6f, g presents the cycling performance of Zn anodes under the more demanding DOD levels of 30% and 50%, respectively. Remarkably, Zn@AGI continued to deliver outstanding stability with minimal overpotential fluctuations, whereas Zn@SUS and Zn@rGO exhibited accelerated degradation under these harsher cycling conditions. For comparison, the performance of the Zn foil-based cell is displayed in the inset graphs. Surface images of the Zn anodes after cycling at 30% and 50% DOD are also presented in Figs. S36 and S37. Furthermore, Zn@AGI even remained stable at 5 mA cm^−2^ and 0.5 mAh cm^−2^, confirming robust high-rate operation (Fig. S38). Lastly, as shown in Fig. 6h, Zn@AGI displayed the most stable voltage profiles, exhibiting the smallest overpotential variations across current densities ranging from 0.5 to 5 mA cm^−2^. Collectively, these results underscore the outstanding electrochemical advantages of the Zn@AGI anode in terms of Coulombic efficiency, cycling stability and morphological robustness.

**Fig. 7:**
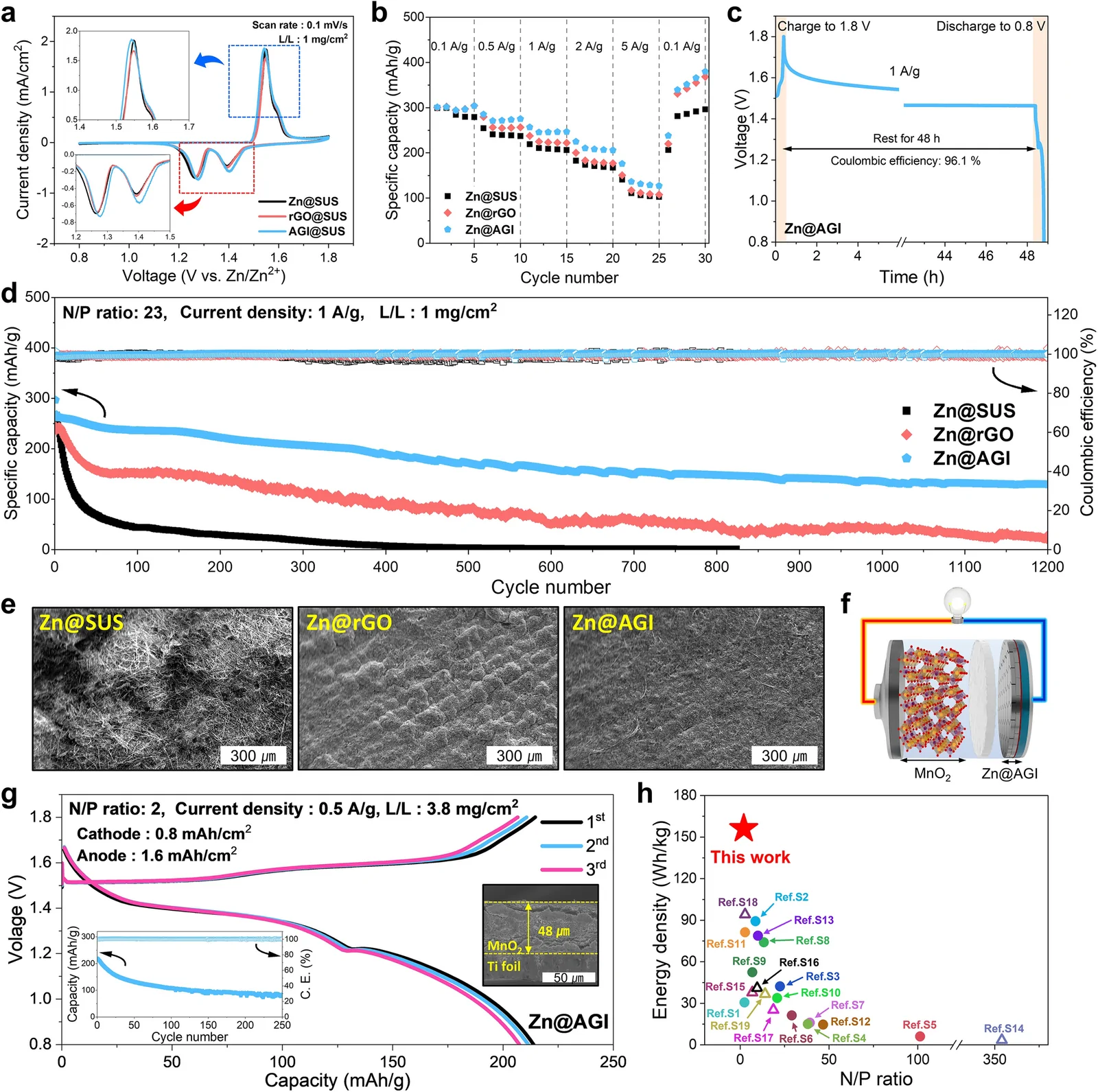
**a** Cyclic voltammetry (CV) curves of full-cells employing α-MnO_2_ as the cathode and Zn@SUS, Zn@rGO or Zn@AGI as the anode, measured at a scan rate of 0.1 mV s^−1^. **b** Rate performance of full cells cycled at varying current densities (0.1, 0.5, 1, 2, 5 and back to 0.1 A g^−1^). **c** Monitoring the self-discharge behavior of the full cell by charging to 1.8 V, resting for 48 h and then discharging to 0.8 V. **d** Long-term cycling performance of the full cells with an *N*/*P* ratio of 23, evaluated at a current density of 1 A g^−1^. **e** SEM surface images of the three Zn anodes after 500 cycles. **f** Schematic of the full cell configuration. **g** Representative initial three charge/discharge curves of the full cell employing Zn@AGI anode with an ultralow N/P ratio of 2, tested at a current density of 0.5 A g^−1^. The left inset shows the cycling performance of the full cell, while the right inset displays a cross-sectional SEM image of the high-loading MnO_2_ cathode. **h** Ragone plot comparing energy densities of aqueous Zn-ion batteries using diverse Zn anodes as a function of *N*/*P* ratio. Plated Zn and Zn foil anodes are denoted by empty triangles and filled circles, respectively

To extend the evaluation toward practical implementation, full Zn-ion batteries were assembled by pairing the plated Zn anodes with the MnO_2_ cathodes. In this study, nanorod-type MnO_2_ cathodes were synthesized via a hydrothermal method, yielding phase-pure α-MnO_2_ (Fig. S39) [70]. As shown in the cyclic voltammetry (CV) curves in Fig. 7a, all full cells exhibited well-defined redox peaks associated with the electrochemical activity of α-MnO_2_. Notably, Zn@AGI displayed obviously smaller voltage polarization and higher cathodic current responses, suggesting more favorable Zn^2+^/Zn reaction kinetics. Figure 7b compares the rate capabilities of the three cells under different current densities. The cell with Zn@AGI delivered specific capacities of 301.6, 275.3, 246.7, 205.9, 127.2 and 379.8 mAh g^−1^, outperforming the Zn@SUS cell (299.4, 237.3, 206.3, 167.4, 102.5 and 296.3 mAh g^−1^) and the Zn@rGO cell (302.2, 256.6, 222.0, 176.8, 107.5 and 368.8 mAh g^−1^) at current densities of 0.1, 0.5, 1, 2, 5 and 0.1 A g^−1^, respectively (Fig. S40). Given that the rate capability test was conducted during the early cycling, the observed capacity discrepancy with increasing current density is particularly noteworthy, highlighting the critical roles of fast reaction kinetics and interfacial stability of Zn@AGI. These differences are expected to become more pronounced upon prolonged cycling. Due to the reactive nature of the aqueous electrolyte, AZIBs are susceptible to degradation and charge loss over time, primarily caused by parasitic reactions such as Zn corrosion and ZHS formation, even when not in use. Accordingly, a self-discharge test was performed to assess the battery stability by monitoring the open-circuit voltage (OCV) of the cell after 10 cycles, followed by charging to 1.8 V, resting for 48 h and then discharging to 0.8 V. The full cell with Zn@AGI retained 96.1% of its initial capacity, demonstrating excellent resistance to self-discharge and parasitic reactions. In contrast, cells incorporating Zn@rGO and Zn@SUS exhibited lower capacity retention and a complete discharge over the same resting period, respectively, highlighting the critical role of AGI in stabilizing the electrode–electrolyte interface (Fig. S41). The long-term cycling stability was further evaluated at 1 A g^−1^ in full cells (*N*/*P* = 23) employing Zn anodes with 5 mAh cm^−2^ (Fig. 7d). The full cell with Zn@AGI greatly outperformed the control cells, revealing that the uniformly plated Zn anode with high corrosion resistance is critical for achieving long-term performance. Moreover, it delivered a higher capacity than the Zn foil-based full cell throughout the cycling test, even though the latter employed the *N*/*P* ratio as high as 680 (Fig. S42). Such enhancement is attributed to the stable reversibility of Zn@AGI, while suppressing electrochemical degradation that readily occurs in conventional Zn foil anodes. Moreover, postmortem SEM analysis was conducted to examine the surface morphology of Zn anodes after 500 cycles (Fig. 7e). While Zn@SUS showed severe dendritic growth with deposits penetrating the separator, Zn@rGO exhibited pronounced protrusions of overgrown dendrites. In contrast, Zn@AGI retained a smooth and uniform surface over prolonged cycling. To assess its practical viability, a full cell was assembled by pairing a high-loading MnO_2_ cathode (3.8 mg cm^−2^) with a low-capacity plated Zn@AGI anode, thereby reaching an ultralow *N*/*P* ratio of 2 (Fig. 7f). Despite these stringent conditions, the cell achieved a high energy density of 156.1 Wh kg^−1^ (based on the full electrode) in the initial cycles and maintained stable cycling over 250 cycles, outperforming recent studies (Fig. 7g–h and Table S3). Under lean-Zn configurations, reducing the *N*/*P* ratio enhances energy density but simultaneously amplifies irreversible Zn loss arising from parasitic reactions and morphology-induced degradation, thereby rendering anode stability more critical than Zn excess. Accordingly, the superior performance of the Zn@AGI full-cell originates from its nanoscale interfacial architecture, which enables (002)-textured planar Zn growth, spatially uniform nucleation and suppressed corrosion/by-product formation. These features collectively establish a stable interface for uniform Zn plating, highlighting the role of interfacial design alongside current density effects, and account for the high energy density achieved at an *N*/*P* ratio of 2. Moreover, the BCP template-enabled interfacial design is readily extendable to large-area electrodes, underscoring its scalability and practical relevance. To further examine the intrinsic stability of the Zn@AGI anode under practically relevant conditions, a ZnI_2_‖Zn@AGI full-cell was additionally evaluated at 1 C with a realistic areal capacity (~ 3.3 mAh cm^−2^) and a lean *N*/*P* ratio of 1.51 (Fig. S43). The stable cycling behavior observed in this configuration confirms that the Zn@AGI anode is not the limiting factor governing full cell performance.

## Conclusions

In summary, we developed a nanoscale interfacial architecture that facilitates uniform Zn plating and directional growth, enabling dendrite-free and highly reversible Zn anodes for high-performance AZIBs with long-term stability. This interfacial structure was realized by solvent-annealed BCP templating and the selective reduction of gold precursors to construct well-ordered Au nanoseed arrays on the rGO nanolayer. By leveraging BCP-templated Au nanoseed arrays on rGO, AGI facilitated spatially regulated nucleation and preferential (002)-oriented growth during Zn plating, thereby producing dense, planar, horizontally layered Zn anodes with a pronounced (002) texture. The underlying mechanism was elucidated by combined experimental observations and theoretical simulations: in Zn@AGI, the Au-modified interface generates a homogeneous Zn^2+^ flux and guides texture development (preferentially along (002)), both of which are critical for directing initial nucleation and sustaining planar Zn growth. To evaluate their electrochemical performances, Zn anodes were systematically tested in a variety of cell configurations, including asymmetric cells, symmetric cells and full cells. Notably, Zn@AGI exhibited outstanding long-term cycling stability in symmetric cells, retaining performance for over 3000 h at 1 mA cm^−2^ and 10% DOD, and also maintained stable operation under higher-DOD conditions (30% and 50%), significantly outperforming other Zn anodes. Furthermore, under stringent conditions with high cathode loading and the ultralow *N*/*P* ratio of 2, the full cell with Zn@AGI achieved the energy density of 156.1 Wh kg^−1^ full electrode, far exceeding the performance of previously reported AZIBs using foil and plated Zn anodes. Collectively, these findings highlight the practical viability and scalability of the proposed interfacial strategy, which facilitates highly controlled, directionally guided Zn plating, thereby offering a viable pathway for advanced aqueous Zn-ion batteries.

## Supplementary Information

Below is the link to the electronic supplementary material.

Supplementary file1 (DOCX 13.1 MB)
