# Supplementary material for: Growth-Directing Nanostructured Interfaces via Block Copolymer-Templated Au Nanoseed Arrays for Stabilized Zinc Anodes in Lean-Zinc Batteries
Source: Nanomicro Lett. 2026 May 6;18:355. doi: 10.1007/s40820-026-02167-y (PMC13149798; doi:10.1007/s40820-026-02167-y)
Supplement: Supplementary file 1 — Supplementary file1 (DOCX 13.1 MB) [file 40820_2026_2167_MOESM1_ESM.docx]

**Supporting information**

**Growth-Directing Nanostructured Interfaces via Block Copolymer-Templated Au Nanoseed Arrays for Stabilized Zinc Anodes in Lean-Zinc Batteries**

Dong Won Hae^1,6,†^ , Hoseok Lee^3,4, †^, Hyeong Jun Kook^1,2^, Ga Hee Kim^1,2^, Saehun Kim^1^, Jaecheol Choi^1^, Min Pyeong Kim^1^, Seok Hun Kang^1^, Won Jun Lee^6^, Young-Gi Lee^1^, Hyeong Min Jin^7,^*, Jongsoon Kim^3,4,5,^*, and Dong Ok Shin^1,2,^*

^1^ Smart Materials Research Section, Electronics and Telecommunications Research Institute (ETRI), Daejeon 34129, Republic of Korea

^2^ Department of Semiconductor and Advanced Device Engineering, University of Science and Technology (UST), Daejeon 34113, Republic of Korea

^3^ Department of Future Energy Engineering, Sungkyunkwan University, Suwon 16419, Republic of Korea

^4^ SKKU Institute of Energy Science and Technology (SIEST), Sungkyunkwan University, Suwon 16419, Republic of Korea

^5^ Department of Energy Science, Sungkyunkwan University, Suwon 16419, Republic of Korea

^6^ Department of Fiber System Engineering, Dankook University, Yongin 16890, Republic of Korea

^7^ Department of Organic Materials Engineering, Chungnam National University, Daejeon 34134, Republic of Korea

*Corresponding author. E-mail: hyeongmin@cnu.ac.kr (Prof. Hyeong Min Jin), jongsoonkim@skku.edu (Prof. Jongsoon Kim), doshin@etri.re.kr (Dr. Dong Ok Shin)

† D.W. Hae, H. Lee contributed equally to this work


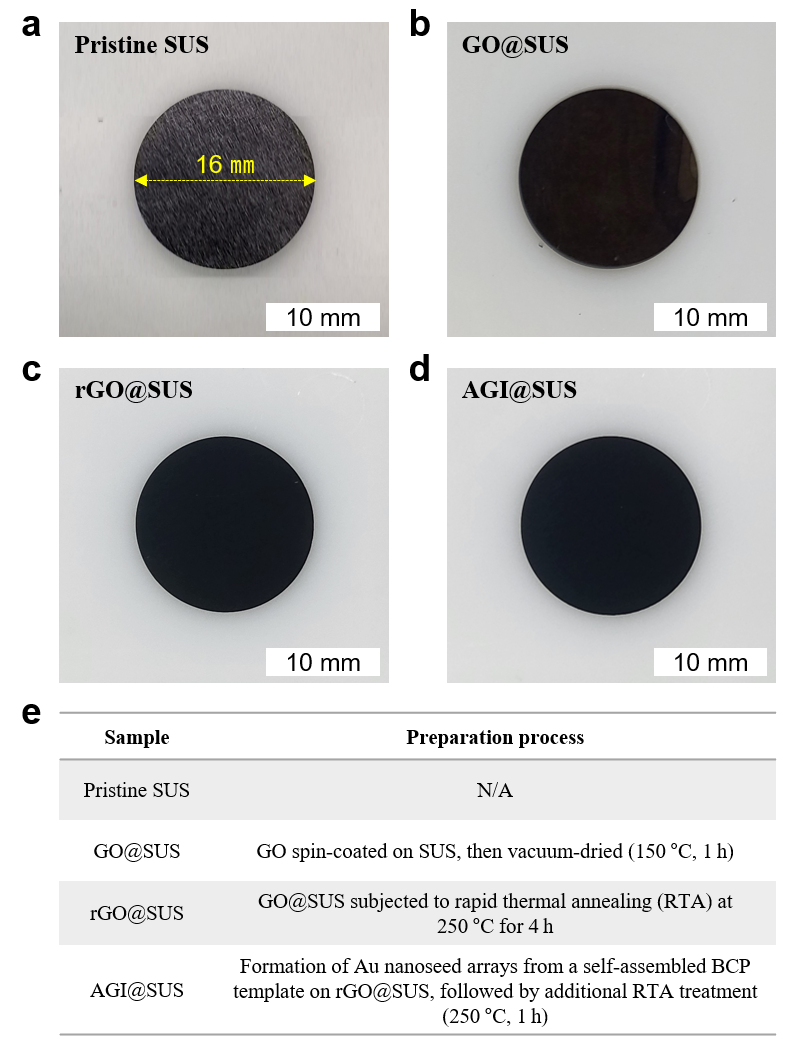


**Figure S1.** Digital camera images of different substrates: (a) pristine SUS, (b) GO-coated SUS, (c) rGO-coated SUS, and (d) Au nanoseed–rGO interface (AGI)-coated SUS. (e) Table summarizing the preparation process of each substrate.


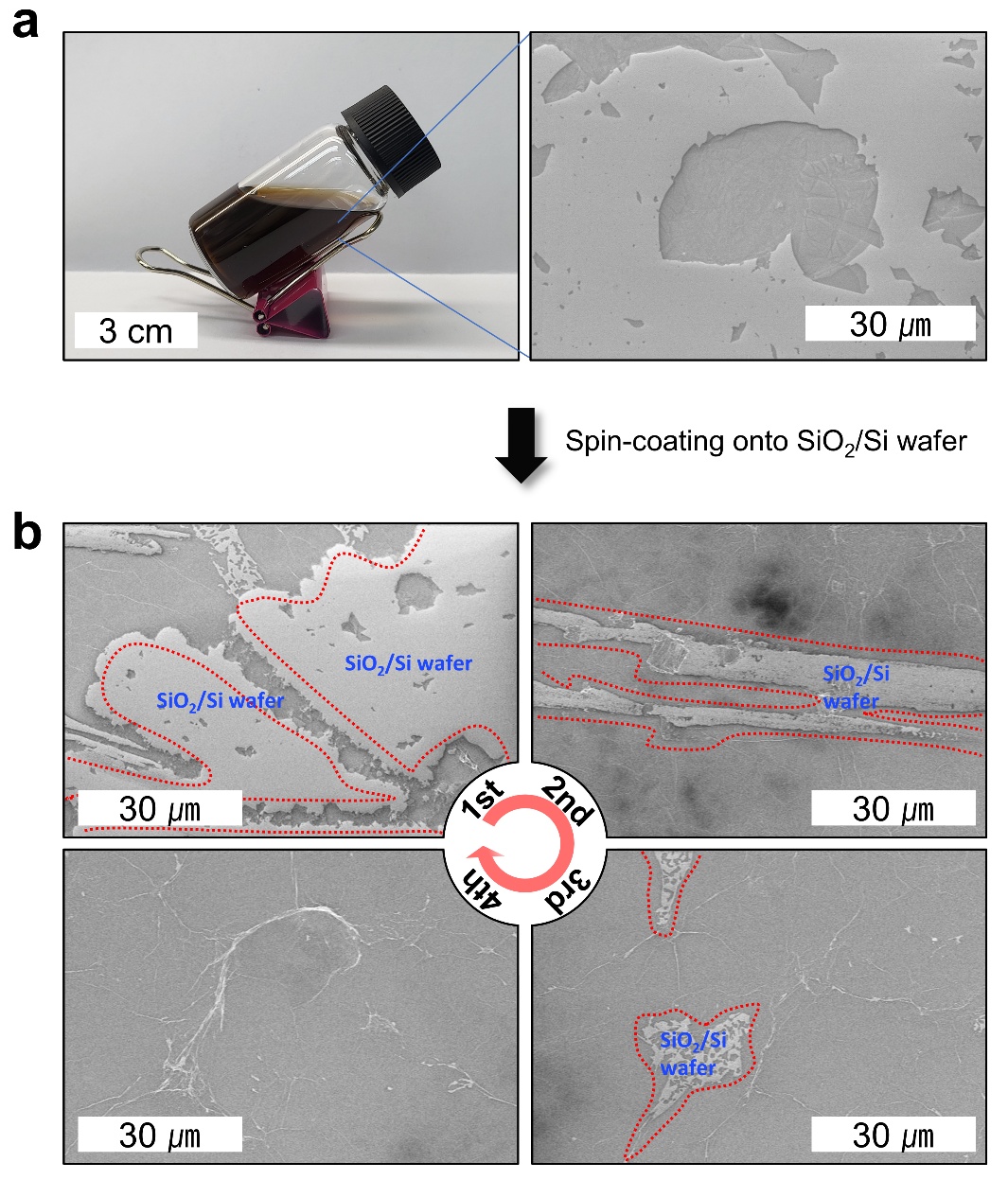


**Figure S2.** (a) Digital camera image of the GO dispersion (1 mg/mL) used for GO nanolayer fabrication along with a corresponding SEM image of an individual GO flake. For convenience, a SiO_2_/Si wafer was used for analysis. (b) GO nanolayer fabrication via multiple spin-coating steps. After four successive spin-coating cycles, the SiO_2_/Si wafer was fully covered with a uniform GO nanolayer. The same spin-coating conditions were successfully applied to the SUS substrate.


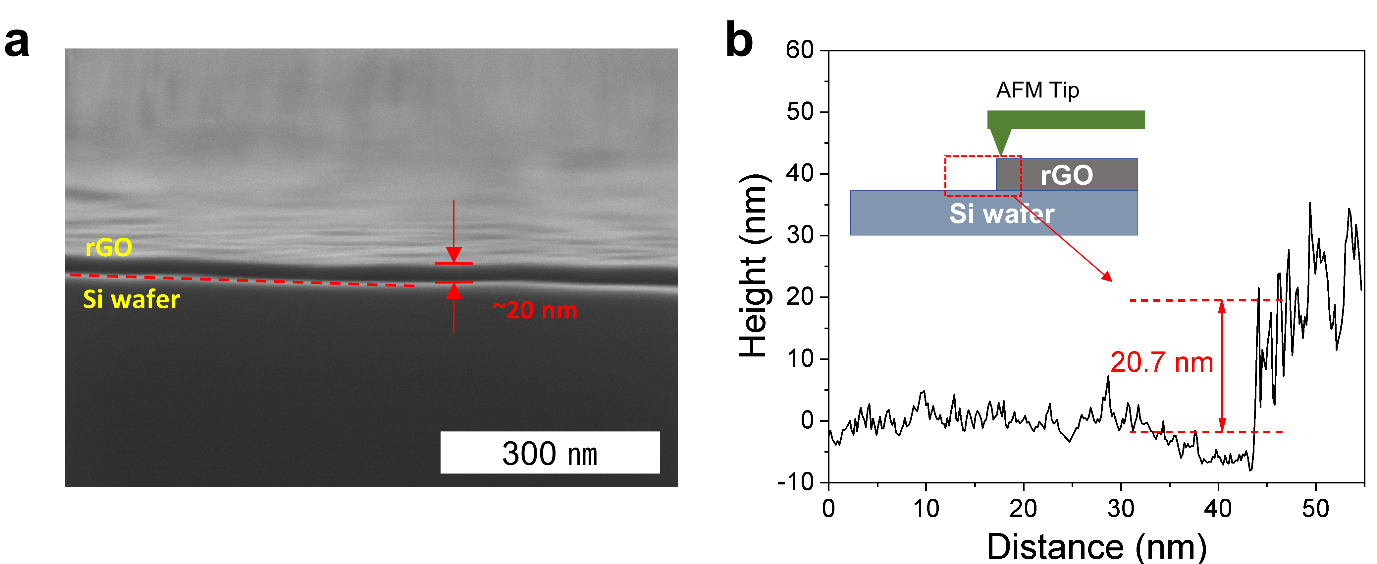


**Figure S3.** (a) SEM image showing the rGO nanolayer with a thickness of ~20.0 nm. (b) AFM measurement at the edge of the rGO nanolayer on a Si wafer, confirming a thickness of ~20.7 nm in agreement with the SEM result shown in (a).


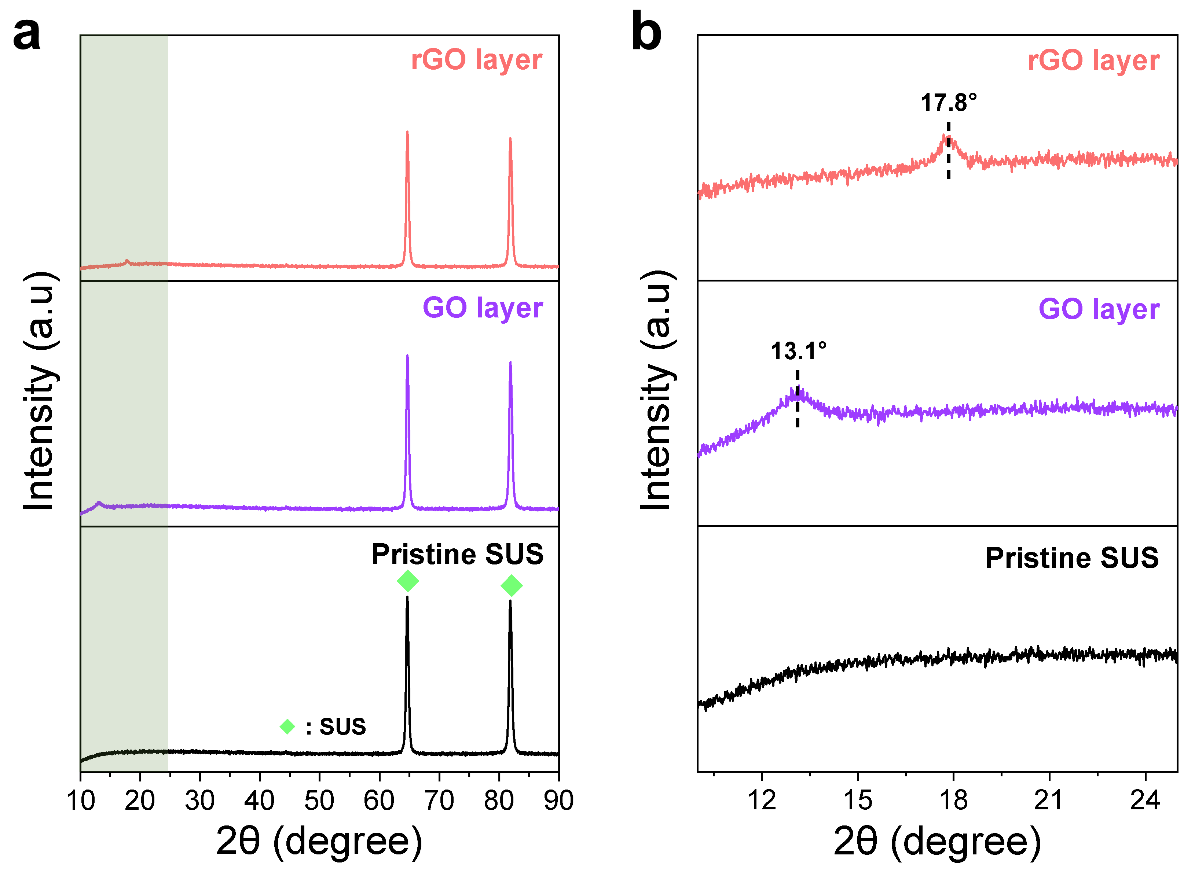


**Figure S4.** (a) XRD patterns of pristine SUS, GO nanolayer (vacuum-dried at 150 °C for 1 h), and rGO nanolayer (GO nanolayer subjected to RTA at 250 °C for 4 h). (b) Enlarged view of region marked by the light green box in (a). Notably, the rGO nanolayer exhibits a more compact and well-stacked structure due to RTA treatment, as evidenced by a peak shift toward higher 2θ compared to the simply vacuum-dried GO nanolayer.


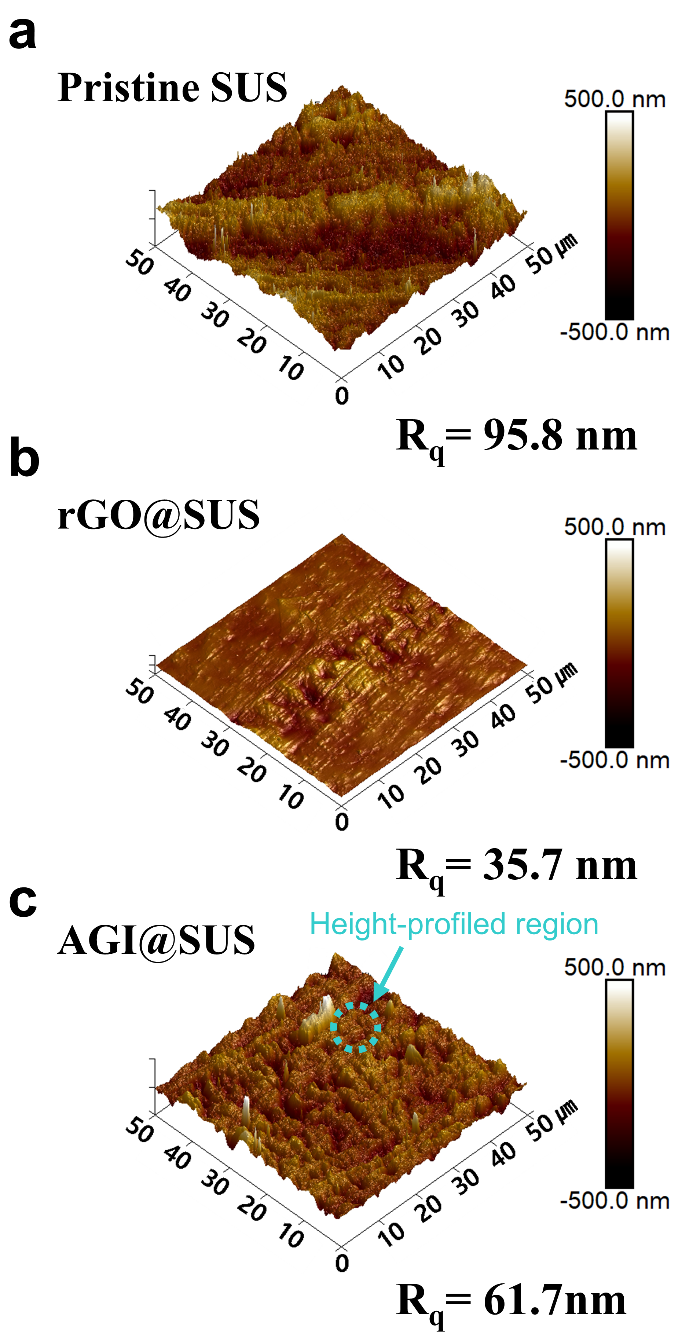


**Figure S5.** AFM images over an area of 50×50 μm^2^ for (a) pristine SUS, (b) rGO@SUS, and (c) AGI@SUS. From the corresponding AFM measurements, root-mean-square roughness (R_q_) values were obtained for each sample. The height-profiled region marked by the light green dotted circle in (c) corresponds to the area shown in Figure 1g.


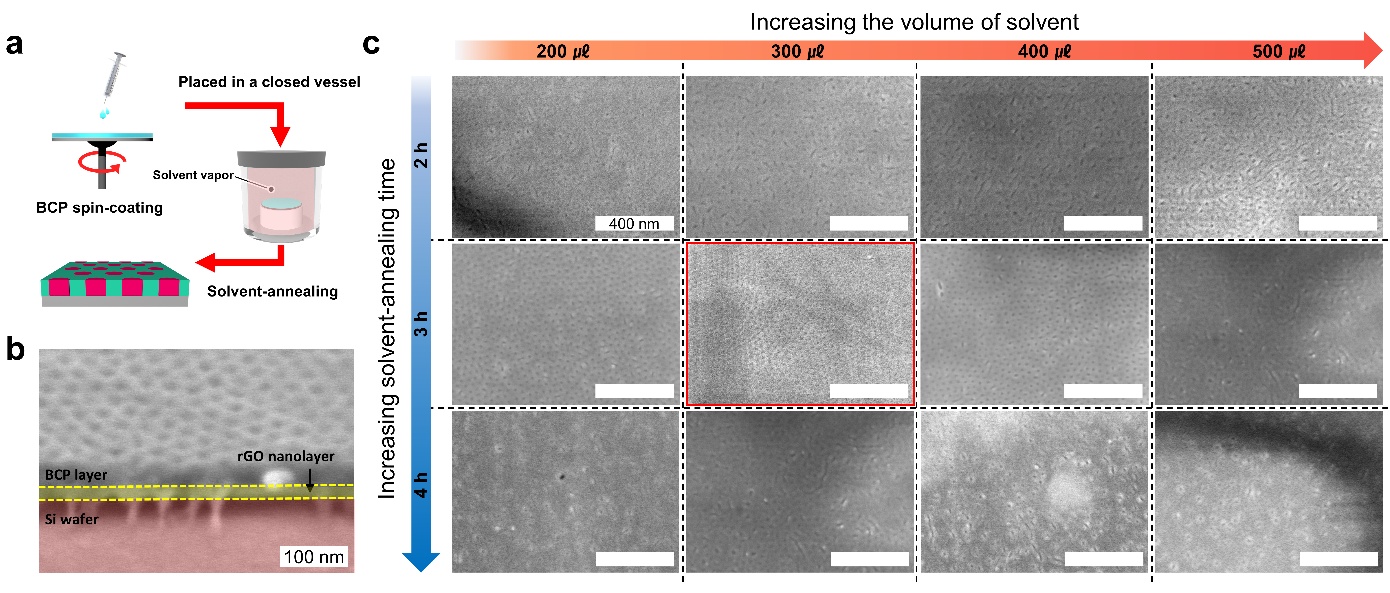


**Figure S6.** (a) Schematic illustration depicting the self-assembly of a PS-*b*-P4VP thin film driven by solvent-annealing. (b) SEM image showing the thickness of the self-assembled PS-*b*-P4VP template. For precise measurement, the BCP template was prepared on a Si wafer. (c) Morphological changes in solvent-annealed PS-*b*-P4VP templates on rGO@SUS substrates with varying solvent volume and annealing time. As revealed by SEM images, annealing time is more crucial than solvent volume in directing self-assembled nanostructure formation. Accordingly, the optimum solvent-annealing condition was set to 3 h and 300 μL in this study, as confirmed by the SEM image highlighted by the red box.


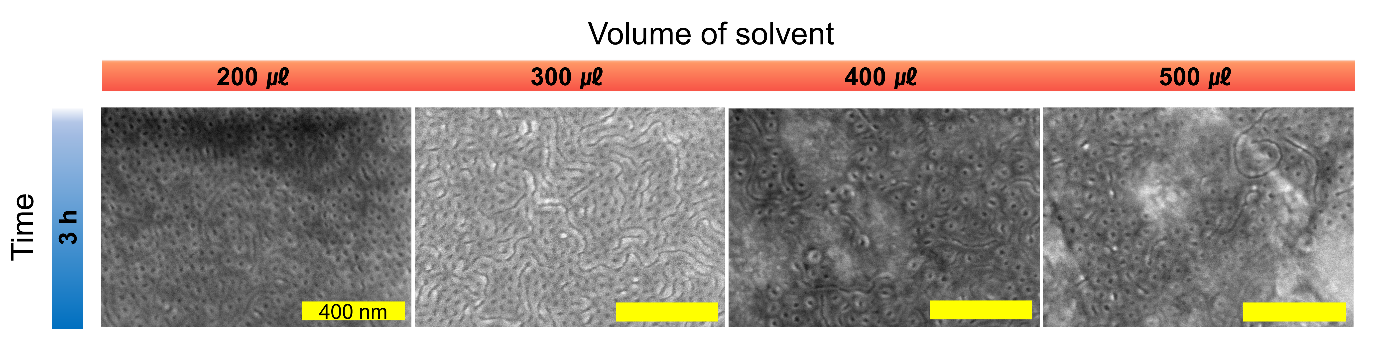


**Figure S7.** Surface morphology of the BCP template solvent-annealed for 3 h on the pristine SUS substrate (without the rGO nanolayer) as a function of solvent volume. Owing to the intrinsic roughness of SUS, the lateral ordering and uniform microphase separation of the BCP were significantly disrupted.


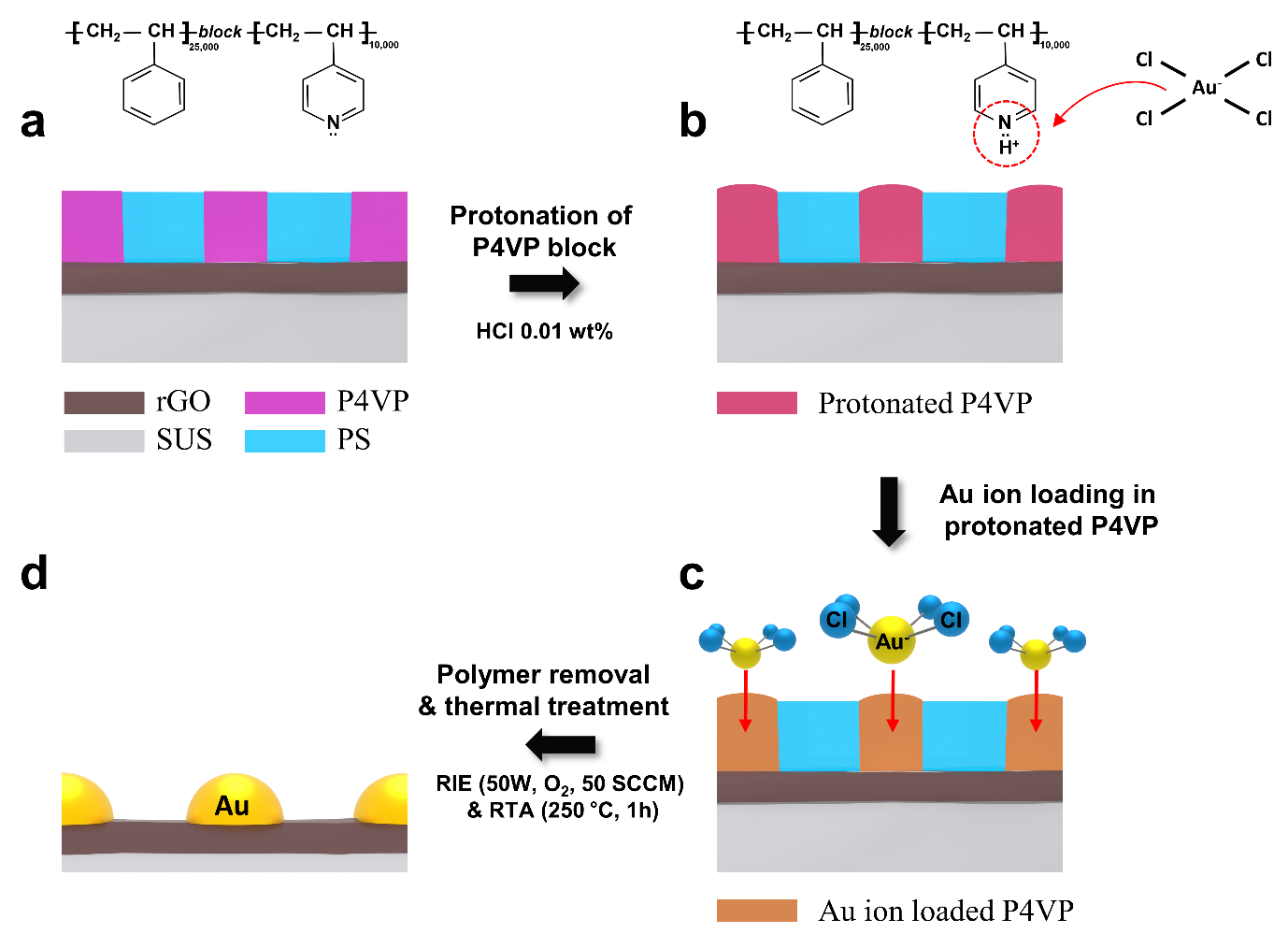


**Figure S8.** Schematic illustration of the fabrication process of AGI@SUS. (a) Preparation of a hexagonally packed cylindrical nanopattern template via solvent-annealing-driven self-assembly of PS-*b*-P4VP. In this system, the lower molecular weight block, P4VP, forms cylindrical nanodomains surrounded by the matrix of the higher molecular weight block, PS. (b) Protonation of the P4VP block by immersing the BCP template in a weakly acidic aqueous solution (0.01 wt% HCl). Notably, the acid-stimulated P4VP block exhibits slight vertical swelling, resulting in a broader lateral diameter in the final Au nanoseeds than that in the initial P4VP cylinders. (c) Au ion loading (1 mM HAuCl_4_ in 0.01 wt% HCl (aq.)) through strong electrostatic interaction between AuCl_4_^-^ and the protonated pyridine units. (d) Formation of Au nanoseed arrays on the rGO@SUS substrates via RIE, followed by RTA treatment.


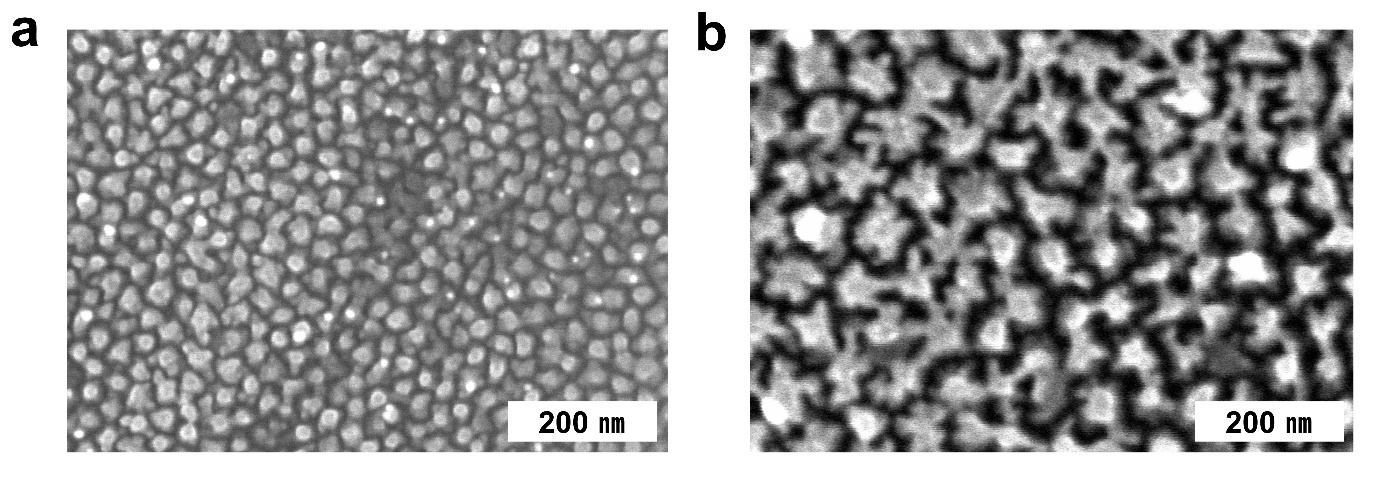


**Figure S9.** Morphology of the fabricated Au nanoseed arrays as a function of Au ion loading time. (a) Au nanoseed arrays after 60 s AuCl_4_^-^ anion loading. (b) Au nanoseed arrays after 300 s AuCl_4_^-^ anion loading. As observed, prolonged loading time leads to irregularly shaped nanoseeds and even interconnections between neighboring seeds.


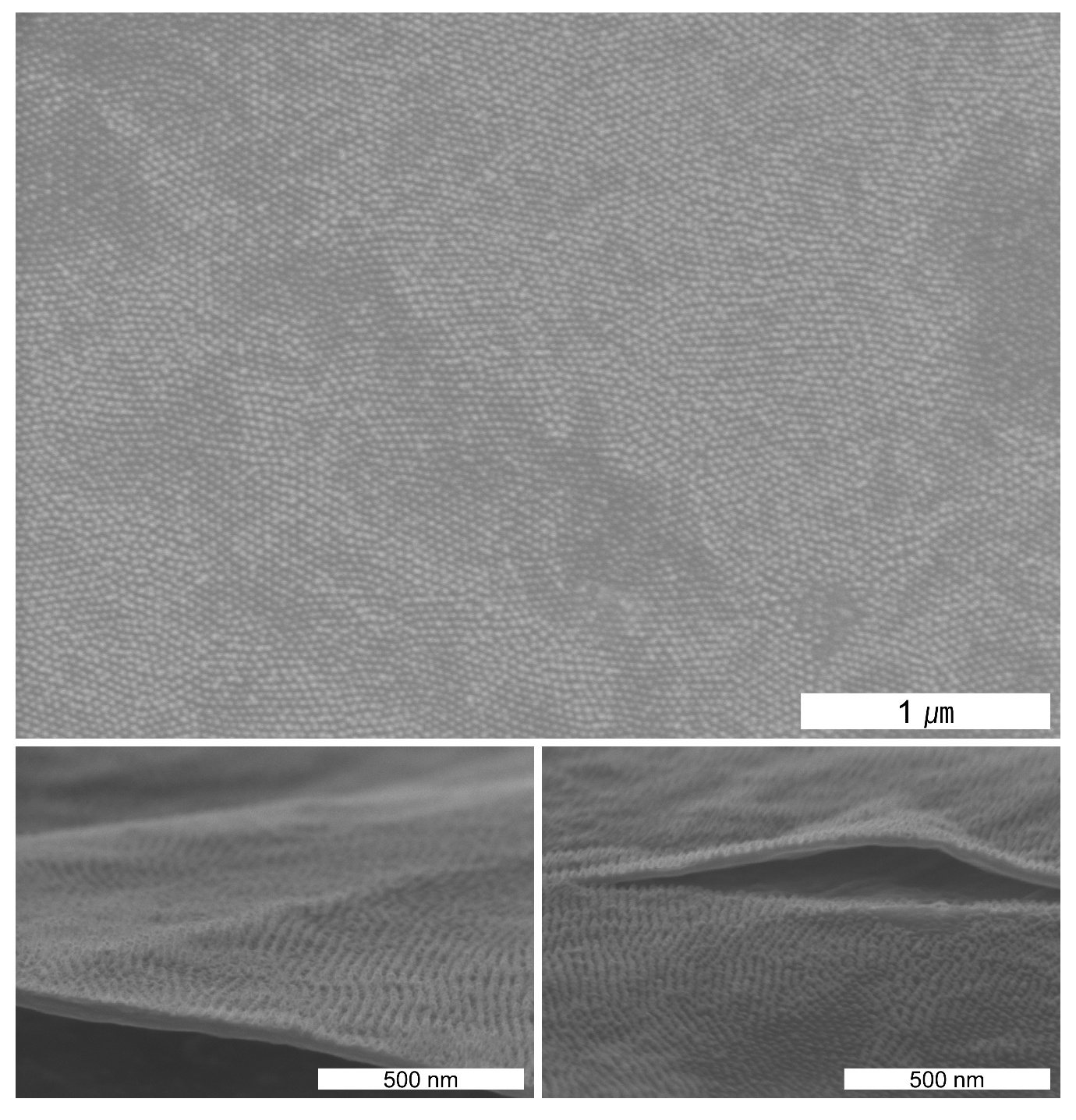


**Figure S10.** The large-area SEM image validates the effectiveness and reliability of the BCP template-based pattern transfer process for the scalable fabrication of Au nanoseed arrays. The region marked by the yellow-dotted circle indicates an area where AGI was peeled off during sample preparation for SEM analysis.


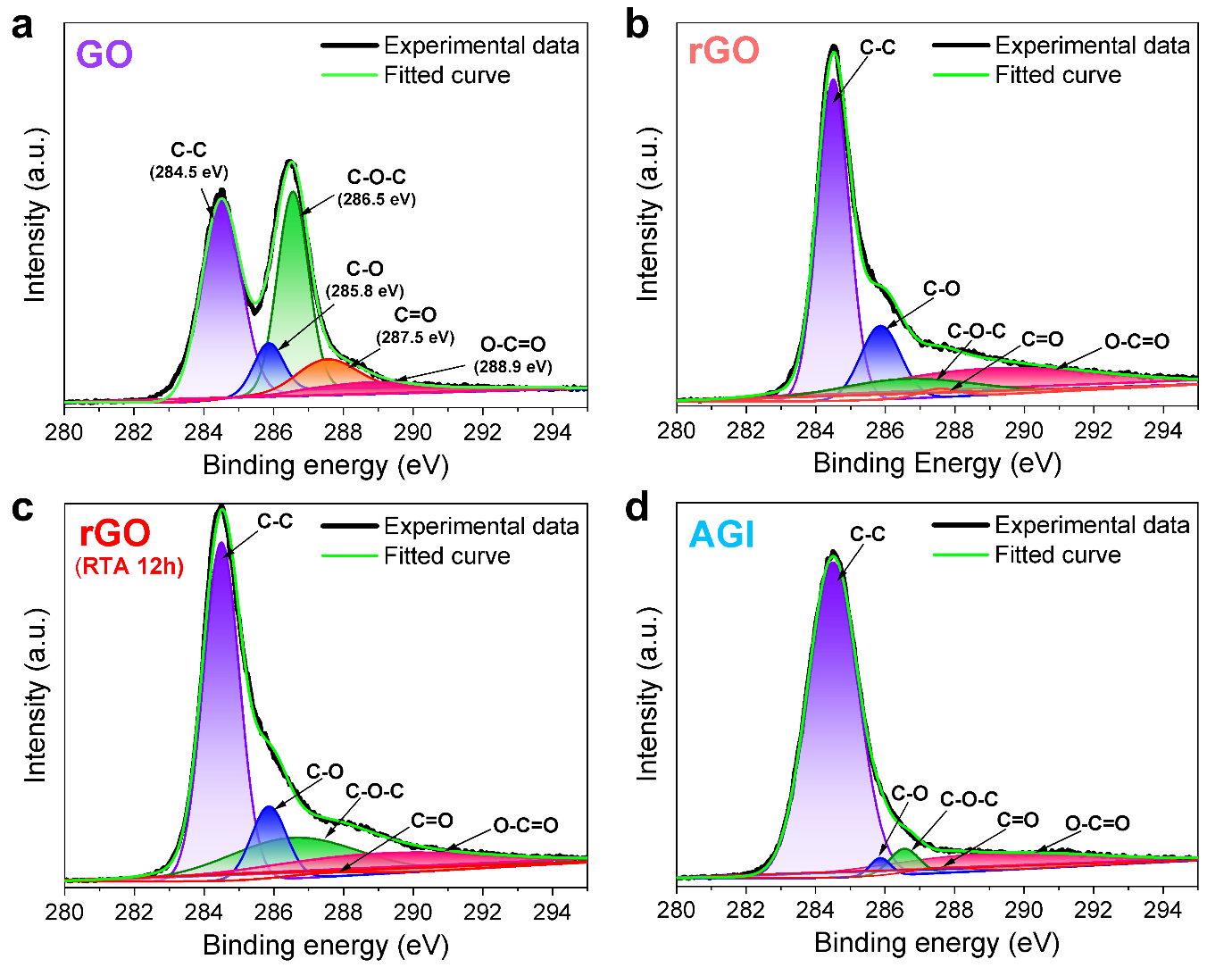


**Figure S11.** High-resolution XPS spectra of C 1s for (a) GO, (b) rGO, (c) rGO after additional thermal treatment at 250 °C for 12 h, and (d) AGI. The spectra reveal the progressive reduction of GO to rGO with thermal treatment. Notably, the C 1s spectrum of rGO after extended thermal treatment remains nearly identical to that of rGO in (b), whereas AGI exhibits a further reduced state. This suggests that the enhanced reduction in AGI originates from the catalytic effect of Au nanoseeds rather than from prolonged thermal treatment.


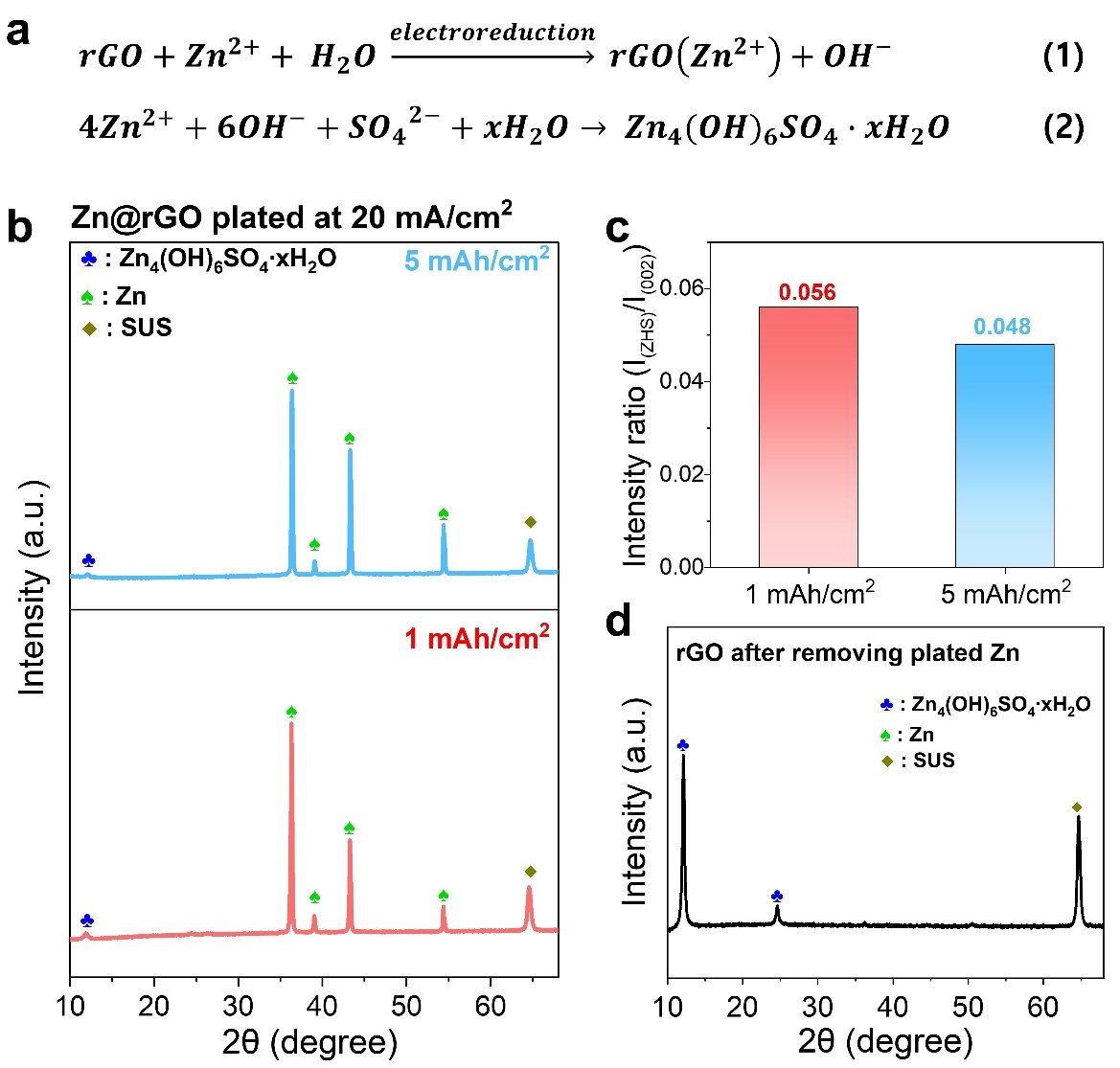


**Figure S12.** (a) Proposed reaction pathway for Zn_4_(OH)_6_SO_4_·xH_2_O (ZHS) formation onto the rGO surface at the initial Zn plating. (b) XRD patterns of Zn@rGO plated at 20 mA/cm^2^ with capacities of 1 and 5 mAh/cm^2^. The signal for ZHS is more noticeable in Zn@rGO with a lower plated capacity. (c) Comparison of the quantified ZHS to Zn (002) peak intensity ratio in XRD patterns for Zn@rGO with capacities of 1 and 5 mAh/cm^2^, indicating a higher relative proportion of ZHS at the lower plated capacity. (d) XRD patterns after intentional removal of plated Zn (20 mA/cm^2^ for 5 mAh/cm^2^) by intensive ethanol rinsing, confirming that the remaining intense ZHS signal originates from the ZHS layers primarily formed on the rGO surface.


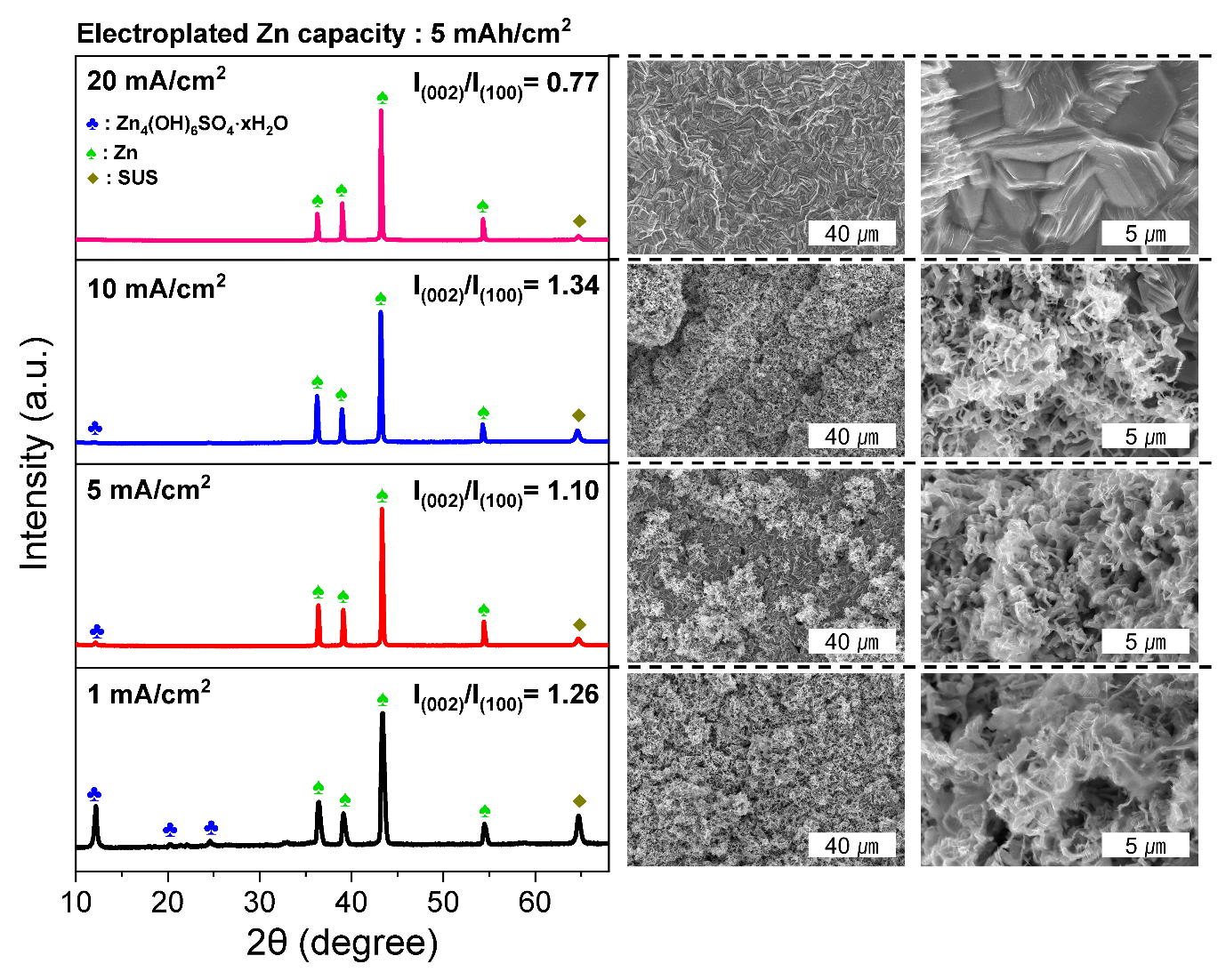


**Figure S13.** XRD patterns and corresponding surface SEM images (wide-view and magnified) of Zn@SUS plated under various current densities (1, 5, 10, and 20 mA/cm^2^), displaying growth facet distribution and surface morphology. In the absence of a growth-guiding interface, plated Zn preferentially grew along the kinetically favored (101) facet, regardless of the applied current density.


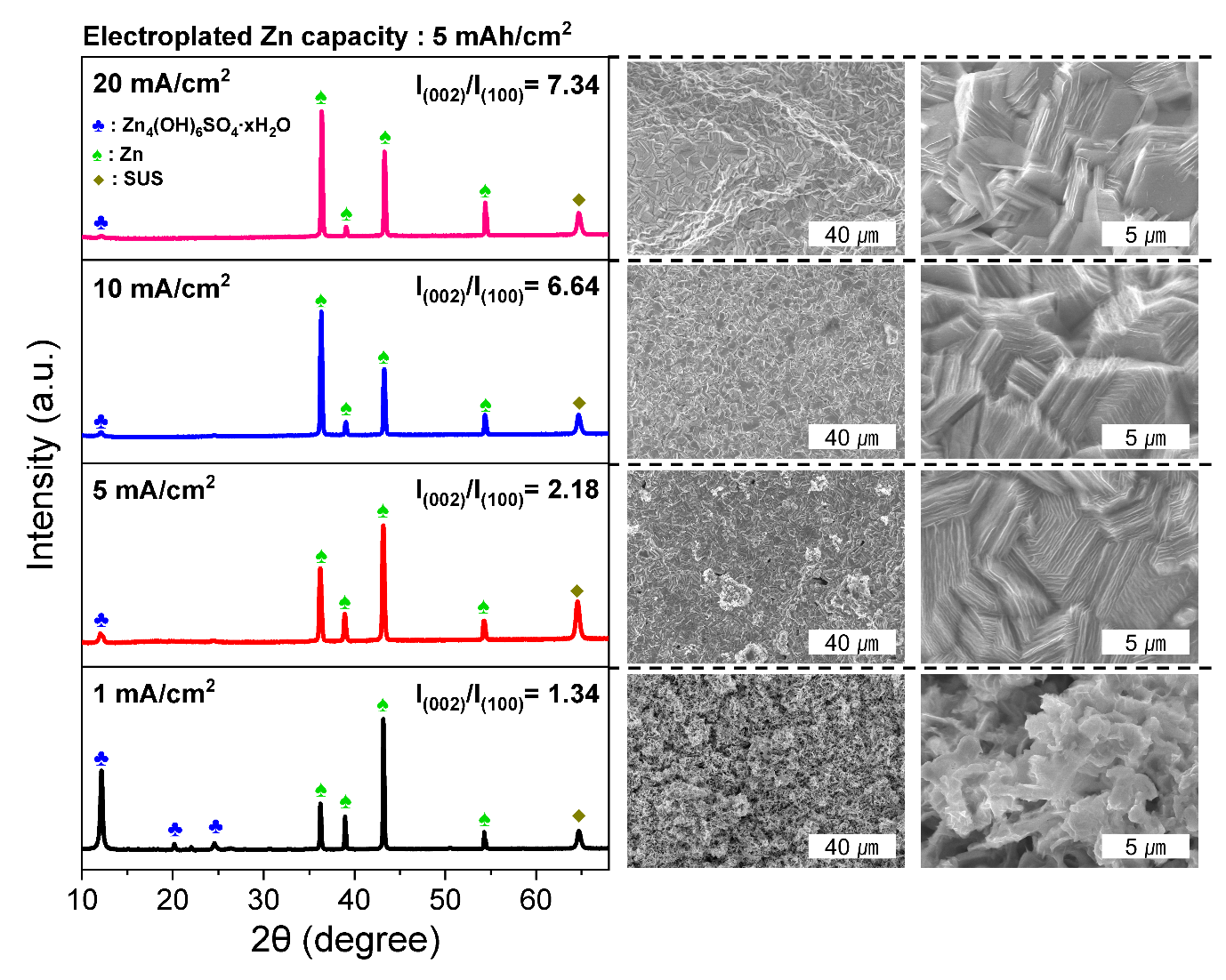


**Figure S14.** XRD patterns and corresponding surface SEM images (wide-view and magnified) of Zn@rGO plated under various current densities (1, 5, 10, and 20 mA/cm^2^), displaying growth facet distribution and surface morphology. As the current density increased, the plated Zn exhibited a tendency toward (002) growth due to its lattice-matched characteristics with the underlying interface. However, a pronounced Zn (101) peak still appeared, leading to an unusual non-horizontal layered Zn growth as shown in magnified SEM image. Moreover, the formation of ZHS layers was more noticeable in Zn@rGO at lower current densities due to the higher reactivity of the exposed (101) facet toward water.


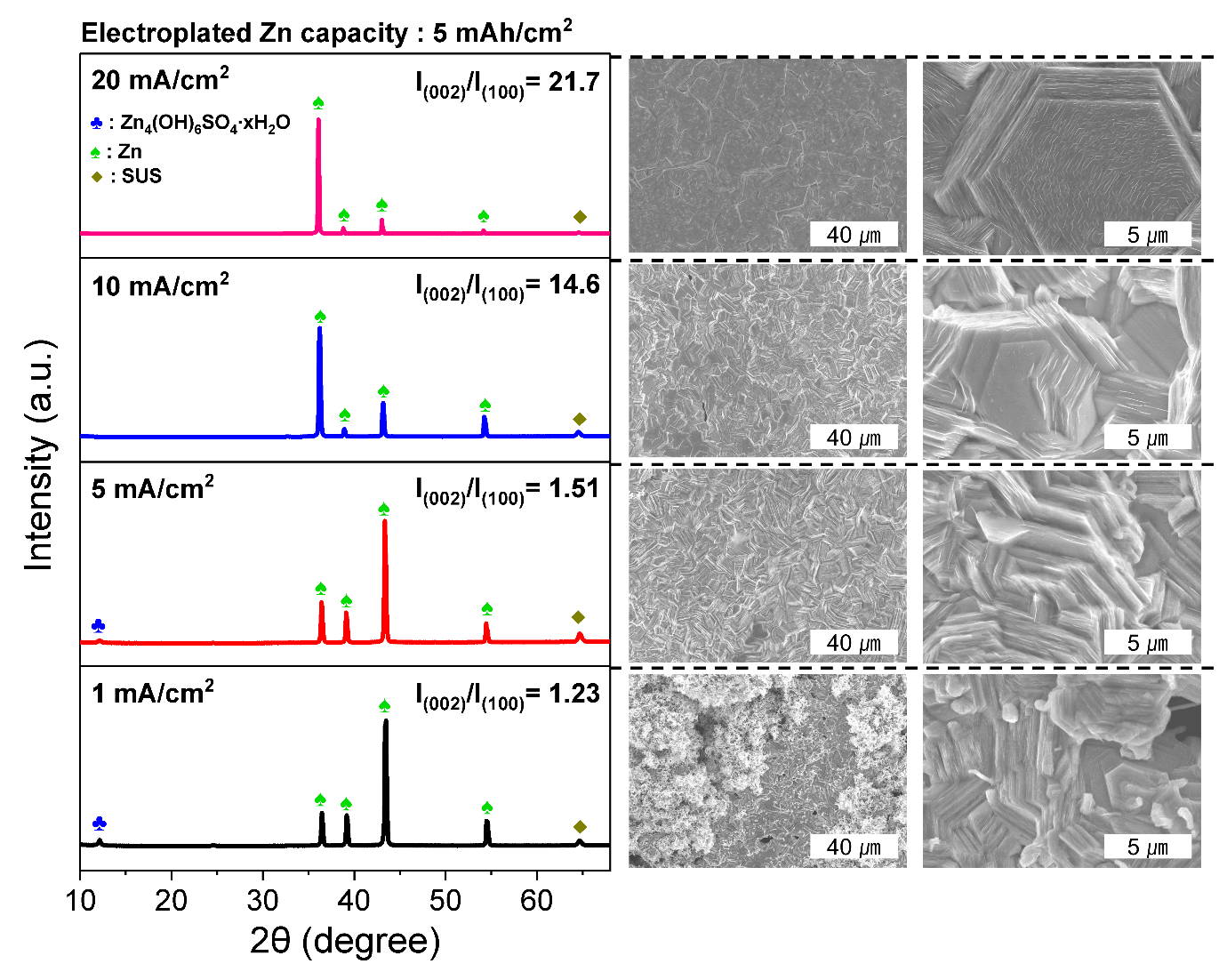


**Figure S15.** XRD patterns and corresponding surface SEM images (wide-view and magnified) of Zn@AGI plated under various current densities (1, 5, 10, and 20 mA/cm^2^), displaying growth facet distribution and surface morphology. As the current density increased, the (002) facet became dominant, accompanied by a gradual decrease in the intensities of the (100) and (101) facets. At 20 mA/cm^2^, horizontally layered Zn morphology with predominant (002) facet was observed, attributed to the homogeneous Zn^2+^ flux and lattice-guided growth enabled by the AGI as clearly confirmed in magnified SEM image.


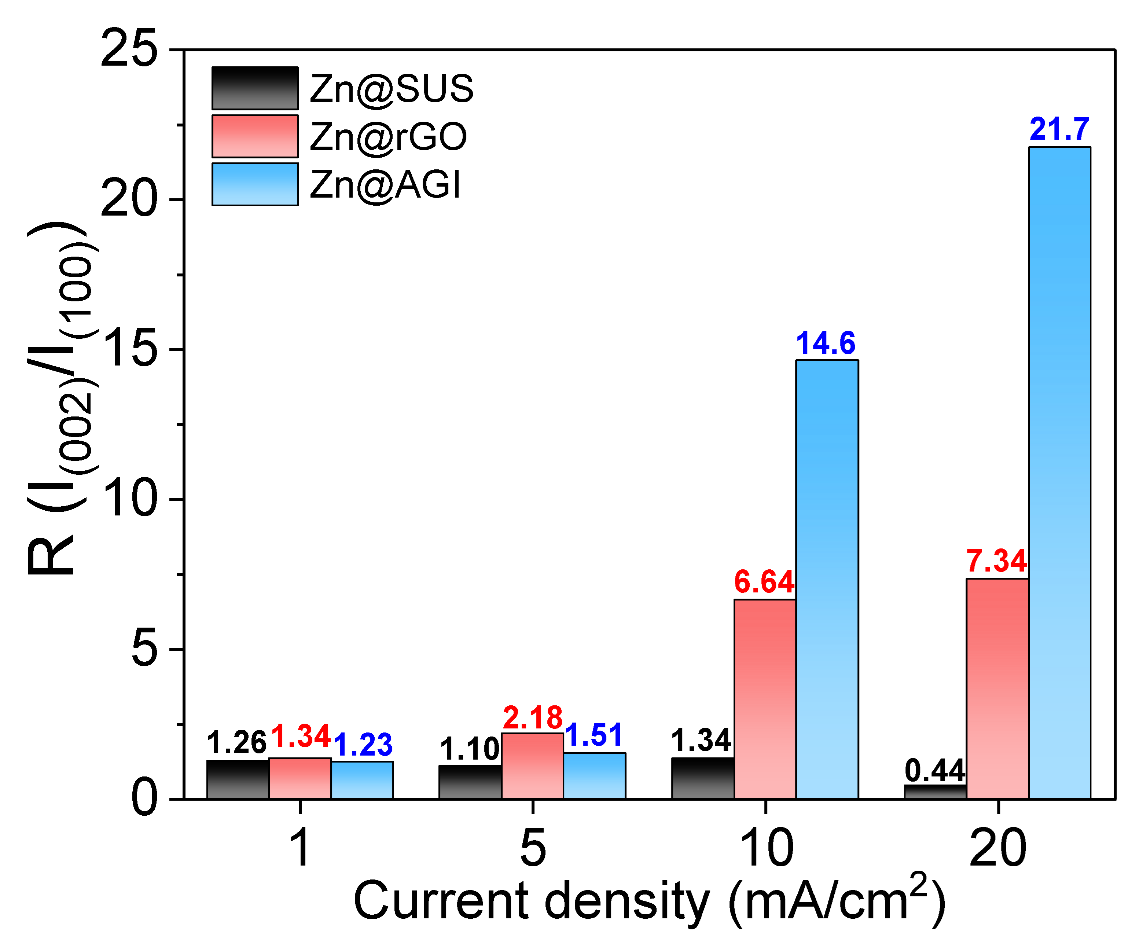


**Figure S16.** Comparison of the orientation factor (R) for different plated Zn anodes as a function of applied current density.


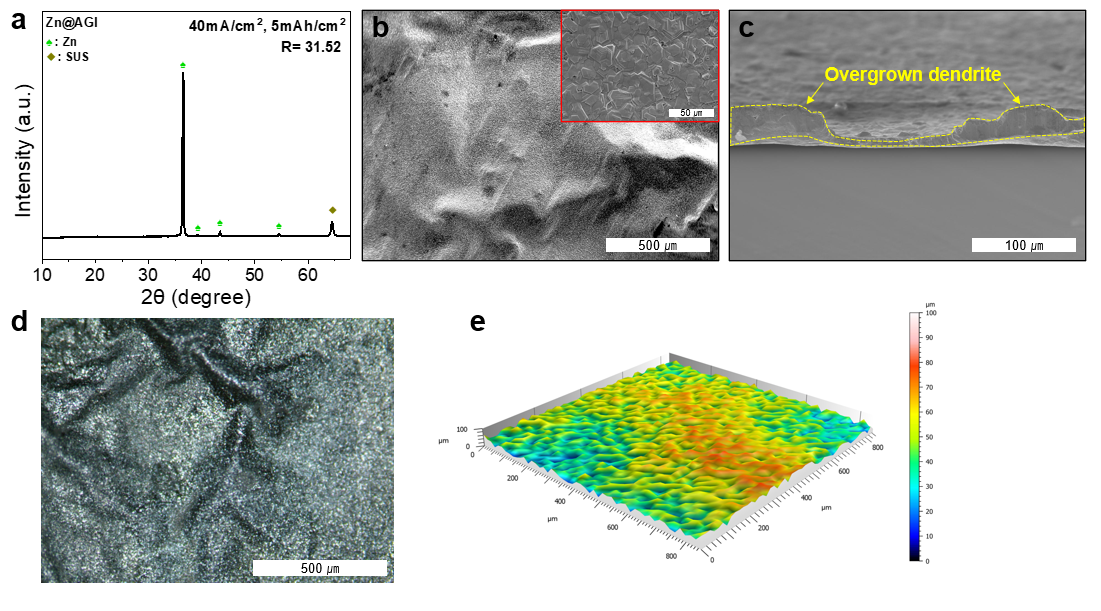


**Figure S17.** (a) XRD pattern of Zn@AGI plated at 40 mA/cm^2^ for 5 mAh/cm^2^ showing a high R value, indicative of (002) preferred orientation at higher current density. (b) Surface and (c) cross-sectional SEM images, (d) optical microscopy (OM) image, and (e) CLSM image, revealing distorted plating morphology of Zn@AGI plated at 40 mA/cm^2^.


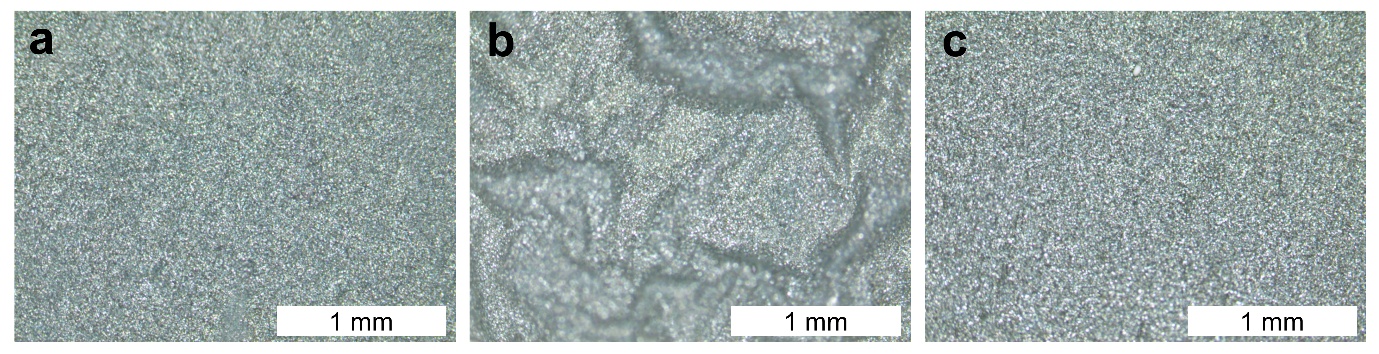


**Figure S18.** Optical microscopy (OM) images showing the surface morphologies of (a) Zn@SUS, (b) Zn@rGO, and (c) Zn@AGI after Zn plating conducted at a current density of 20 mA/cm^2^ to an areal capacity of 5 mAh/cm^2^. While Zn@SUS and Zn@AGI exhibited relatively flat surface features that were not clearly distinguishable by OM analysis alone, Zn@rGO exhibited a ridge-like morphology that was macroscopically discernible.


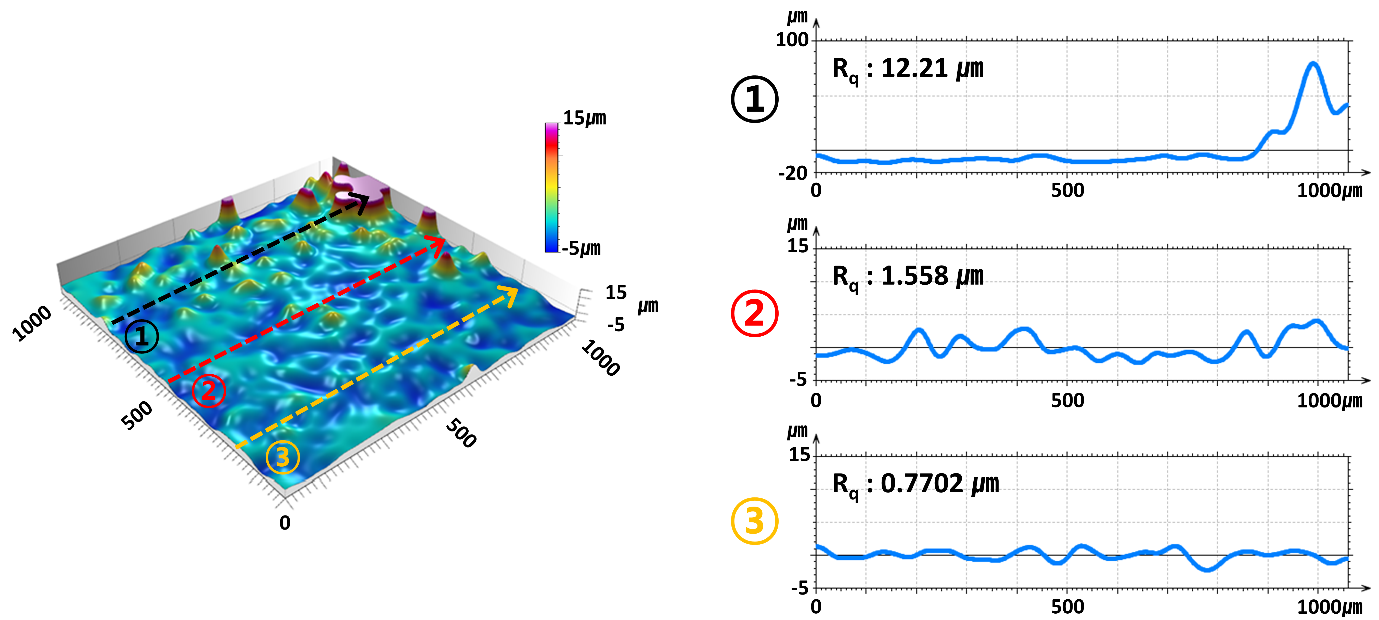


**Figure S19.** CLSM images and surface roughness (R_q_) analysis of commercial Zn foil. Surface scans were performed on three distinct regions to evaluate morphological uniformity and quantify average roughness. The Zn foil exhibited relatively high surface roughness with non-uniform features, highlighting the intrinsically poor smoothness of commercial Zn foil.


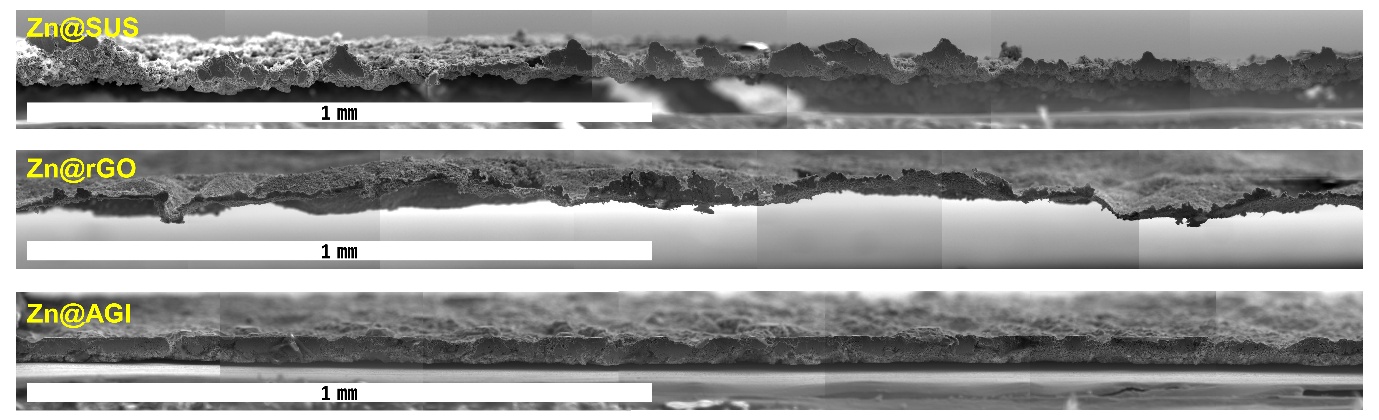


**Figure S20.** Large-area cross-sectional SEM images of different Zn anodes plated at 5 mA/cm^2^ for 5 mAh/cm^2^.


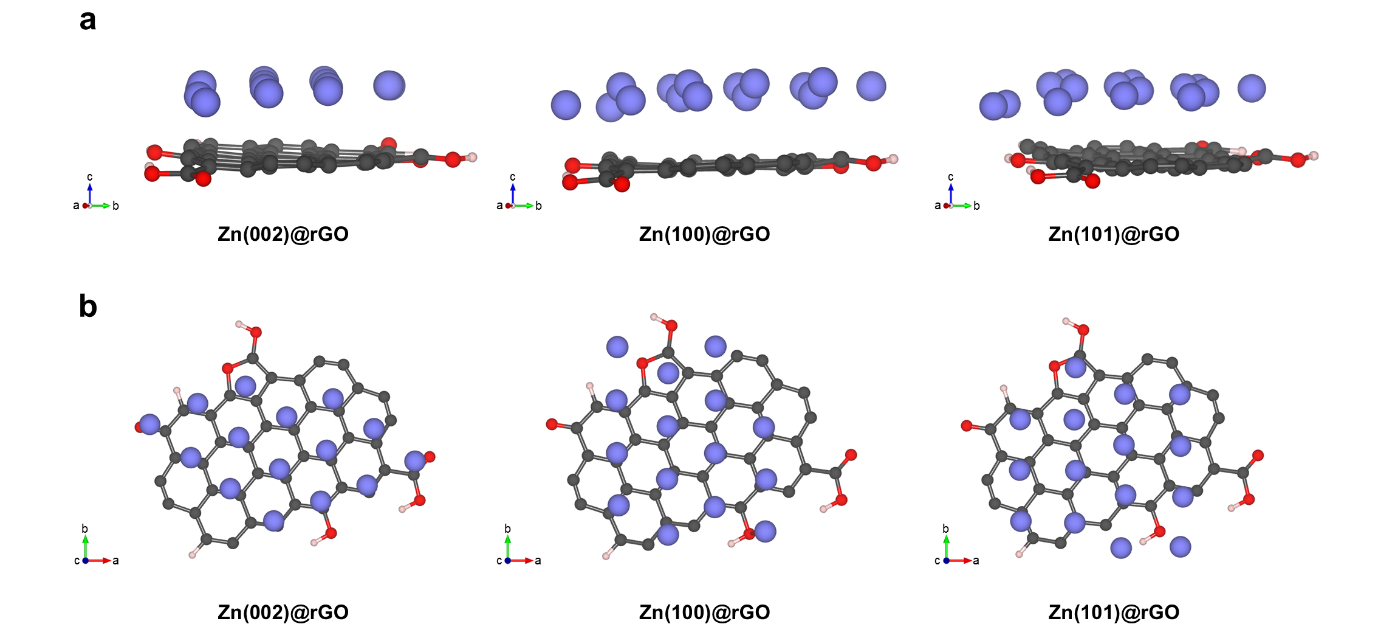


**Figure S21**. Optimized geometries of Zn on rGO for adsorption energy calculations (002), (100), and (101) facets: (a) a-axis projection (b) c-axis projection.


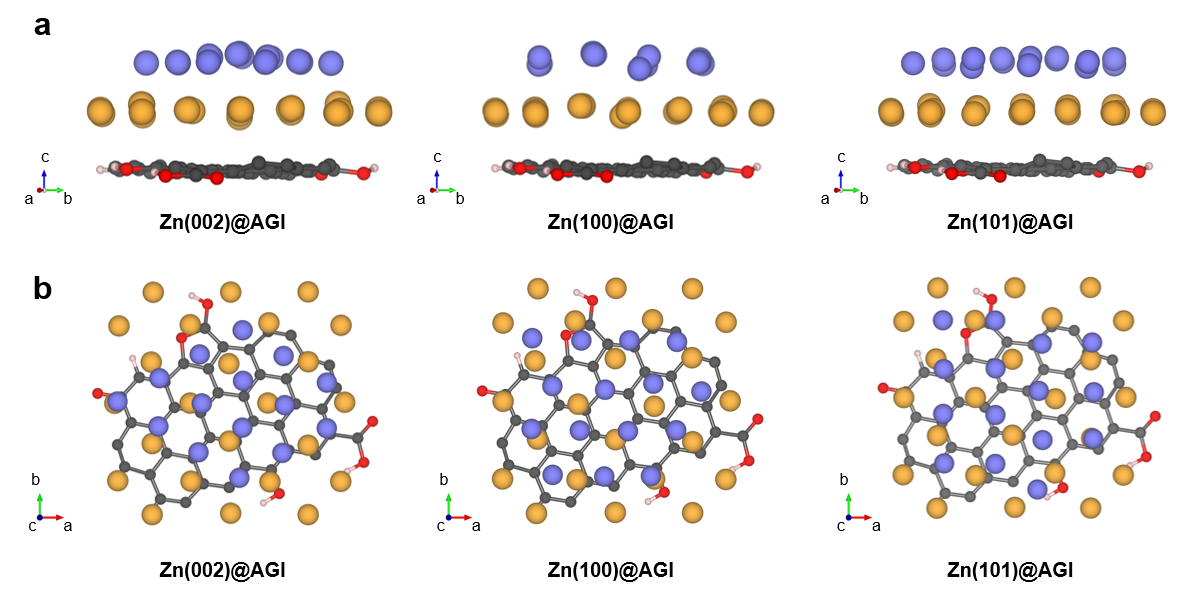


**Figure S22**. Optimized geometries of Zn on AGI for adsorption energy calculations (002), (100), and (101) facets: (a) a-axis projection (b) c-axis projection.


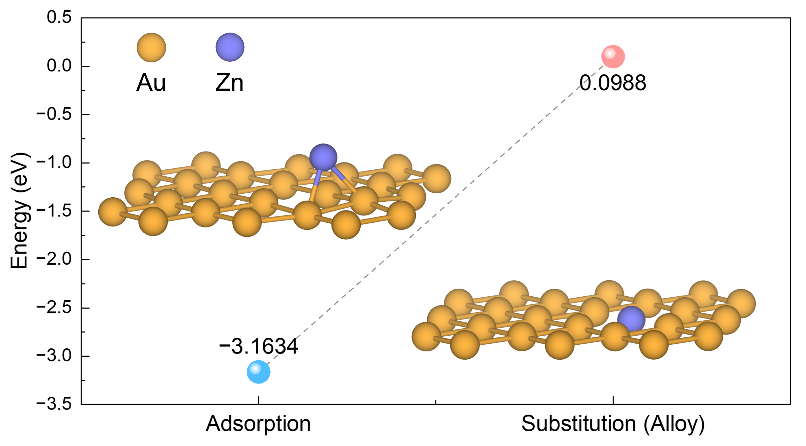


**Figure S23.** Zn adsorption energy (single Zn atom on Au nanoseed) and substitution formation energy for alloying (Zn replacing a surface Au atom; referenced to bulk Zn and Au).


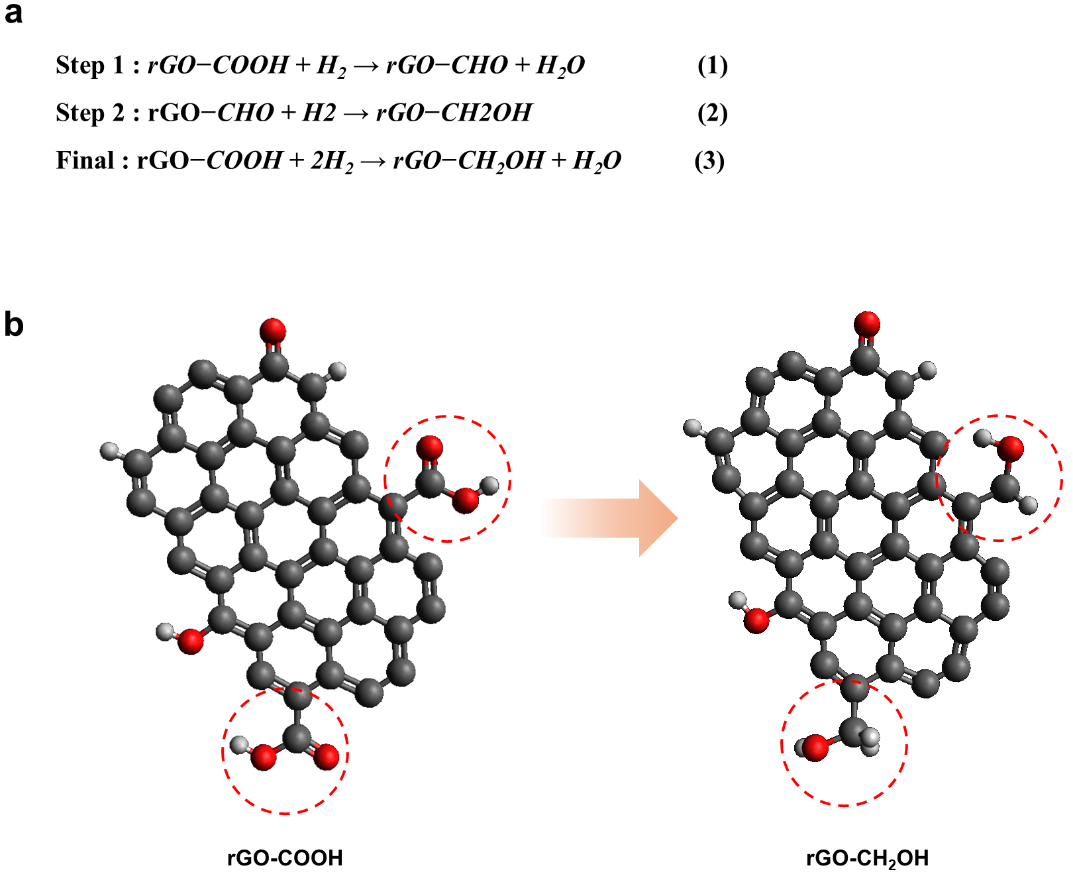


**Figure S24**. (a) Reduction pathway of the rGO surface carboxyl group (−COOH) considered for Gibbs free-energy evaluation. (b) Optimized geometries for the rGO surface −COOH to −CH₂OH reduction reaction, used in Gibbs free energy calculations.


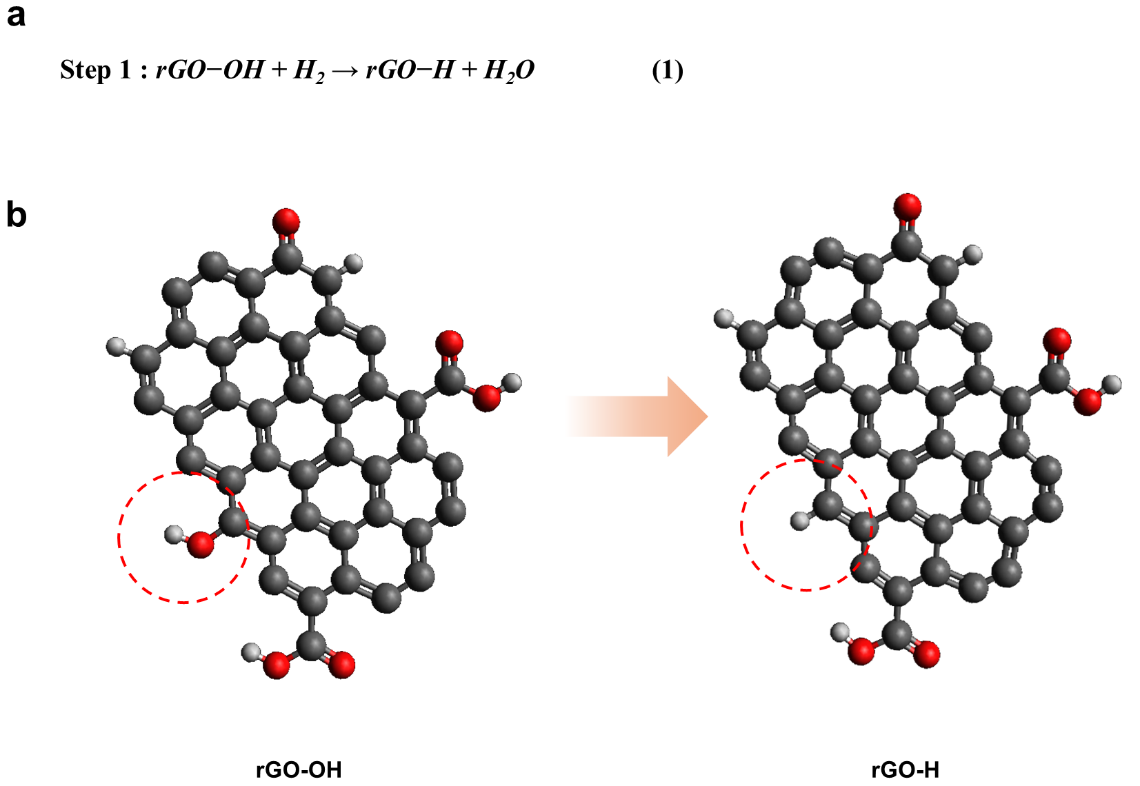


**Figure S25**. (a) Reduction pathway of the rGO surface hydroxyl group (−OH) considered for Gibbs free-energy evaluation. (b) Optimized geometries for the rGO surface −OH to −H reduction reaction, used in Gibbs free energy calculations.


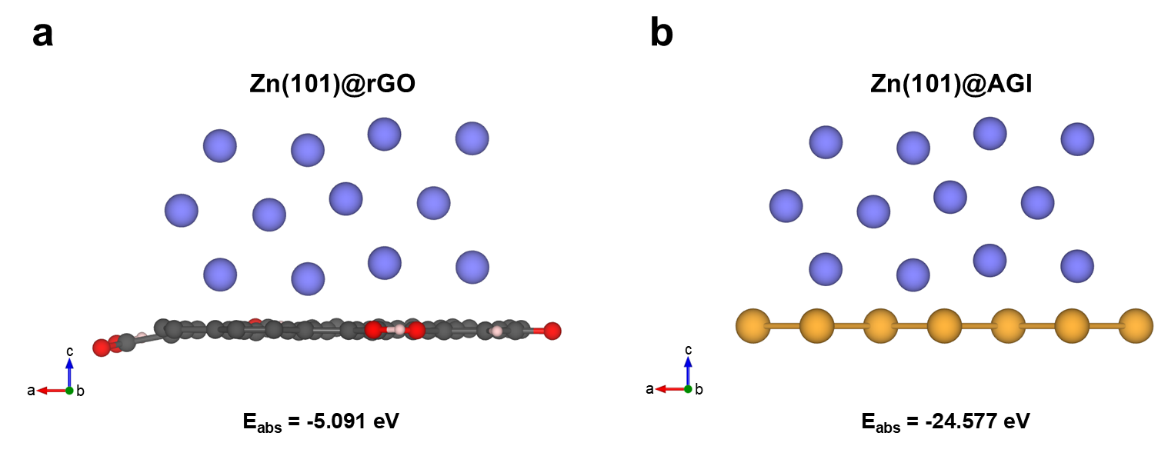


**Figure S26**. (a) Illustrations of the optimized geometries of multi-layer Zn(101) slabs on rGO and (b) AGI substrates, along with the corresponding adsorption energies (multi-layer denotes a 3-layer Zn slab).


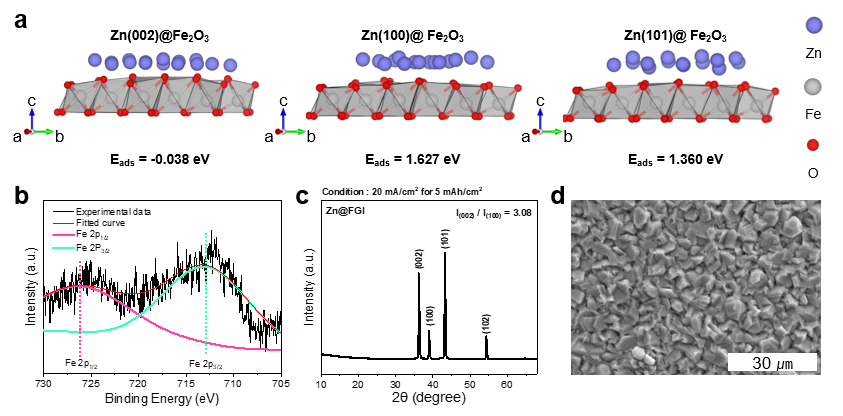


**Figure S27**. (a) Calculated adsorption energies for Zn slabs with Zn (002), Zn (100), and Zn (101) orientations on Fe_2_O_3_ substrate. (b) Deconvoluted XPS core scans of Fe 2p from Fe_2_O_3_ nanoseed-rGO interface (FGI@SUS). (c) XRD patterns of the electroplated Zn anode on FGI@SUS at 20 mA/cm^2^ and 5 mAh/cm^2^. (d) Corresponding SEM surface images of (c).


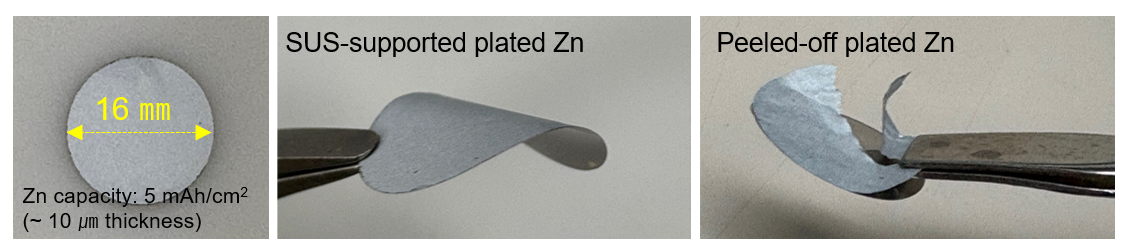


**Figure S28**. The mechanical integrity of substrate-supported Zn@AGI and peeled-off Zn film at the same areal capacity (5 mAh/cm^2^, ~10 μm) was compared, revealing severe cracking and tearing in the peeled Zn layers.


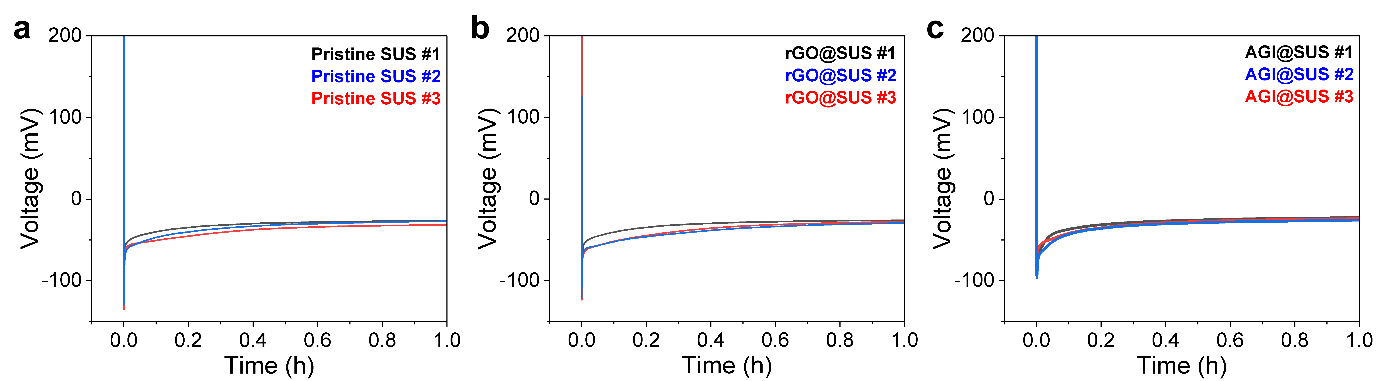


**Figure S29.** Voltage-time profiles to measure initial nucleation overpotentials by plating Zn at 1 mA/cm^2^ to 1 mAh/cm^2^ on (a) pristine SUS, (b) rGO@SUS, and (c) AGI@SUS. The average nucleation overpotential for each substrate was obtained from three independent measurements. Notably, AGI@SUS exhibited the lowest average nucleation overpotential (66.9 mV), attributed to the synergistic effect of the uniformly distributed Au nanoseed arrays and the underlying rGO nanolayer.


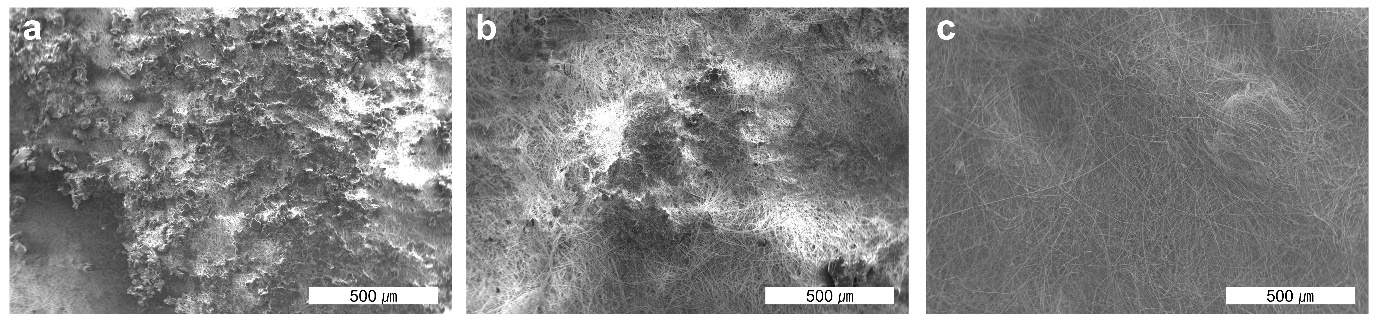


**Figure S30.** SEM images of the glass fiber separator surfaces after the short-circuit time (T_sc_) tests shown in Figure 5d. SEM images of glass fiber separators after T_sc_ tests: (a) Separator from the half-cell with pristine SUS (failure at 53 mAh/cm^2^), showing sharp Zn dendrites penetrating the separator. (b) Separator from the rGO@SUS cell (failure at 73.2 mAh/cm^2^), revealing large, irregular Zn protrusions indicative of overgrown dendrites. (c) Separator from the AGI@SUS cell after 100 mAh/cm^2^ Zn plating, exhibiting no Zn residues or structural damage, suggesting uniform Zn plating confined to AGI.


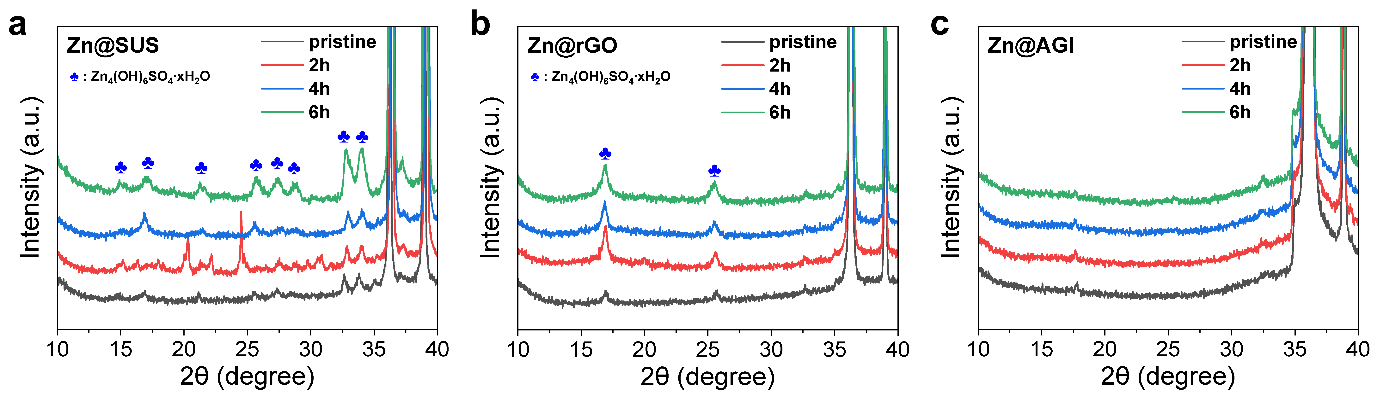


**Figure S31.** XRD patterns of different Zn anodes as a function of immersion time in 2 M ZnSO_4_ solution to compare their corrosion resistance by identifying byproduct formation such as Zn_4_(OH)_6_SO_4_·xH_2_O (ZHS). (a) Zn@SUS possessing a dominant (101) texture showed extensive formation of byproducts, indicating substantial chemical instability. (b) Zn@rGO also displayed evident ZHS formation. In contrast, (c) Zn@AGI exhibited no detectable byproducts, suggesting excellent resistance to electrolyte-induced degradation. These findings demonstrate the exceptional interfacial and chemical stability of the Zn@AGI structure under aqueous electrolyte environments.


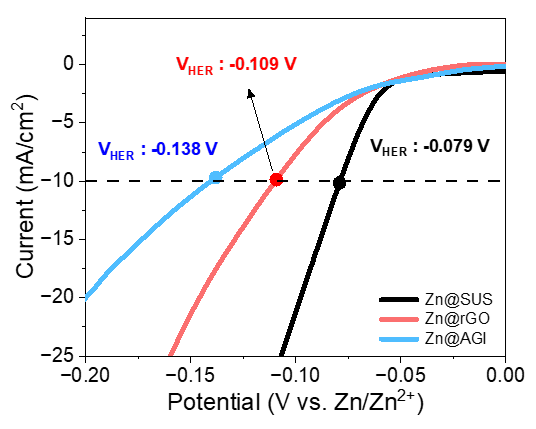


**Figure S32.** Linear sweep voltammetry (LSV) curves of Zn@SUS, Zn@rGO, and Zn@AGI in 1 M Na_2_SO_4_ electrolyte, comparing hydrogen evolution reaction (HER) activity. The HER potentials (V_HER_) were determined at a current density of -10 mA/cm^2^, as indicated by the dashed line, showing that Zn@AGI exhibits the most negative V_HER_ and thus the highest HER overpotential among the anode samples.


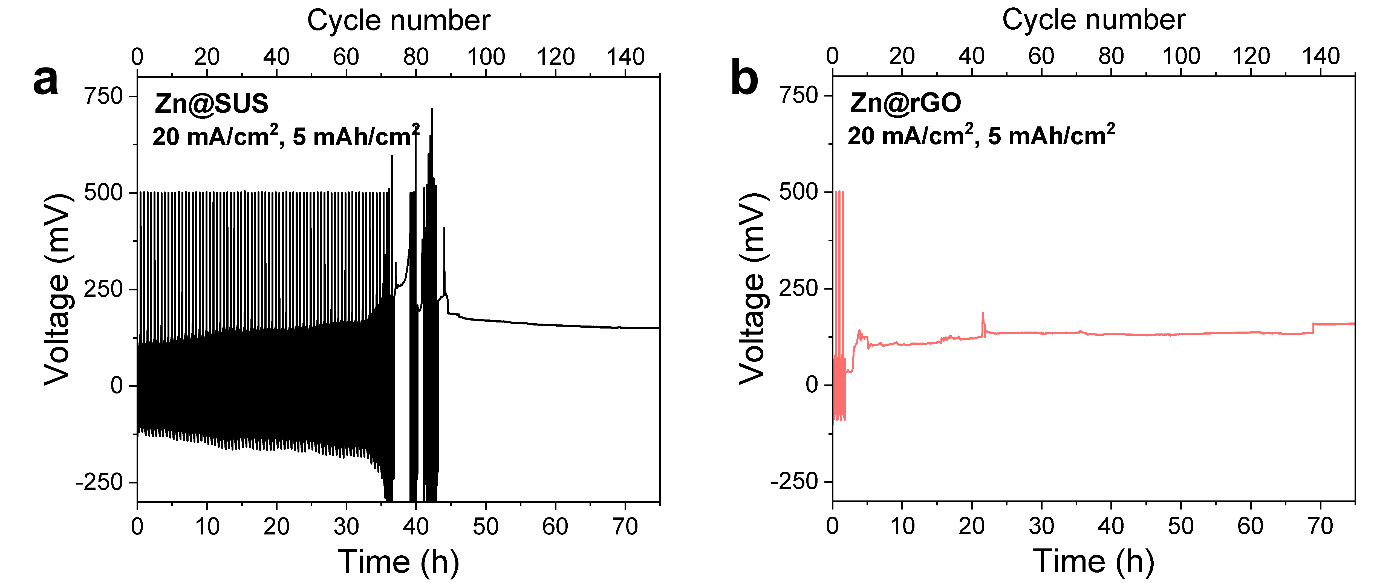


**Figure S33.** Results of asymmetric cell tests conducted at 20 mA/cm^2^ with a capacity of 5 mAh/cm^2^. (a) The Zn@SUS electrode maintained stable cycling performance for up to 35 h before failure. (b) In contrast, the Zn@rGO electrode failed after just the fourth cycle, which is attributed to side reactions between the plated Zn and oxygen-containing functional groups on the rGO surface that accelerate cycling instability.


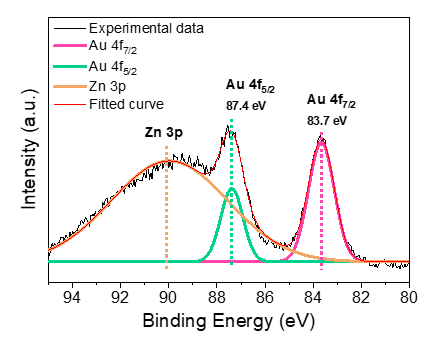


**Figure S34.** XPS spectra of the Au 4f and Zn 3p regions for AGI after Zn stripping, showing the unchanged Au 4f_7/2_ peak position and the emergence of a Zn 3p signal.


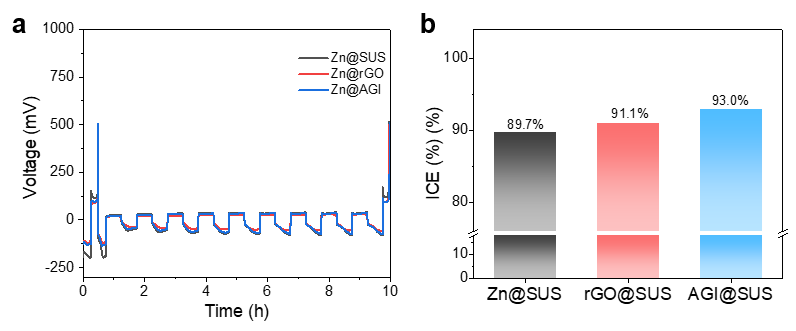


**Figure S35.** Aurbach-type Coulombic efficiency evaluation of Zn anodes using Zn plated on different substrates. Zn was electrochemically plated onto SUS, rGO@SUS, and AGI@SUS substrates, which were then used as the working electrodes, while Zn foil served as the counter/reference electrode. The cells were assembled using 2 M ZnSO_4_ electrolyte (100 µL). An initial Zn deposition of 5 mAh/cm^2^ was carried out at a current density of 20 mA/cm^2^, followed by complete stripping to a cutoff voltage of 0.5 V to establish a uniform initial state. Subsequently, repeated Zn plating/stripping cycles were performed at 1 mA/cm^2^ with a fixed areal capacity of 0.5 mAh/cm^2^ for nine cycles, with each stripping step terminated at 0.5 V. This Aurbach-type protocol allows quantitative determination of irreversible Zn loss and intrinsic reversibility of Zn anodes on different substrates. (a) Cycling profiles and (b) comparison of initial Coulombic efficiencies (ICE) for different Zn anodes.


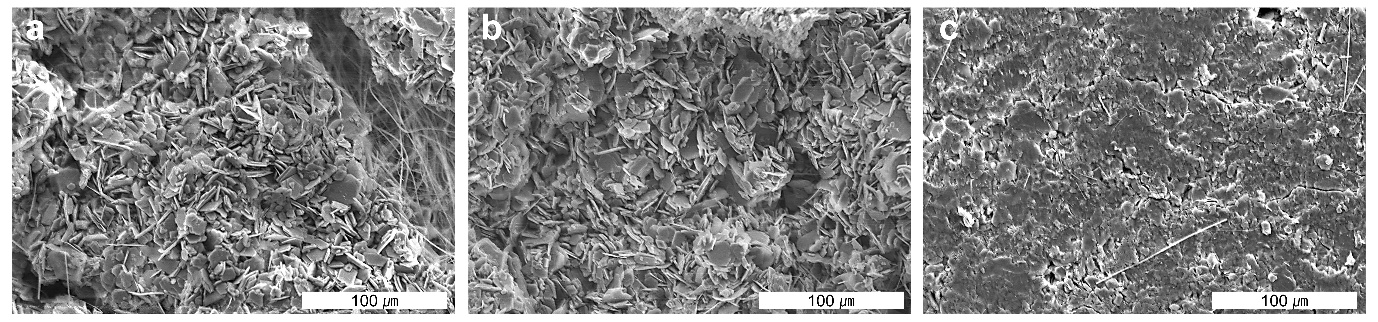


**Figure S36.** SEM surface images of (a) Zn@SUS, (b) Zn@rGO, and (c) Zn@AGI after 50 cycles at 30% DOD under 1 mA/cm^2^ and 1.5 mAh/cm^2^ conditions.


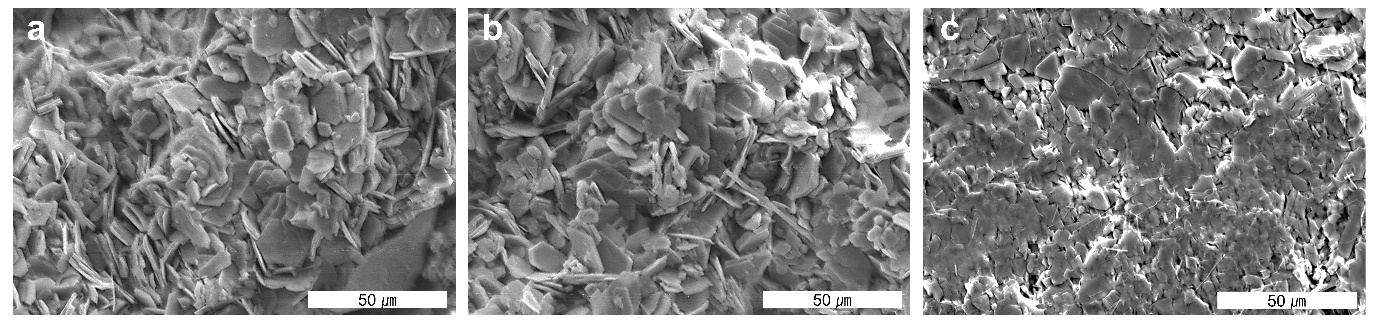


**Figure S37.** SEM surface images of (a) Zn@SUS, (b) Zn@rGO, and (c) Zn@AGI after 20 cycles at 50% DOD under 2.5 mA/cm^2^ and 2.5 mAh/cm^2^ conditions.


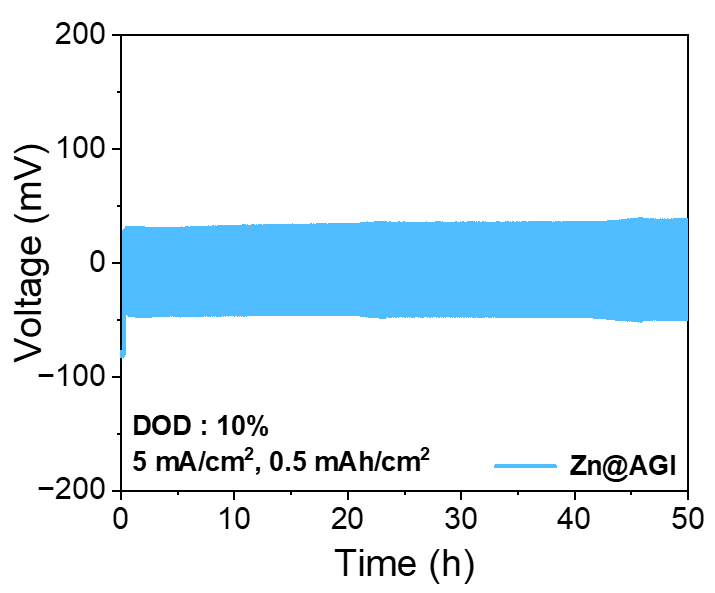


**Figure S38.** Cycling performance of symmetric cells operated at 10% DOD under 5 mA/cm^2^ and 0.5 mAh/cm^2^ conditions.


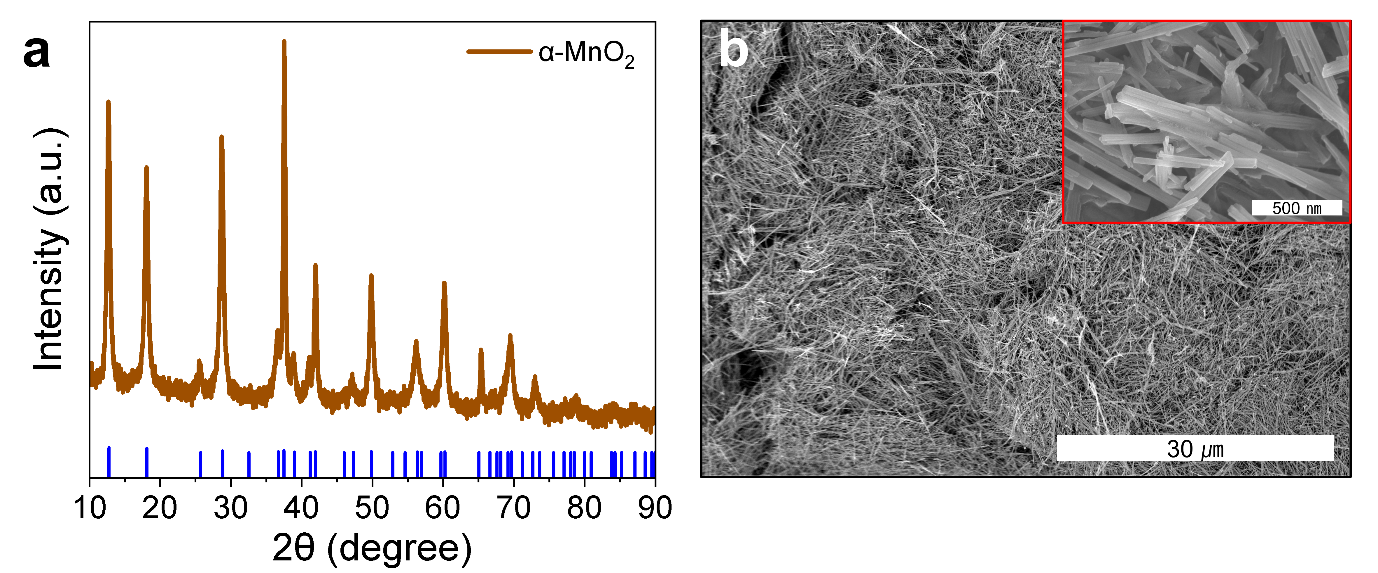


**Figure S39.** Structural and morphological characterization of synthesized α-MnO_2_. (a) XRD pattern confirming its characteristic crystal structure. (b) SEM image revealing its nanorod-type morphology.


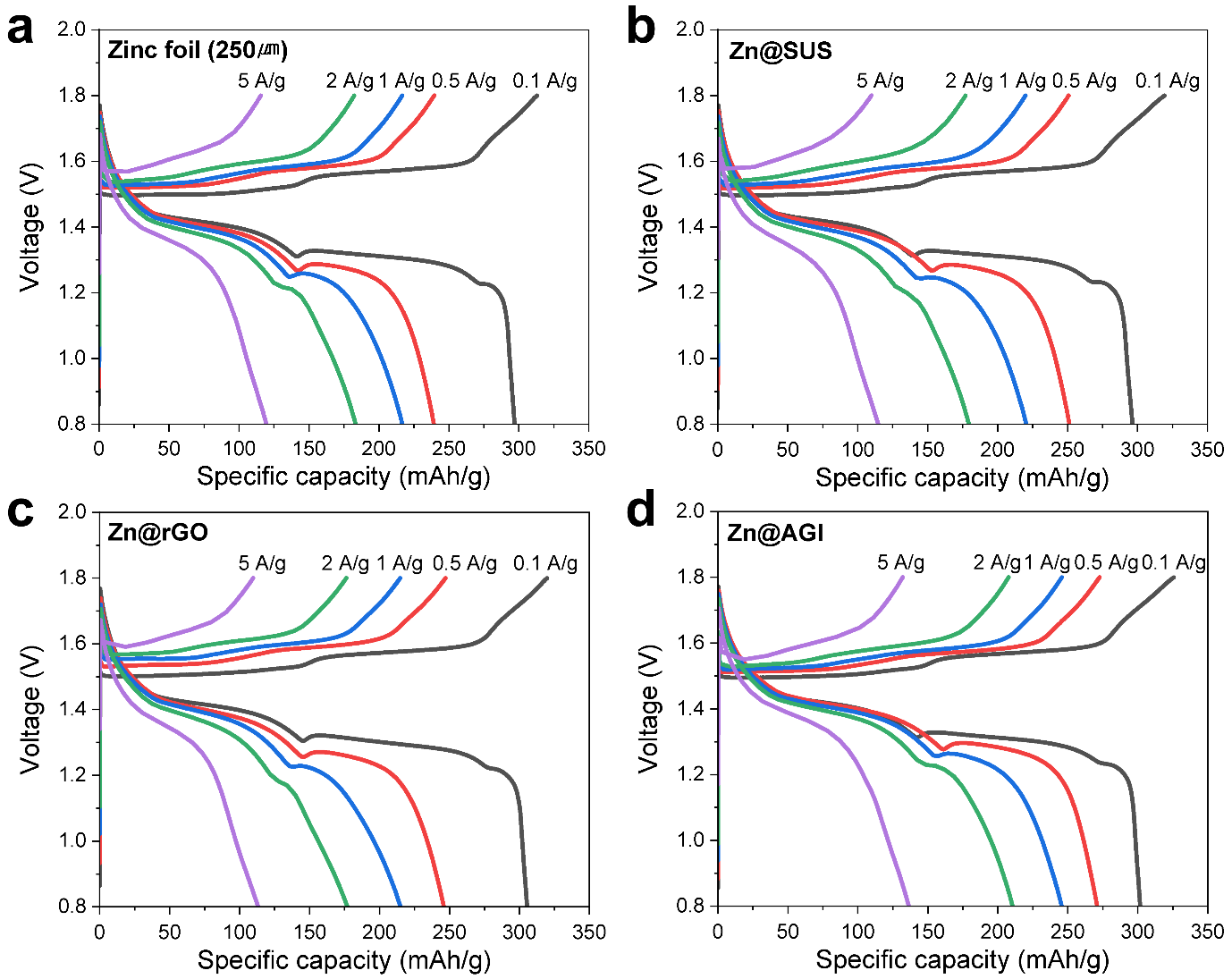


**Figure S40.** Selected voltage profiles of full cells employing (a) Zn foil, (b) Zn@SUS, (c) Zn@rGO, and (d) Zn@AGI as anodes, measured at various current densities of 0.1, 0.5, 1, 2, and 5 A/g. The MnO_2_ cathode loading was fixed at 1 mg/cm^2^.


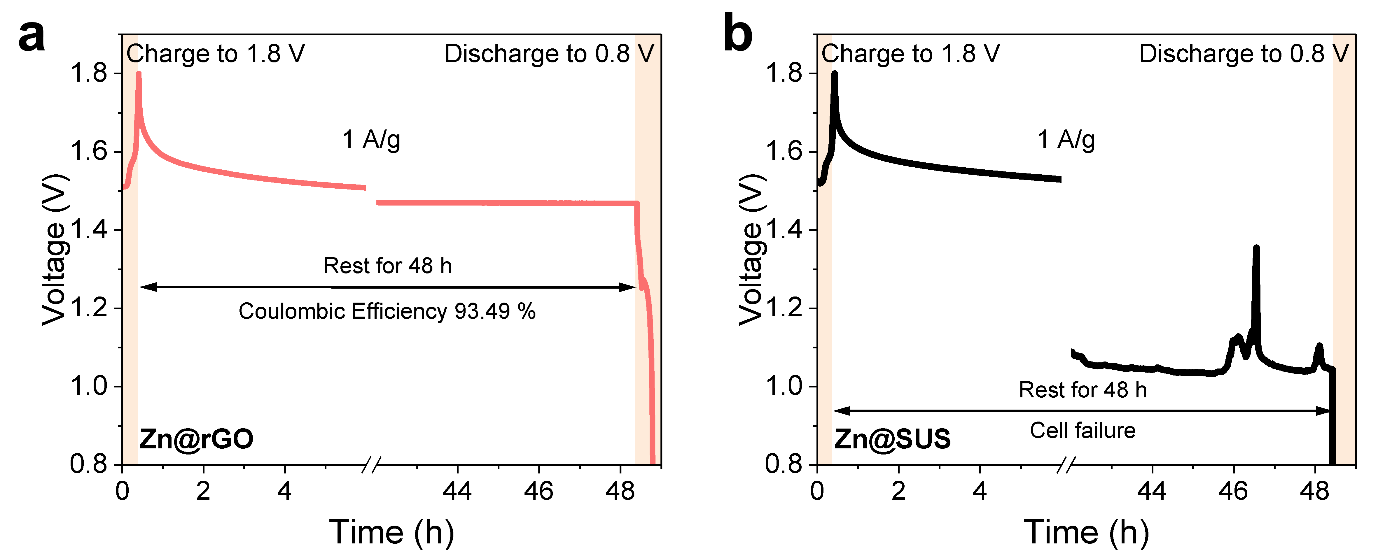


**Figure S41.** Self-discharge voltage profiles of full cells with (a) Zn@rGO and (b) Zn@SUS after charging to 1.8 V, 48 h rest, and discharge to 0.8 V.


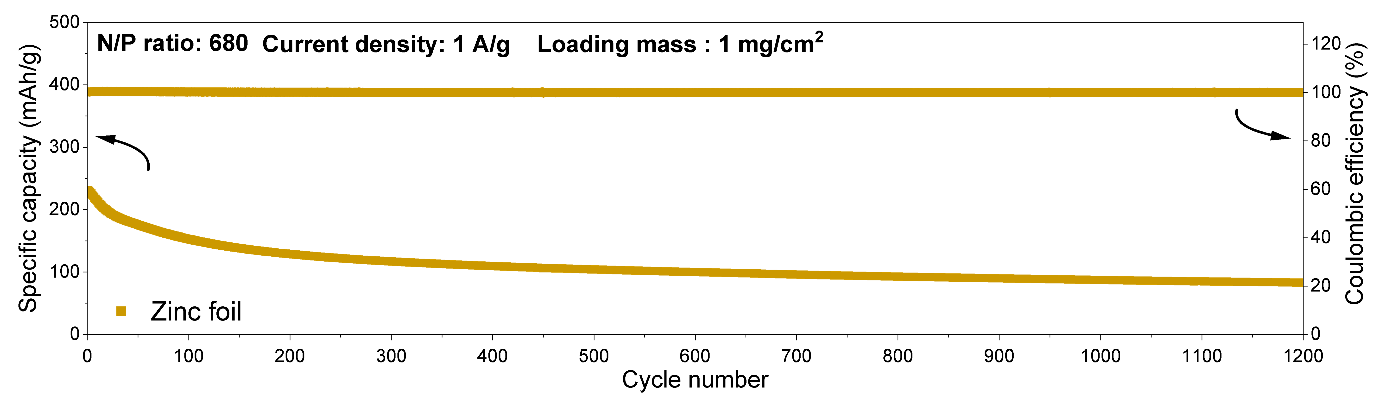


**Figure S42.** Cycling performance test of the full-cell using a 250 μm-thick zinc foil as the anode. The N/P ratio of the full-cell is as high as 680.


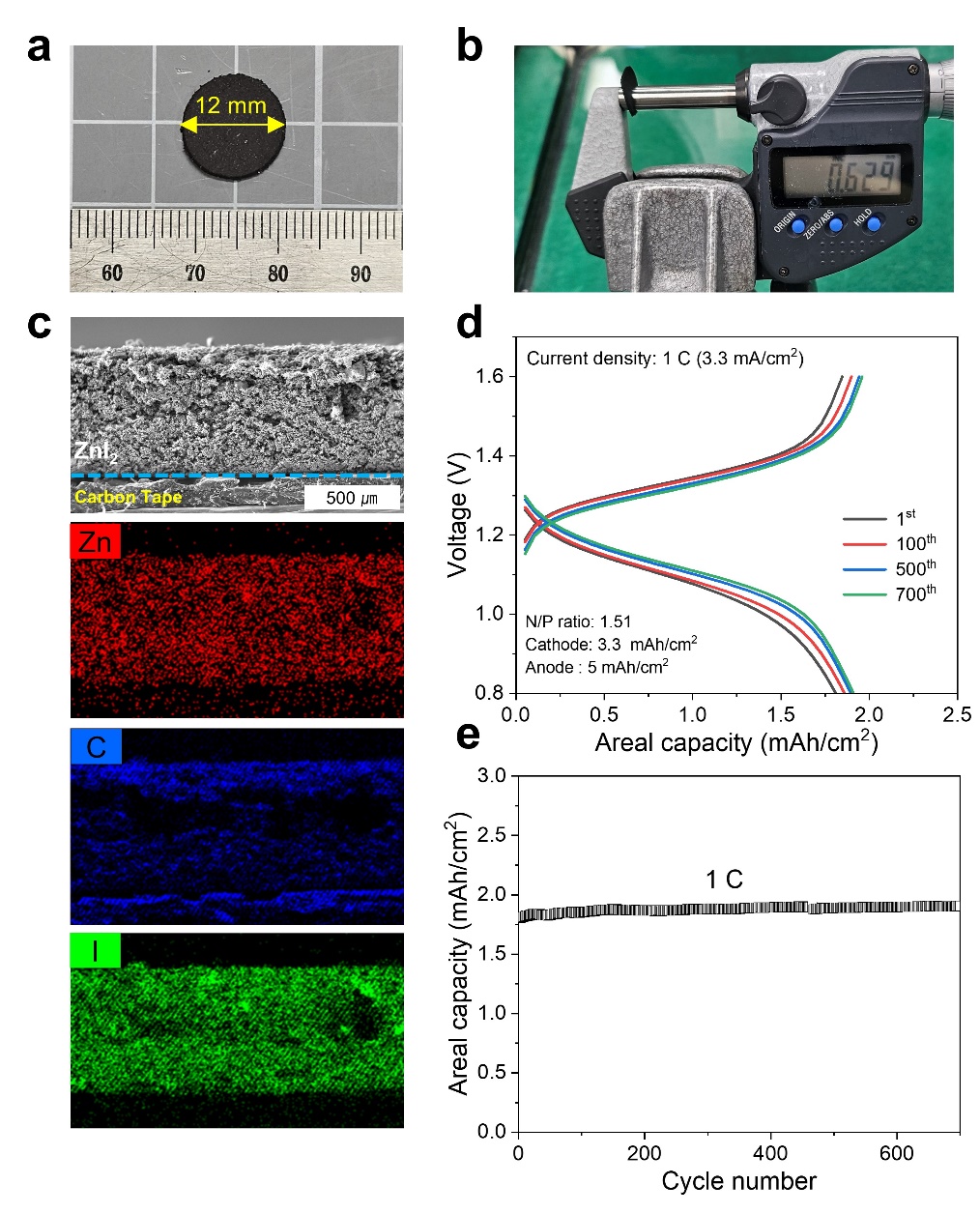


**Figure S43.** By pairing the ZnI_2_ cathode and the Zn@AGI anode, a full-cell was assembled to examine the intrinsic stability of the Zn@AGI anode under practically relevant conditions. The ZnI_2_ cathode (ZnI_2_/Activated carbon/PTFE = 49/50/1, wt%) was fabricated via a scalable dry process to achieve a high areal capacity (~3.3 mAh/cm^2^) and a lean N/P ratio of 1.51. The full-cell was evaluated at 1 C after one cycle at 0.1 C. Digital camera images of (a) the ZnI_2_ cathode prepared by the dry process and (b) showing the thickness of the fabricated ZnI_2_ cathode (~ 629 µm). (c) Cross-sectional SEM image and corresponding EDS elemental mapping of the ZnI_2_ cathode. Charge–discharge profiles of the ZnI_2_‖Zn@AGI full-cell at 0.1 and 1 C are shown in (d), and the cycling performance measured at 1 C is presented in (e). Notably, the ZnI_2_‖Zn@AGI full cell exhibited highly stable cycling performance, retaining a capacity of 1.9 mAh/cm^2^ after 700 cycles that was similar to its initial value under these demanding conditions.

**Table S1.** Work of adhesion per unit area for multilayer Zn slabs representing Zn (002), Zn (100), and Zn (101) plating on rGO and AGI substrates.

| **Multi-layer Zn slabs** | **W_ad_ = [E_(substrate)_ + E_(multi-layer Zn slabs)_ – E_(multi-layer Zn slabs + substrate)_] /A** | | |
| --- | --- | --- | --- |
|  | Zn (002) | Zn (100) | Zn (101) |
| rGO substrate | 0.4622 J/m^2^ | -0.2177 J/m^2^ | 0.3852 J/m^2^ |
| AGI substrate | 3.2856 J/m^2^ | 1.6457 J/m^2^ | 2.9027 J/m^2^ |

**Table S2.** Table summarizing the measured I_corr_ and P_corr_ values obtained from Tafel polarization test.


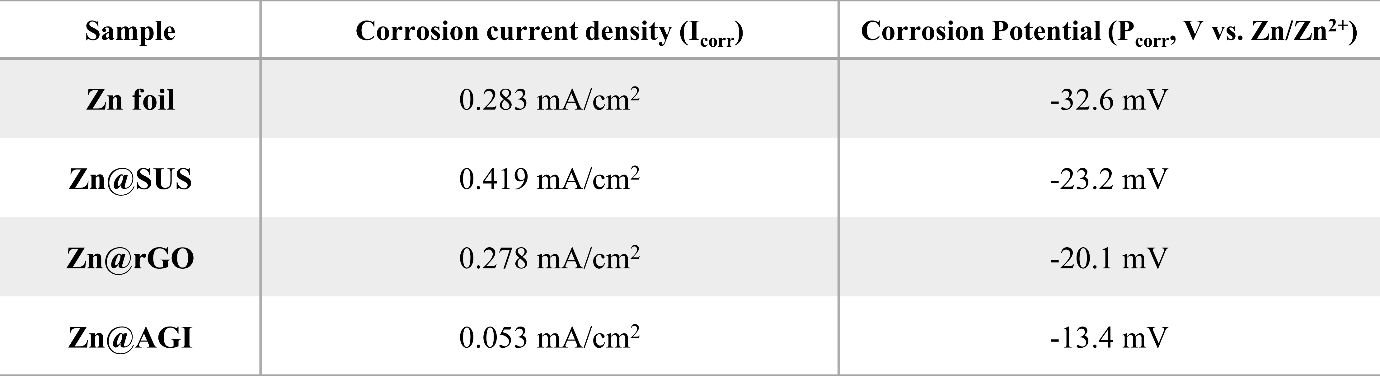


**Table S3.** Energy density comparison of AZIBs employing plated and foil-type Zn anodes at different N/P ratios.

| **Anode**  **(mAh/cm^2^)** | **Cathode** | **Electrode ratio**  **(AM^a)^ : CM^b)^: BM^c)^)** | **Electrolyte** | **N/P** | **Electrode energy density (Wh/kg)** | **Ref.** |
| --- | --- | --- | --- | --- | --- | --- |
| Zn@AGI (1.6) | α-MnO_2_ | 70 : 20 : 10 | ZnSO_4_ 2 M,  MnSO_4_ 0.1 M | 2 | 156.1 | **This work** |
| Zn@CNF (5) | V_2_O_5_ | 70 : 20 : 10 | ZnSO_4_ | 2.4 | 30.65 | [S1] |
| Zn/CNT (6.67) | MnO_2_ | 100 | ZnSO_4_ 2 M,  MnSO_4_ 0.4 M | 8.6 | 89.31 | [S2] |
| Zn@CFs (7.85) | α-MnO_2_ | 70 : 20 : 10 | ZnSO_4_ 2 M,  MnSO_4_ 0.1 M | 22.4 | 42.24 | [S3] |
| (C_2_F_4_)n-C@Cu@Zn (5) | V_2_O_5_ | 70 : 20 : 10 | Zn(OTF)_2_ 3M | 39.3 | 16.02 | [S4] |
| Zn@CuNWs (20) | Mn_0.25_V_2_O_5_ | 70 : 20 : 10 | ZnSO_4_ 2M | 101.3 | 6.116 | [S5] |
| Zn@Bio-scafold (6) | VOH | 70 : 20 : 10 | ZnSO_4_ 2M | 29.1 | 21.25 | [S6] |
| 3D Ni-Zn (8) | PVO | 70 : 20 : 10 | Zn(OTF)_2_ 3M | 38.1 | 15.02 | [S7] |
| 3D Ti-TiO_2_ (5) | S-MXene@MnO_2_ | 100 | ZnSO_4_ 2 M,  MnSO_4_ 0.2 M | 13.3 | 74.04 | [S8] |
| Zn@a-Ag mesh (2) | LFP | 70 : 20 : 10 | ZnSO_4_ 2M,  LiCl 1M | 6.8 | 52.46 | [S9] |
| Zn@ZIF-8-500 (10) | I_2_ (liquid cathode) | 80 : 20 | LiI 1M, I_2_ 0.1M,  ZnSO_4_ 0.5M | 20.8 | 33.83 | [S10] |
| MGA@Zn (5) | LMO | 70 : 20 : 1 | PVA, Mxene,  Zn(OTF)_2_, Li_2_SO_4_ | 2.7 | 81.3 | [S11] |
| Zn@LM-AgT (10) | NVO | 70 : 20 :10 | ZnSO_4_ 2 M,  MnSO_4_ 0.1 M | 46.7 | 14.65 | [S12] |
| Zn@Cu (4) | MnO_2_ | 70 : 20 : 10 | HWAE-10, ZnSO_4_ 1 M,  MnSO_4_ 0.1 M | 10 | 78.83 | [S13] |
| Zn foil (100 μm) | MnO_2_ | 100 | Acetone, ZnSO_4_ 2M | 354 | 3.42 | [S14] |
| Zn foil (113 μm) | VS_2_ | 70 : 20 :10 | La(NO_3_)_3_ 0.0085 M,  ZnSO_4_ 2 M | 6.8 | 37.79 | [S15] |
| Zn foil (50 μm) | PANI | 70 :20 :10 | TMS 30 wt%,  Zn(OTF)_2_ 2 M | 9.6 | 40.97 | [S16] |
| Zn foil (20 μm) | NH_4_V_4_O_10_ | 75 : 15 :10 | MSG 0.1 M,  ZnSO_4_ 2 M | 18.7 | 25.26 | [S17] |
| Zn foil (10 μm) | VS_2_ | 100 (Binder free) | Irgacure 2959,  ZnSO_4_ 2 M | 2.7 | 94.14 | [S18] |
| Zn foil (10 μm) | V_2_O_5_ | 70 : 20 : 10 | TMU 0.25 M,  Zn(OTF)_2_ 4 M | 14 | 36.65 | [S19] |

^a)^AM: Active material, ^b)^CM: Conductive material, ^c)^BM: Binding material

**Supporting Reference**

1. J. Li, Q. Lin, Z. Zheng, L. Cao, W. Lv, Y. Chen. How is cycle life of three-dimensional zinc metal anodes with carbon fiber backbones affected by depth of discharge and current density in zinc–ion batteries? ACS Appl. Mater. Interfaces **14**(10), 12323–12330 (2022). <https://doi.org/10.1021/acsami.2c00344>
2. Y. Zeng, X. Zhang, R. Qin, X. Liu, P. Fang, D. Zheng, Y. Tong, X. Lu. Dendrite-free zinc deposition induced by multifunctional CNT frameworks for stable flexible Zn-ion batteries. Adv. Mater. **31**(36), 1903675 (2019). <https://doi.org/10.1002/adma.201903675>
3. W. Dong, J.L. Shi, T.S. Wang, Y.X. Yin, C.R. Wang, Y.-G. Guo. 3D zinc@carbon fiber composite framework anode for aqueous Zn–MnO_2_ batteries. RSC Adv. **8**(34), 19157–19163 (2018). <https://doi.org/10.1039/c8ra03226b>
4. Q. Li, H. Wang, H. Yu, M. Fu, W. Liu, Q. Zhao, S. Huang, L. Zhou, W. Wei, X. Ji. Engineering an ultrathin and hydrophobic composite zinc anode with 24 μm thickness for high-performance Zn batteries. Adv. Funct. Mater. **33**(40), 2303466 (2023). <https://doi.org/10.1002/adfm.202303466>
5. Z. Yi, J. Liu, S. Tan, Z. Sang, J. Mao, L. Yin, X. Liu, L. Wang, F. Hou, S. X. Dou. An ultrahigh-rate and stable zinc anode by facet-matching-induced dendrite regulation. Adv. Mater. **34**(37), 2203835 (2022). <https://doi.org/10.1002/adma.202270259>
6. Z. Zhang, X. Yang, P. Li, Y. Wang, X. Zhao, J. Safaei, H. Tian, D. Zhou, B. Li, F. Kang. Biomimetic dendrite-free multivalent metal batteries. Adv. Mater. **34**(47), 2206970 (2022). <https://doi.org/10.1002/adma.202206970>
7. G. Zhang, X. Zhang, H. Liu, J. Li, Y. Chen, H. Duan. 3D-printed multi-channel metal lattices enabling localized electric-field redistribution for dendrite-free aqueous Zn-ion batteries. Adv. Energy Mater. **11**(19), 2003927 (2021). <https://doi.org/10.1002/aenm.202003927>
8. Y. An, Y. Tian, S. Xiong, J. Feng, Y. Qian. Scalable and controllable synthesis of interface-engineered nanoporous host for dendrite-free and high-rate zinc metal batteries. ACS Nano **15**(7), 11828–11842 (2021). <https://doi.org/10.1021/acsnano.1c02928>
9. R. Xue, J. Kong, Y. Wu, Y. Wang, X. Kong, M. Gong, L. Zhang, X. Lin, D. Wang. Highly reversible zinc metal anodes enabled by a three-dimensional silver host for aqueous batteries. J. Mater. Chem. A **10**(18), 10043–10050 (2022). <https://doi.org/10.1039/d2ta00326k>
10. Z. Wang, J. Huang, Z. Guo, X. Dong, Y. Liu, Y. Wang, Y. Xia. A metal–organic framework host for highly reversible dendrite-free zinc metal anodes. Joule **3**(5), 1289–1300 (2019). <https://doi.org/10.1016/j.joule.2019.02.012>
11. J. Zhou, M. Xie, F. Wu, Y. Mei, Y. Hao, L. Li, R. Chen. Encapsulation of metallic Zn in a hybrid MXene/graphene aerogel as a stable Zn anode for foldable Zn-ion batteries. Adv. Mater. **34**(1), 2106897 (2022). <https://doi.org/10.1002/adma.202106897>
12. H. Chen, Z. Guo, H. Wang, W. Huang, F. Pan, Z. Wang. A liquid metal interlayer for boosted charge transfer and dendrite-free deposition toward high-performance Zn anodes. Energy Storage Mater. **54**, 563–569 (2023). <https://doi.org/10.1016/j.ensm.2022.11.013>
13. Z. Hou, H. Tan, Y. Gao, M. Li, Z. Lu, B. Zhang. Tailoring desolvation kinetics enables stable zinc metal anodes. J. Mater. Chem. A **8**(37), 19367–19374 (2020). <https://doi.org/10.1039/d0ta06622b>
14. X. Shi, J. Wang, F. Yang, X. Liu, Y. Yu, X. Lu. Metallic zinc anode working at 50 and 50 mAh cm^-2^ with high depth of discharge via electrical double layer reconstruction. Adv. Funct. Mater. **33**(7), 2211917 (2023). <https://doi.org/10.1002/adfm.202211917>
15. R. Zhao, H. Wang, H. Du, Y. Yang, Z. Gao, L. Qie, Y. Huang. Lanthanum nitrate as aqueous electrolyte additive for favourable zinc metal electrodeposition. Nat. Commun. **13**(1), 3252 (2022). <https://doi.org/10.1038/s41467-022-30939-8>
16. Z. Shen, J. Mao, G. Yu, W. Zhang, S. Mao, W. Zhong, H. Cheng, J. Guo, J. Zhang, Y. Lu. Electrocrystallization regulation enabled stacked hexagonal platelet growth toward highly reversible zinc anodes. Angew. Chem. Int. Ed. **135**(11), e202218452 (2023). <https://doi.org/10.1002/anie.202218452>
17. Y. Zhong, Z. Cheng, H. Zhang, J. Li, D. Liu, Y. Liao, J. Meng, Y. Shen, Y. Huang. Monosodium glutamate, an effective electrolyte additive to enhance cycling performance of Zn anode in aqueous battery. Nano Energy **98**, 107220 (2022). <https://doi.org/10.1016/j.nanoen.2022.107220>
18. T. C. Li, C. Lin, M. Luo, P. Wang, D.S. Li, S. Li, J. Zhou, H. Y. Yang. Interfacial molecule engineering for reversible Zn electrochemistry. ACS Energy Lett. **8**(8), 3258–3268 (2023). <https://doi.org/10.1021/acsenergylett.3c00859>
19. J. Yang, Y. Zhang, Z. Li, X. Xu, X. Su, J. Lai, Y. Liu, K. Ding, L. Chen, Y. P. Cai. Three birds with one stone: tetramethylurea as electrolyte additive for highly reversible Zn-metal anode. Adv. Funct. Mater. **32**(49), 2209642 (2022). <https://doi.org/10.1002/adfm.202209642>
